# An Iterative Divergent Approach to Conjugated Starburst Borane Dendrimers

**DOI:** 10.1002/chem.202001985

**Published:** 2020-09-16

**Authors:** Florian Rauch, Peter Endres, Alexandra Friedrich, Daniel Sieh, Martin Hähnel, Ivo Krummenacher, Holger Braunschweig, Maik Finze, Lei Ji, Todd B. Marder

**Affiliations:** ^1^ Institut für Anorganische Chemie and Institute for Sustainable Chemistry & Catalysis with Boron (ICB) Julius-Maximilians-Universität Würzburg Am Hubland 97074 Würzburg Germany; ^2^ Frontiers Science Center for Flexible Electronics (FSCFE) Shaanxi Institute of Flexible Electronics (SIFE) & Shaanxi Institute of Biomedical Materials and Engineering (SIBME) Northwestern Polytechnical University 127 West Youryi Road 710072 Xi'an China

**Keywords:** density functional calculations, electron storage, luminescence, redox, triarylborane

## Abstract

Using a new divergent approach, conjugated triarylborane dendrimers were synthesized up to the 2^nd^ generation. The synthetic strategy consists of three steps: 1) functionalization, via iridium catalyzed C−H borylation; 2) activation, via fluorination of the generated boronate ester with K[HF_2_] or [N(nBu_4_)][HF_2_]; and 3) expansion, via reaction of the trifluoroborate salts with aryl Grignard reagents. The concept was also shown to be viable for a convergent approach. All but one of the conjugated borane dendrimers exhibit multiple, distinct and reversible reduction potentials, making them potentially interesting materials for applications in molecular accumulators. Based on their photophysical properties, the 1^st^ generation dendrimers exhibit good conjugation over the whole system. However, the conjugation does not increase further upon expansion to the 2^nd^ generation, but the molar extinction coefficients increase linearly with the number of triarylborane subunits, suggesting a potential application as photonic antennas.

## Graphical abstract

**Boron branches out**: Conjugated starburst borane dendrimers were synthesized up to the 2^nd^ generation, using to a new, divergent, three‐step approach, exhibiting good substrate tolerance. The dendrimers exhibit multiple, distinct and reversible reduction potentials, making them potential materials for molecular accumulators. Photophysical measurements indicate good conjugation up to the 1^st^ generation.

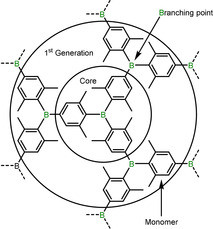

## Introduction

**Scheme 1:**
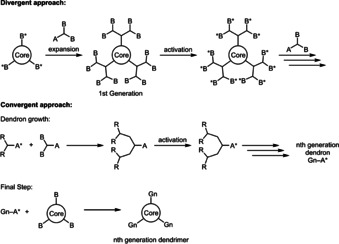
General approaches to dendrimer synthesis. A, B and A^*^ and B^*^ indicate different end groups. Gn indicates the generation of the dendrimers.

Three‐coordinate boranes have attracted considerable interest due to their inherent and interesting properties.^1^, ^2^, ^3^, ^4^, ^5^, ^6^, ^7^, ^8^, ^9^, ^10^, ^11^, ^12^, ^13^ Three‐coordinate boron is isoelectronic with a carbonium ion, and has an empty *p*‐orbital. Thus, boranes can be employed as Lewis acids or electron acceptors. Therefore, a plethora of different potential applications have been investigated, including linear^14^, ^15^, ^16^, ^17^, ^18^, ^19^, ^20^, ^21^, ^22^, ^23^, ^24^, ^25^, ^26^, ^27^, ^28^, ^29^, ^30^, ^31^, ^32^, ^33^, ^34^ and non‐linear^35^, ^36^, ^37^, ^38^, ^39^, ^40^, ^41^, ^42^, ^43^, ^44^, ^45^, ^46^, ^47^ optics, bioimaging,^30^, ^46^, ^47^, ^48^, ^49^ sensors,^7^, ^50^, ^51^, ^52^ frustrated Lewis pairs (FLPs),^53^, ^54^, ^55^, ^56^, ^57^, ^58^, ^59^ and organic light emitting diodes (OLEDs).^60^, ^61^, ^62^ Dendrimers represent a class of macromolecules which, as opposed to linear and hyperbranched polymers, can be isolated as monodisperse compounds. As such, dendrimers provide potential alternatives to polymers in areas where monodispersity is essential, and they have been studied extensively for applications in a wide variety of fields.^63^, ^64^, ^65^, ^66^, ^67^, ^68^, ^69^, ^70^, ^71^, ^72^, ^73^, ^74^, ^75^ Due to their attachment to a focal point, dendrimers usually adopt a spherical structure, especially at higher generations. Thus, their chemistry and properties are often dominated by their outermost layer. This is not the case for conjugated dendrimers, as the goal is to generate large systems with a high degree of *π*‐electron delocalization. These systems show promising properties for application such as charge transport^76^, ^77^, ^78^, ^79^, ^80^ or emitter materials^81^, ^82^, ^83^, ^84^, ^85^, ^86^, ^87^, ^88^ in OLEDs, or as electron‐acceptor or ‐donor materials in organic solar cells.^89^, ^90^, ^91^ There are generally two approaches to dendrimer synthesis, namely convergent and divergent (Scheme 1).

**Figure 1:**
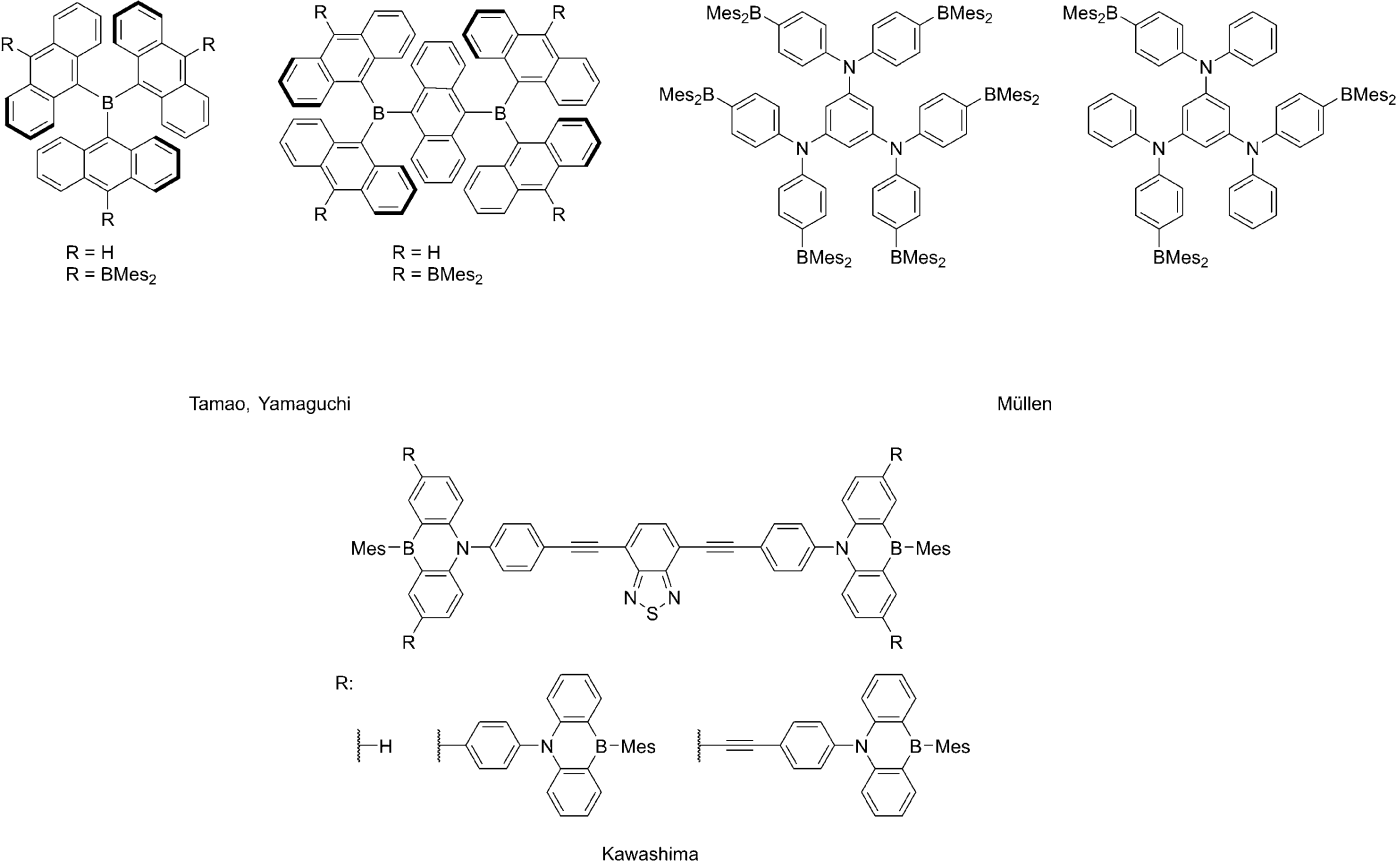
Examples of previously synthesized conjugated borane dendrimers.^15^, ^97^, ^98^

In the convergent approach, the dendrimer is built up concentrically from the focal point or core moiety, adding one generation at a time. The drawback of this method is that the number of active centers needed for the expansion, increases exponentially with each generation. This also increases the probability of defects. In the convergent approach, the dendrons are synthesized up to a desired generation, and then attached to the focal point in the last step. The drawback of this strategy is the last step, which needs to be highly efficient in order to warrant the effort of first synthesizing the dendrons. However, the probability of defects is lower using this approach. There are several examples of boron‐containing dendrimers.^15^, ^92^, ^93^, ^94^, ^95^, ^96^, ^97^, ^98^ However, the majority are dendrimers with a boron‐functionalized periphery. The number of conjugated dendrimers reported which contain boron as a structural component is limited (Figure 1).^15^, ^97^, ^98^, ^99^

The first example, reported by Tamao, Yamaguchi, and co‐workers, was synthesized by a convergent approach (Figure 1, top left).^15^ Tri‐9‐anthrylborane was used as the core and expanded with dimesitylborane (BMes_2_). The dendrimers show a red shift of their absorption bands as well as a rise of their molar extinction coefficients and an anodic shift of their first reduction potentials with increasing dendrimer size, indicating good conjugation throughout the system. The reduction events can be attributed to reduction of the boron centers. The boron‐containing dendrimers reported by Müllen and co‐workers contain an electron‐rich arylamine core with BMes_2_ moieties as the outer periphery (Figure 1, top right).^97^ Changing the ratio of boron and nitrogen changes the excited state properties of those dendrimers. In a third example, reported by Kawashima and co‐workers, azaborinines were used as the branching points of the dendrons.^98^ However, due to a perpendicular arrangement of the azaborinine centers with respect to each other and to the focal point, conjugation seems to be limited, and the dendrimers show very similar properties to the dendrons themselves.

**Scheme 2:**
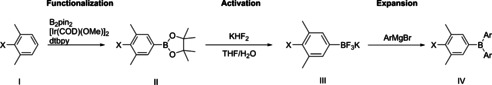
Divergent approach for the synthesis of conjugated starburst borane dendrimers.

Based on these systems, and our own expertise, we designed a new iterative, divergent methodology for the synthesis of conjugated starburst borane dendrimers (Scheme 2).

Our method consists of three steps: functionalization, activation and expansion. Tris(2,6‐dimethylphenyl)borane (**BG0H**) was chosen as the core moiety, as it exhibits a very high degree of stability due to the steric protection of the boron center by six *ortho*‐methyl groups. In the first step (functionalization), the xylene moieties were regioselectively borylated *para* to the boron center via iridium‐catalyzed C−H borylation.^100^, ^101^ This catalytic system has been studied extensively and is highly efficient and regioselective for sterically‐demanding aromatic substrates. This is also a particularly convenient approach, as the boron in the center of the dendrimer needs to be sterically protected in order to achieve stability towards nucleophiles (hydrolysis). In the second step (activation), the arylboronate ester was converted to the corresponding potassium trifluoroborate salt with K[HF_2_]. This transformation is also generally very efficient. Aryltrifluoroborate salts are highly stable compounds and are widely used in cross‐coupling reactions.^102^, ^103^ Despite their high stability and anionic charge, we and others have found that [aryl‐BF_3_]K salts can be employed as convenient electrophiles for the synthesis of triarylboranes.^29^, ^31^, ^32^, ^34^, ^104^, ^105^, ^106^, ^107^, ^108^, ^109^, ^110^ Thus, in the third step (expansion), all boron centers are arylated using the 2,6‐dimethylphenyl (Xyl) Grignard reagent. It is important to note that this method was designed to be generally applicable to 2,6‐funtionalized phenyl groups. Herein we present the results of this study.

## Results and Discussion

### Synthesis

The nomenclature for the dendrimers was chosen as follows: **BG** denotes dendrimers, **BD** denotes dendrons, the subsequent number denotes the generation, and the last character(s) denote the substituent *para* to the boron center on the outermost layer.

**Scheme 3:**
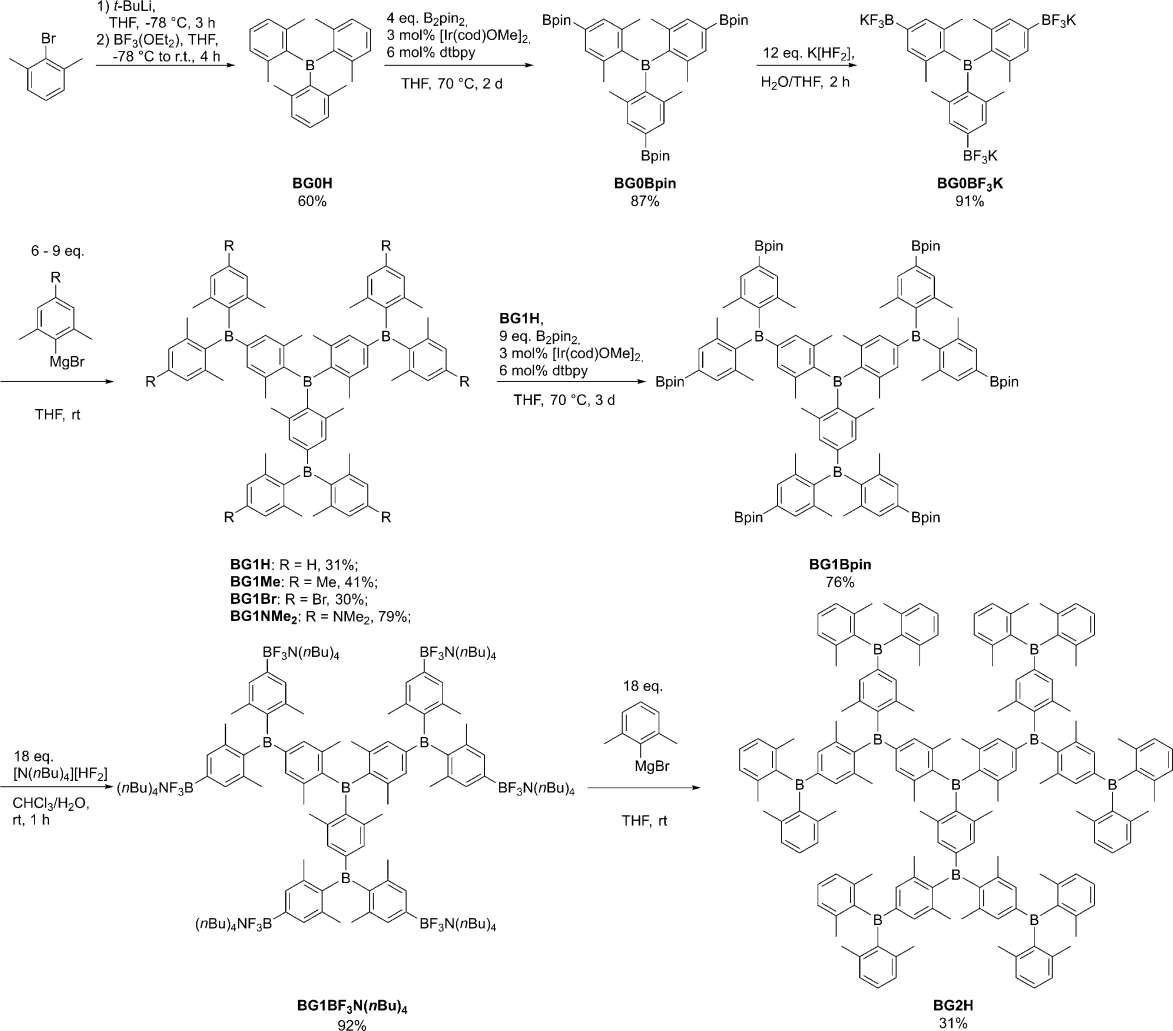
Divergent synthesis of conjugated borane dendrimers **BG1H**, **BG1Me**, **BG1Br**, **BG1NMe_2_**, BG1Bpin, **BG1BF_3_N(*n*Bu)_4_**, and **BG2H**.

The divergent synthesis is depicted in Scheme 3. All compounds were fully characterized by 1D and 2D NMR spectroscopy, high resolution mass spectrometry and, when possible, by X‐ray crystallography (see Supporting Information (SI)).

The core moiety **BG0H** was synthesized according to a literature procedure and was isolated in a good yield.^27^ **BG0H** was subsequently borylated with [Ir(COD)OMe]_2_ (COD=cyclooctadiene) as the catalyst precursor, di‐*tert*‐butylbipyridine (dtbpy) as the ligand, bis(pinacolato)diboron (B_2_pin_2_) as the boron source, and THF as the solvent. Then, **BG0Bpin** was fluorinated using KHF_2_ in THF/H_2_O to form the activated core moiety **BG0BF_3_K**. Both **BG0Bpin** and **BG0BF_3_K** were isolated in very good yields on a multi‐gram scale with a very simple work‐up. However, it was apparent that the solubility of **BG0BF_3_K** in polar organic solvents is quite low. As **BG0BF_3_K** is only slightly soluble in THF, a suspension was reacted with an excess of 2,6‐dimethylphenylmagnesium bromide (9 equiv. instead of 6 equiv.), and **BG1H** was isolated in 31 % yield. Yields of arylation reactions of the aryl‐BF_3_K salts were found to be strongly dependent on the substituents on the aryl moiety, as electron withdrawing substituents increase the stability of the trifluoroborate salts whereas electron donating substituents decrease their stability.^34^ It is likely that both the low solubility, as well as the electron withdrawing nature of the boron substituents are responsible for the modest yield. In order to test the functional group tolerance and the influence of the substituent *para* to the boron center on the outermost layer, we also synthesized the 1^st^ generation derivatives **BG1Me**, **BG1Br**, and **BG1NMe_2_** with methyl, bromide, and dimethylamino substituents, respectively. The syntheses were analogous to that used for the preparation of **BG1H**, but the respective Grignard reagents were employed. The dendrimers **BG1Me**, **BG1Br**, and **BG1NMe_2_** were isolated in moderate to good yields. **BG1H** was purified without the use of column chromatography simply by precipitation from CHCl_3_ with EtOH. This procedure did not work as well for **BG1Me**, **BG1Br**, and **BG1NMe_2_**; therefore, those products were purified by column chromatography. To synthesize the 2^nd^ generation dendrimer, **BG1H** was borylated analogously to **BG0H**, and **BG1Bpin** was isolated in a very good yield. However, **BG1Bpin** is almost insoluble in most polar or non‐polar organic solvents, with CHCl_3_ being the exception. This became problematic for the activation step, and several attempts to synthesize **BG1BF_3_K** in various solvent mixtures (THF/H_2_O, MeOH/H_2_O, CHCl_3_/H_2_O, and toluene/H_2_O), or with the addition of a crown ether (18‐C‐6) or phase transfer catalysts ([N(*n*Bu)_4_]Cl, [N(*n*Bu)_4_]Br, [N(*n*Bu)_4_]OH), were unsuccessful. Finally, exchange of the cation of the fluorination agent from potassium to tetrabutylammonium, allowed the isolation of **BG1BF_3_N(*n*Bu)_4_** in a very good yield. **BG1BF_3_N(*n*Bu)_4_** is very soluble in a variety of polar organic solvents (especially CH_2_Cl_2_) and almost insoluble in water. However, it was unclear what influence the exchange of the cation would have on the expansion step. We have previously proposed that exchanging potassium for lithium increases the reactivity due to the thermodynamically more favorable formation of LiF compared to KF.^109^ **BG1BF_3_N(*n*Bu)_4_** was reacted with an excess of 2,6‐dimethylphenylmagnesium bromide (18 equiv. instead of 12 equiv.) in THF. It is important to note that **BG1BF_3_N(*n*Bu)_4_** does not dissolve very well in THF and forms a viscous oil. However, upon addition of the Grignard reagent, a clear solution formed. After purification by column chromatography, **BG2H** was isolated in a moderate yield, which can be attributed to the fact that **BG2H** decomposes slowly on silica gel. Unfortunately, we have not yet found a better procedure to obtain pure **BG2H**.

**Scheme 4:**
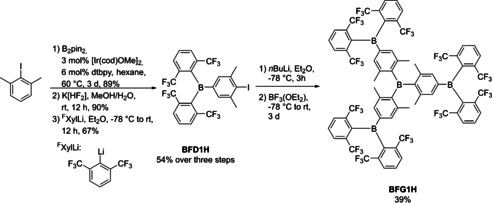
Convergent synthesis of conjugated borane dendrimer **BFG1H**.

All compounds discussed herein exhibit high apparent symmetry in solution as observed by ^1^H and ^13^C NMR spectroscopy. Only one set of resonances for each of the chemically equivalent protons and carbon atoms was observed. No ^11^B NMR signals corresponding to the three‐coordinate boron center were observed for most dendrimers in solution, due to the quadrupole moment of boron and, thus, inherent signal broadening combined with their low solubilities. As **BG0BF_3_K**, **BG1H**, and **BG1Bpin** were synthesized in larger quantities, solid‐state ^11^B NMR spectra were recorded (Figures S10, S13 and S17, respectively, in the Supporting Information). While we struggled with the synthesis of **BG1BF_3_N(*n*Bu)_4_** and **BG2H**, we searched for ways to increase the solubility of the precursors. Having previously worked with 2,6‐bis(trifluoromethyl)phenyl (^F^Xyl)‐substituted triarylboranes^109^, ^110^ which show significantly improved solubility compared to their xylyl analogues, we decided to exchange the methyl groups on the periphery of **BG1H** with trifluoromethyl groups. Initial attempts to achieve this using the above methodology failed, as the ^F^Xyl Grignard reagent exhibits rather low reactivity, and is also unstable at higher temperatures. Using ^F^XylLi was also unsuccessful, as the reaction is very slow, and ^F^XylLi decomposes in THF at room temperature. Instead, we decided to synthesize **BFG1H** via a convergent approach using the dendron **BFD1H** that we had previously reported for the synthesis of borane‐containing TADF materials (Scheme 4).^110^

Thus, **BFD1H** was synthesized according to our previously published procedure. Before preparing **BFD1H**, we had also synthesized a derivative with a bromide instead of iodide, but its lithiation was unsuccessful. Upon addition of either *n*BuLi or *t*BuLi at −78 °C the reaction changed from colorless to deep green, and upon slight warming, became dark brown. This might be due to radical intermediates formed in the bromine/lithium exchange, whereas the iodine/lithium exchange does not involve radical intermediates.^111^, ^112^ We assume that a radical species is formed which is thermally unstable.

In contrast, **BFD1H** was successfully lithiated using *n*BuLi and then reacted with BF_3_⋅OEt_2_ to give **BFG1H** in a moderate yield after purification by column chromatography. As expected, **BFG1H** is qualitatively more soluble in non‐polar and polar solvents than **BG1H. BFG1H** also exhibits apparent C_3_ symmetry in solution as observed by NMR spectroscopy. Two broad singlets are observed in the ^19^F NMR spectrum, their linewidth indicative of a hindered rotation process, as previously observed for similar boranes with *ortho*‐trifluoromethyl groups.^113^ The solid‐state ^11^B NMR spectrum of **BFG1H** shows three distinct boron signals with ^11^B NMR shifts of *δ*=77.4, 71.0 and 69.6 ppm, which integrate to approximately 1:3:3, respectively, indicative of possible geometrical isomers in the solid‐state (see below).

### Crystal and molecular structures

**Figure 2:**
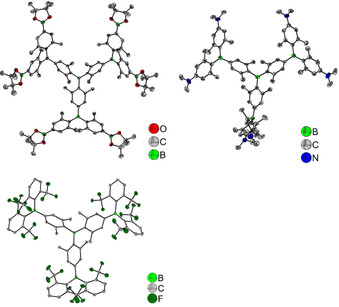
Molecular structures of **BG1Bpin** (top left), **BG1NMe_2_** (top right) and **BFG1H** (bottom left) determined by single‐crystal X‐ray diffraction at 100 K. All ellipsoids are drawn at the 50 % probability level, and H atoms and solvent molecules are omitted for clarity. For **BG1Bpin**, the Bpin moieties are slightly disordered and only the major part (94 %) is shown. The molecule has 3‐fold and 2‐fold rotation symmetries. For **BFG1H**, one of the bis(2,6‐bis(trifluoromethyl)phenyl)boranyl (B(^F^Xyl)_2_) groups is disordered and only the major part (69 %) is shown.

Single crystals suitable for X‐ray diffraction were obtained via slow liquid‐liquid diffusion, by layering saturated solutions of the dendrimer in CHCl_3_ (**BG1Bpin**) or CH_2_Cl_2_ (**BG1NMe_2_** and **BFG1H)** with ethanol (**BG1Bpin** and **BG1NMe_2_**) or hexane (**BFG1H)**. The molecular structures are shown in Figure 2 and selected bond lengths and angles are listed in Table 1.

**Table 1:** Selected angles [°], bond lengths and distances [Å] of **BG1Bpin**, **BFG1H**, and **BG1NMe_2_**.

**BG1Bpin**		**BFG1H**		**BG1NMe_2_**		
Angle [°]	B−C bond length [Å]	Angle [°]/ distance [Å]	B−C bond length/ distance [Å]	Angle [°]	B−C bond length [Å]	C−N bond length [Å]

**∡BC_3_‐Ar and B−C bond length inner layer**						
50.95(8) 3×	1.576(3) 3×	50.00(6)	1.585(2)	54.31(14)	1.579(7)	
		46.96(6)	1.576(2)	48.88(15)	1.584(7)	
		47.36(6)	1.581(2)	45.06(14)	1.572(7)	

**∡BC_3_−Ar and B−C or C−N bond length outer layer**						
23.45(12) 3×	1.566(4) 3×	34.64(6)	1.559(2)	44.39(15)	1.571(7)	1.381(6)
53.75(7) 6×	1.577(2) 6×	55.50(6)	1.608(2)	44.92(15)	1.567(7)	1.388(6)
		61.07(6)	1.602(2)	50.33(15)	1.563(7)	1.401(6)
		40.40(6)	1.558(2)	42.0(2)	1.576(7)	1.393(6)
		44.42(6)	1.604(2)	50.4(2)	1.562(8)	1.379(6)
		57.62(6)	1.604(2)	46.8(2)	1.547(8)	1.387(14)/1.446(16)
		22.94(19)/28.6(4)	1.555(2)/1.554(3)	37.67(14)	1.578(7)	
		59.07(16)/57.9(4)	1.607(2)/1.607(2)	50.93(15)	1.569(7)	
		44.90(19)/45.1(4)	1.609(2)/1.608(2)	51.19(16)	1.561(7)	

**Sum ∡CBC inner**						
360.000(1)		359.97(12)		360.0(4)		

**Sum ∡CBC outer**						
360.0(2) 3×		359.99(12)		359.9(4)		
		360.00(12)		360.0(5)		
		360.0(4)/360.0(9)		360.0(4)		

**Sum ∡CNC**						
				359.0(4)		
				359.7(5)		
				350.4(5)		
				354.5(5)		
				357.2(4)		
				360.0(14)/354.3(13)		

**Shortest B⋅⋅⋅F contacts**						
		2.935(2)	2.584(2)			
		2.954(2)	2.688(2)			
		2.925(2)	2.818(2)			
		2.871(2)	2.729(2)			
		2.770(18)/2.70(4)	2.850(9)/2.89(2)			
		2.804(14)/2.70(3)	2.928(13)/2.82(3)

The borylated 1^st^ generation dendrimer **BG1Bpin** molecule exhibits *D*_3_ symmetry in the solid state. In contrast, the central boron atoms of the molecules of **BFG1H** and **BG1NMe_2_** occupy general positions in the crystal structure and, hence, those compounds do not exhibit any symmetry in the solid state. The B−C (1.547(8)–1.609(2) Å) and C−N (1.379(6)–1.446(16) Å) bond lengths are in the expected range (B−C_ar_: 1.556 Å, C_ar_−N: 1.390 Å)^114^ and comparable to those of other triarylboranes.^37^, ^46^, ^49^, ^115^ Due to steric hindrance, in **BG1Bpin** the B−C bonds to aryl rings with *ortho*‐methyl groups (1.576(3)–1.577(2) Å) are slightly longer than the B−C bonds to aryls without *ortho*‐methyl groups (1.566(4) Å). Similarly, in **BFG1H**, the B−C bonds to aryls with *ortho*‐trifluoromethyl groups (1.602(2)–1.609(2) Å) are longer than the B−C bonds to the inner aryls without methyl groups in the near vicinity (1.554(3)–1.559(2) Å). This is analogous to previously reported *ortho*‐trifluoromethylaryl substituted triarylboranes.^26^, ^104^, ^110^, ^113^, ^116^ In contrast, the NMe_2_ groups on the outer aryl rings of **BG1NMe_2_** have an opposite effect on the B−C bond distances, so that the B−C bonds to the inner aryl rings (1.571(7)–1.578(7) Å) are slightly longer than the B−C bonds to the outer NMe_2_‐bearing aryl rings with *ortho*‐methyl groups (1.547(8)–1.569(7) Å). We previously observed a similar shortening of the respective B−C bond in the presence of a strong electron‐donating NMe_2_ group, which indicates a degree of polarization in the ground state.^46^, ^109^ The boron atoms have a nearly ideal trigonal planar configuration, with the sum of the C−B−C angles being between 359.97(12) and 360.0(9)°. In **BG1NMe_2_**, the sum of the C−N−C angles around the nitrogen atoms varies from 350.4(5) to 360.0(5)°. In all of these compounds, we observe an effect of aryl *ortho*‐methylation on the torsion angles of the aryl moieties with respect to the BC_3_ plane.^37^, ^46^, ^49^, ^115^ Due to steric repulsion, the torsion angles are significantly increased in *ortho*‐methylated aryls (45.06(14)–54.31(14)°) compared to non‐methylated aryls (22.94(19)–44.39(15)°). In **BFG1H**, several B⋅⋅⋅F distances in the range 2.584(2)–2.954(2) Å are observed, which are significantly shorter than the sum of the van der Waals radii for boron and fluorine (3.39 Å).^117^ This was previously observed in boranes with *ortho*‐CF_3_ aryl moieties.^26^, ^104^, ^113^, ^116^ As the respective fluorine atoms are directly above and below the boron center, it is most likely that the lone pair electrons of these fluorine atoms interact with the empty p_z_‐orbital of the boron center, which results in a stabilizing effect of the *ortho*‐CF_3_ groups on the triarylborane. The packing of our molecules in their crystal structures is determined by their bulk. Due to the large torsion angles between the aryl moieties and the BC_3_ planes, there are no π⋅⋅⋅π stacking interactions present between the molecules. The crystal structure of **BG1Bpin** contains strongly disordered ethanol solvent molecules and, hence, there is no obvious direct interaction between the molecules of the main compound themselves. The crystal structure of **BFG1H** mainly exhibits H⋅⋅⋅F, H⋅⋅⋅H, and F⋅⋅⋅F contacts, while in **BG1NMe_2_**, H⋅⋅⋅H contacts dominate, and weak interactions are observed involving a disordered dichloromethane solvent molecule of low‐occupation.

### Electrochemistry

**Figure 3:**
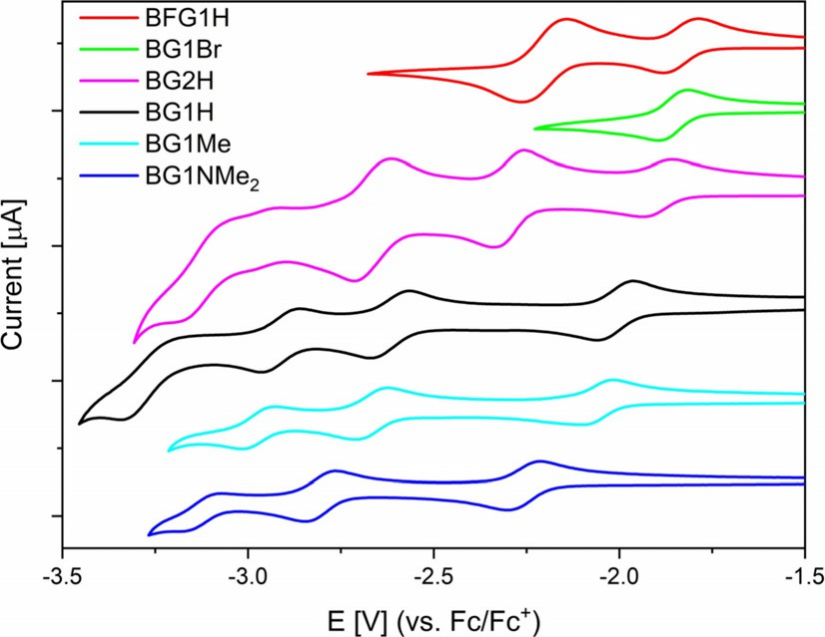
Cyclic voltammograms of BG1H (black), **BG1Me** (cyan), **BG1NMe_2_** (blue), **BG1Br** (green), **BFG1H** (red) and **BG2H** (magenta) in THF ordered by decreasing first reduction potential. All samples are referenced against the Fc/Fc^+^ redox couple.

Cyclic voltammograms of the six borane dendrimers were recorded in THF with [*n*Bu_4_N][PF_6_] as the electrolyte and a scan rate of 250 mVs^−1^ (Figure 3) in order to determine their reduction potentials (Table 2), which are referenced to the ferrocene/ferrocenium redox couple (Fc/Fc^+^).

**Table 2:** Reduction potentials of **BG1H**, **BG1Me**, **BG1NMe_2_**, **BG1Br**, **BFG1H** and **BG2H** ordered by decreasing first reduction potential, referenced vs. the Fc/Fc^+^ redox couple.

E vs. Fc/Fc^+^ [V]				
Compound	E_1/2_ (red 1)	E_1/2_ (red 2)	E_1/2_ (red 3)	E_p.c._ (red 4)
**BFG1H**	−1.83	−2.19	−2.98^[a]^	N.D.
**BG1Br**	−1.89	−2.56 (irr)^[a]^	N.D.	N.D.
**BG2H**	−1.90	−2.30	−2.66	−3.19^[b]^
**BG1H**	−2.01	−2.62	−2.91	−3.34^[b]^
**BG1Me**	−2.05	−2.62	−2.96	N.D.
**BG1NMe_2_**	−2.24	−2.79	−3.11	N.D.

[a] E_p.c._ is given for irreversible reduction potentials. [b] Too close to the solvent window to determine accurately whether or not it is reversible. Multiple potential sweeps of all reductions showed no loss in intensity, indicating reversibility.

Compared to BMes_3_ (E_1/2_=2.8 V (calculated from measurements vs. SCE with Fc/Fc^+^=0.4 V vs. SCE))^118^ all conjugated borane dendrimers exhibit anodically shifted first reduction potentials, showcasing the electron withdrawing nature of the triarylborane moieties.^115^ For the parent compound **BG1H**, three distinct reversible reduction waves were observed. The fourth reduction is too close to the solvent window, and thus, whether or not it is reversible or irreversible cannot be determined accurately. However, almost no decrease in intensity was observed when repeatedly cycling through all four reductions. This indicates that the fourth reduction is also reversible. The difference between the first and second reduction wave is 610 mV and the difference between the 2^nd^ and 3^rd^ is 290 mV. According to Kaim and Schulz, the difference between equivalent redox centers can be used as an indicator for the conjugation of the system as, in a well conjugated system, the reduction potentials are dependent on each other.^118^, ^119^, ^120^ They observed a peak splitting of 690 mV for 1,4‐bis(dimesitylboryl)benzene and 250 mV for 4,4’‐bis(dimesitylboryl)‐1,1’‐biphenyl.^119^ Similarly, we previously observed a peak splitting of 280 mV for 2,7‐bis(dimesitylborane)pyrene^121^ and 380 mV for 2,5,8,11‐tetrakis(dimesitylborane)perylene.^32^ It is important to note that, in our systems, not all boron centers are equivalent. The central boron atom can be expected to exhibit a lower reduction potential compared to that of the boron centers of the outer periphery. Assuming that the 1^st^ reduction event corresponds to the central boron center, and the consecutive reductions correspond to the outer periphery, the peak splitting between the 2^nd^ and 3^rd^ reductions should provide a more accurate description of the conjugation. The peak splitting for **BG1Me** (Δ*E*(1^st^/2^nd^)=570 mV; Δ*E*(2^nd^/3^rd^)=340 mV) and **BG1NMe_2_** (Δ*E*(1^st^/2^nd^)=550 mV; Δ*E*(2^nd^/3^rd^)=320 mV) is similar to that of **BG1H**. However, for **BFG1H** (Δ*E*(1^st^/2^nd^)=360 mV), due to the electron withdrawing ^F^Xyl moieties, it can be assumed that the first and second reductions correspond to the peripheral boron centers and, as such, the separation can indeed be regarded as a measure of the communication between the boron centers. Functionalization at the *para*‐position of the outer aryl layer has a large influence on the reduction potentials. Electron donating substituents (Me and NMe_2_) shift the reduction potential cathodically, while electron withdrawing substituents (Br) shift the reduction potential anodically. Three reversible reduction events were observed for both **BG1Me** and **BG1NMe_2_**. The fourth reduction is shifted beyond the limit of the solvent. In contrast, **BG1Br** exhibits only one reversible reduction. At more negative potentials, only irreversible events occur (Figure S50). The reason for this is not clear, but one possibility would be activation of the C‐Br bonds. The electron withdrawing ^F^Xyl groups in **BFG1H** also shift the reduction potential anodically. Analogously to **BG1Br**, only irreversible reductions occur at more negative potentials (Figure S51). Interestingly, the first reduction potential of **BFG1H** is only slightly anodically shifted compared to that of **BG1Br** (Δ*E*_1/2_=60 mV). This illustrates the importance of the position of the electron withdrawing groups. The effect of the substitution pattern of trifluoromethyl groups on the reduction potential of triarylboranes was previously studied by Wildgoose and co‐workers.^122^ The second reduction wave of **BFG1H** exhibits much higher intensity compared to the first one (integration of 1:2). This is most likely due to two coincidental one electron reductions, indicating a smaller conjugation of the system compared to that in the non‐trifluoromethylated derivatives. This is also observed for the reduction waves of **BG2H**. The first and 4^th^ reductions exhibit a much lower intensity than the 2^nd^ and 3^rd^ reductions (integration of 1:2:3). Assuming that the 2^nd^ and 3^rd^ reduction waves correspond to two and three coincidental one electron reductions, respectively, it should be possible to reduce **BG2H** reversibly seven times. This is a potentially interesting property for accumulators.^123^

### Photophysical properties

**Figure 4:**
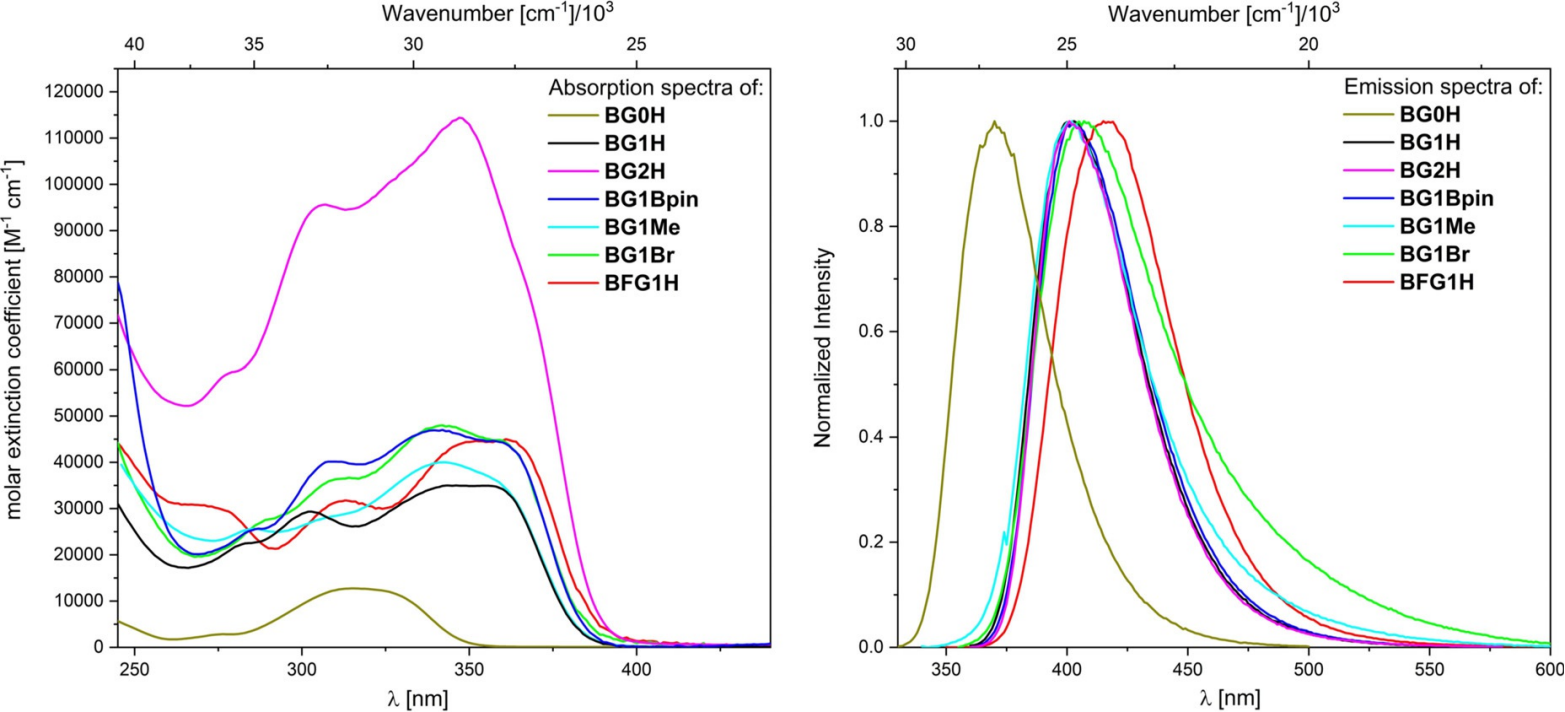
Absorption (left) and emission (right) spectra of **BG0H** (gold), **BG1H** (black), **BG2H** (magenta) **BG1Bpin** (blue), **BG1Br** (green), **BFG1H** (red), and **BG1Me** (cyan).

**Figure 5:**
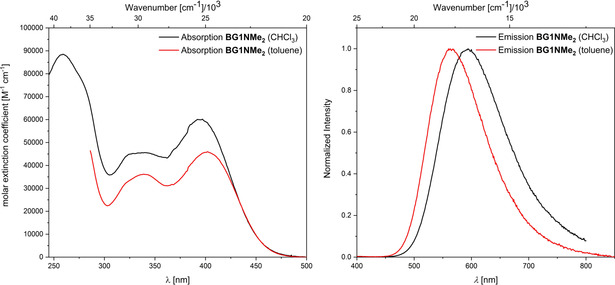
Absorption (left) and emission (right) spectra of **BG1NMe_2_** in CHCl_3_ (black) and toluene (red).

The photophysical data of conjugated dendrimers **BG1H**, **BG1Bpin**, **BG1Me**, **BG1NMe_2_**, **BG1Br**, **BFG1H**, and **BG2H** were determined in CHCl_3_ due to the high solubility of all compounds in that solvent (Figure 4 and Figure 5 and Table 3). The photophysical properties of **BG1H** and **BG1NMe_2_** were also recorded in THF or toluene, respectively, in order to observe possible solvent polarity dependence. To compare the properties of the dendrimers accurately with those of the core moiety **BG0H**, photophysical properties of **BG0H** were also investigated.

**Table 3:** Photophysical properties of conjugated borane dendrimers **BG1H**, **BG1Bpin**, **BG1Me**, **BG1NMe_2_**, **BG1Br**, **BFG1H**, and **BG2H**, and the core moiety **BG0H**.

Compound	Solvent	λ_max_ (abs.) [nm]	*ϵ* 10^3^ [M^−1^ cm^−1^]; (log *ϵ*)	λ_max_ (em.) [nm]	Apparent Stokes Shift [cm^−1^]	τ_f_ [ns]	τ_0_ [ns]^[a]^	Φ_f_	k_r_ [10^9^ s^−1^]^[c]^	k_nr_ [10^9^ s^−1^]^[b]^
**BG0H**	CHCl_3_	315	13 (4.11)	370	4700	1.6	22.9	0.07	0.04	0.6
**BG1H**	CHCl_3_	346	35 (4.54)	403	4100	2.5	14.7	0.17	0.07	0.3
	THF	344	35 (4.54)	403	4300	2.7	15.0	0.18	0.07	0.3
**BG1Bpin**	CHCl_3_	342	47 (4.67)	403	4400	2.4	11.4	0.21	0.09	0.3
**BG1Me**	CHCl_3_	343	40 (4.60)	402	4300	2.0	22.2	0.09	0.05	0.5
**BG1NMe_2_**	toluene	402	46 (4.66)	560	7000	^[d]^	^[d]^	^[d]^	^[d]^	^[d]^
	CHCl_3_	390	60 (4.78)	593	8800	^[d]^	^[d]^	^[d]^	^[d]^	^[d]^
**BG1Br**	CHCl_3_	340	48 (4.68)	407	4800	1.0^[e]^	16.7	0.06	0.06	0.9
**BFG1H**	CHCl_3_	361	45 (4.65)	416	3700	4.6	35.4	0.13	0.03	0.2
**BG2H**	CHCl_3_	347	114 (5.06)	401	3900	2.1	21.0	0.10	0.05	0.4

[a] Calculated from τ_f_/Φ_fl_; [b] The non‐radiative rate constants were calculated from k_nr_=(1−Φ_fl_)/τ_f_; [c] The radiative rate constants were calculated from k_r_=Φ_fl_/τ_f_; [d] not photostable; [e] calculated average of three lifetimes: 0.7 (76 %), 1.8 (18 %), 9.8 (6 %).

Compared to the core moiety **BG0H** (λ_abs, max_=315 nm, λ_em, max_=370 nm), all dendrimers exhibit a bathochromically shifted lowest energy absorption and emission (**BG1H**, **BG1Bpin**, **BG1Me**, **BG1Br** and **BG2H**: λ_abs, max_ ≈345 nm, λ_em, max_ ≈405 nm; **BFG1H**: λ_abs, max_=361 nm, λ_em, max_=416 nm; **BG1NMe_2_**: λ_abs, max_=390 nm, λ_em, max_=593 nm). This shows that the conjugated system has been enlarged as compared to the core moiety; however, the electronic parameters of the different substituents also need to be considered. The observed absorption and emission spectra of **BG1H**, **BG1Bpin**, **BG1Me**, **BG1Br**, and even **BG2H**, are very similar. Thus, in contrast to what was observed for the electrochemical properties, expanding or functionalizing the 1^st^ generation dendrimer at the *para* position of the outer layer, with electron withdrawing substituents or weakly donating substituents, influences the energy of the absorption and emission only slightly. This also indicates that the nature of excitations and emissions are very similar. In analogous systems, such as those of Tamao, Yamaguchi, and co‐workers,^15^ the transitions were classified as π–π* transitions. This is also likely the case for our dendrimers. However, it can be assumed that there is also a large π‐p_z_ contribution due to the empty p_z_‐orbital localized on the boron centers. In addition to the bathochromic shift, extension of the systems from the core moiety **BG0H** to **BG1H**, increases the molar extinction coefficient (*ϵ*(**BG0H)**=13×10^3^ [M^−1^ cm^−1^], *ϵ*(**BG1H)**=35×10^3^ [M^−1^ cm^−1^]). Functionalization at the *para*‐position of the outer layer slightly increases the molar extinction coefficient (*ϵ*(**BG1Bpin**)=47×10^3^ [M^−1^ cm^−1^], *ϵ*(**BG1Me**)=40×10^3^ [M^−1^ cm^−1^], *ϵ*(**BG1Br**)=48×10^3^ [M^−1^ cm^−1^]), while expanding the system by one more generation greatly increases the molar extinction coefficient (**BG2H**=114×10^3^ [M^−1^ cm^−1^]). Comparison of the extinction coefficients of **BG0H**, **BG1H**, and **BG2H**, shows an almost linear increase with the number of triarylborane subunits. Combined with the fact that no further red‐shift is observed from **BG1H** to **BG2H**, this indicates that the maximum conjugation is reached at the first generation for these systems. Thus, the 2^nd^ generation dendrimer **BG2H** consists of 2.5 localized, conjugated 1^st^ generation subunits that only weakly interact with each other. This phenomenon is of potential application for a photonic antenna.^76^ Exchanging the xylyl moieties at the outer periphery with ^F^Xyl groups slightly red shifts the absorption and emission and increases the molar extinction coefficient slightly compared to **BG1H** (*ϵ*(**BFG1H)**=45×10^3^ [M^−1^ cm^−1^]). This small bathochromic shift is due to the electron‐withdrawing nature of the ^F^Xyl moiety. It is surprising that no analogous bathochromic shift was observed for **BG1Br**, as the electrochemical measurements indicate a very similar electron withdrawing effect for **BFG1H**. Adding a *para*‐NMe_2_ group, which is a strong *π*‐donor, drastically shifts both absorption and emission bathochromically, and increases the molar extinction coefficient of the lowest energy absorption compared to that of **BG1H** (*ϵ*(**BG1NMe_2_**, CHCl_3_)=60×10^3^ [M^−1^ cm^−1^]). Furthermore, a bathochromic shift of the emission with increasing solvent polarity was observed for **BG1NMe_2_** ((λ_em,max_(toluene) = 560 nm; λ_em,max_(CHCl_3_) = 593 nm), but it was fairly small. This indicates that the nature of the transition is significantly changed by the introduction of a strong *π*‐donor, most likely from a local excitation (LE) to an intramolecular charge transfer (ICT). The small bathochromic shift of about 1000 cm^−1^ can be attributed to the radial structure of the dendrimers. As such, the change in dipole moment and the subsequent dependence of the emission on solvent polarity can only occur due to symmetry breaking.^27^, ^47^, ^124^ All compounds, except **BG1Br**, exhibit mono‐exponential radiative decays of their excited states. Fluorescent lifetimes τ_f_ of all of the conjugated borane dendrimers are ca. 2 ns, with **BG1Br** exhibiting a shorter lifetime τ_f_=1 ns (average over three lifetimes) and **BFG1H** exhibiting a slightly longer lifetime τ_f_=4.6 ns. We did not measure the lifetime of **BG1NMe_2_** as partial photodecomposition was observed during the measurements (faster in CHCl_3_ than in toluene). The quantum yields of all compounds are modest (Φ_f_≈0.1), which can be attributed to their fast, non‐radiative decay rates. This is likely due to the large number of degrees of freedom the systems have because of their size and flexibility.

### DFT and TD‐DFT studies

**Figure 6:**
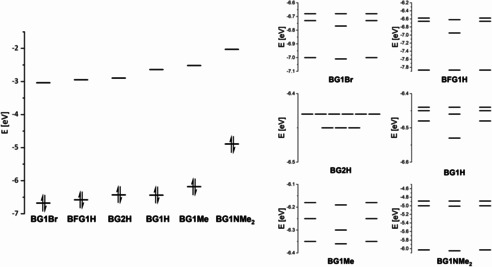
Calculated HOMO and LUMO energies of the conjugated borane dendrimers **BG1Br**, **BFG1 H**, BG2 **H**, **BG1H**, **BG1Me**, and **BG1NMe_2_** (left) and energy diagrams depicting HOMO to HOMO‐8 (right), calculated at the B3LYP/6–31+G(d) level of theory.

DFT and TD‐DFT calculations were carried out to gain further insight into the electronic and photophysical properties of the conjugated borane dendrimers. Due to the large size of the systems, and their apparent high symmetry observed in solution by NMR spectroscopy, all dendrimers were calculated with C_3_ symmetry (**BG1H**, **BG1Me**, **BG1Br**, and **BFG1H)** or D_3_ symmetry (**BG2H)**, in order to reduce the cost of the calculations. The HOMO and LUMO energies are plotted in Figure 6 and listed in Table 4.

**Table 4:** Calculated and experimentally determined HOMO and LUMO energies of conjugated borane dendrimers **BG1Br**, **BFG1H**, **BG2H**, **BG1H**, **BG1Me**, and **BG1NMe_2_**, calculated at the B3LYP/6‐31+G(d) level of theory.

	**BG1Br**		**BFG1H**		**BG2H**		**BG1H**		**BG1Me**		**BG1NMe_2_**	
	calc.	exp.^[a]^	calc.	exp.^[a]^	calc.	exp.^[a]^	calc.	exp.^[a]^	calc.	exp.^[a]^	calc.	exp.^[a]^
LUMO	−3.04	−3.27	−2.95	−3.33	−2.90	−3.26	−2.64	−3.15	−2.52	−3.11	−2.03	−2.92
HOMO	−6.68	−6.49	−6.58	−6.41	−6.43	−6.40	−6.44	−6.36	−6.18	−6.32	−4.89	−5.50

[a] Determined from the half wave potentials: LUMO=−(5.16+E_1/2, red_) eV;^125^, ^126^, ^127^ HOMO=E(HOMO)+E(onset Abs.).

**Figure 7:**
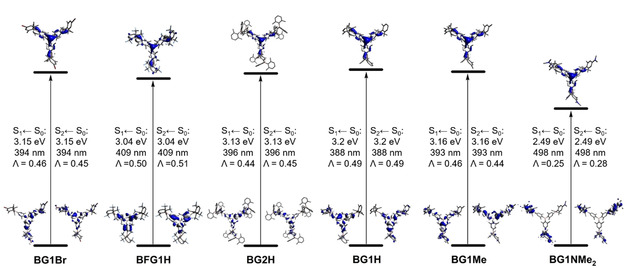
NTOs of the S_1_←S_0_ and S_2_←S_0_ transitions of the conjugated borane dendrimers **BG1Br**, **BFG1H**, and **BG2H**, **BG1H**, **BG1Me**, and **BG1NMe_2_**.

At the B3LYP/6‐31+G(d) level of theory, the calculated LUMO energy levels rise from **BG1Br**<**BFG1H**<**BG2H**<**BG1H**<**BG1Me**<**BG1NMe_2_**. The calculated HOMO energies show almost the same trend, except that the order of **BG1H** and **BG2H** is switched. The experimental HOMO and LUMO energies also show the same trend; however, the order of **BG1Br** and **BFG1H** is switched for the LUMO energies. The calculations describe the trends in HOMO and LUMO energies of the conjugated borane dendrimers rather well, accounting for the fact that all calculations were carried out using symmetry. TD‐DFT calculations were carried out in order to gain more insight into the photophysical properties of the dendrimers. The lowest energy transitions S_1_←S_0_ and S_2_←S_0_ are degenerate for all dendrimers, due to the symmetry used in the calculations. The natural transition orbitals (NTOs) for the S_1_←S_0_ and S_2_←S_0_ transitions of all dendrimers are depicted in Figure 7.

For **BG1Br**, **BFG1H**, **BG2H**, **BG1H**, and **BG1Me**, the S_1_←S_0_ and S_2_←S_0_ transitions correspond to π(xylene)‐p_z_(boron) transitions. The outer xylene moieties have a smaller contribution than the inner xylene moieties. In all cases, the virtual NTOs correspond to the LUMO of the respective dendrimer, which are mainly localized on the four boron centers of the core and the 1^st^ generation. This is also the case for **BG2H**, illustrating the limited conjugation of the 2^nd^ generation dendrimer, and it explains the similarity of its absorption and emission spectra to those of the 1^st^ generation dendrimers. For **BG1NMe_2_**, the S_1_←S_0_ and S_2_←S_0_ transitions can be classified as charge transfer (CT) transitions from the 4‐dimethylaminoxylene moieties of the outer layer to the four boron centers. This is also supported by the small orbital overlap coefficients (0≤*Λ*≤1, where *Λ*=0 corresponds to no overlap and *Λ*=1 corresponds to complete overlap),^128^ that are significantly lower (*Λ*=0.25 and 0.28) than those of the other dendrimers (*Λ*≈0.5). The specific orbitals contributing to the transitions vary, depending on the substitution patterns of the outer periphery of the dendrimers.

## Conclusions

We have designed and applied a new divergent strategy for the synthesis of conjugated borane dendrimers **BG1Br**, **BG1H**, **BG1Me**, **BG1NMe_2_**, and **BG2H**. The three step divergent approach, consisting of: 1) functionalization, via iridium‐catalyzed C−H borylation; 2) activation, via fluorination of the boronate ester generated with K[HF_2_] or [N(*n*Bu)_4_][HF_2_]; 3) expansion, via reaction of the trifluoroborate salts with an aryl Grignard reagent, works as designed up until the 1^st^ generation. After the 1^st^ generation, solubility issues forced us to modify the synthesis slightly. The 2^nd^ generation dendrimer **BG2H** was synthesized after exchanging the cation of the fluorination step from potassium to tetra‐*n‐*butylammonium, in order to improve the solubility in organic solvents. Substrate tolerance is mainly limited by the reactivity of the aryl Grignard reagent. Additionally, we synthesized **BFG1H** by a convergent approach. All dendrimers were investigated via cyclic voltammetry, UV/Vis absorption and emission spectroscopy, and by DFT and TD‐DFT calculations, in order to gain more insight into their electrochemical and photophysical properties. All dendrimers, except **BG1Br**, exhibit multiple, distinct and reversible reductions, that can be easily modified via the substitution pattern of the outer periphery of the dendrimers. As such, they might have potential application as molecular accumulators.^123^ In contrast to the electrochemical properties, the photophysical properties of the dendrimers are not strongly influenced by the substitution pattern of the outer periphery. Only strong donor moieties significantly altered the nature of the excitation and emission of the dendrimers. While conjugation within the systems does not increase after the 1^st^ generation, the molar extinction coefficient does, which is of possible interest for the design of a photonic antenna.^76^

## Conflict of interest

The authors declare no conflict of interest.

## Supporting information

As a service to our authors and readers, this journal provides supporting information supplied by the authors. Such materials are peer reviewed and may be re‐organized for online delivery, but are not copy‐edited or typeset. Technical support issues arising from supporting information (other than missing files) should be addressed to the authors.

Supplementary
