# Supplementary material for: An Iterative Divergent Approach to Conjugated Starburst Borane Dendrimers
Source: Chemistry. 2020 Sep 16;26(57):12951–63. doi: 10.1002/chem.202001985 (PMC7590090; doi:10.1002/chem.202001985)
Supplement: Supplementary file 1 — Supplementary [file CHEM-26-12951-s001.pdf]

Chemistry–A European Journal

Supporting Information

An Iterative Divergent Approach to Conjugated Starburst Borane Dendrimers

Florian Rauch,[a] Peter Endres,[a] Alexandra Friedrich,[a] Daniel Sieh,[a] Martin Hähnel,[a]
Ivo Krummenacher,[a] Holger Braunschweig,[a] Maik Finze,[a] Lei Ji,[a, b] and Todd B. Marder*[a]

Supporting Information

Table of contents

General experimental procedures 2

Synthesis 5

Tris(2,6-dimethyl-4-(4,4,5,5-tetramethyl-1,3,2-dioxaborolan-2-yl)phenyl)borane (BG0Bpin) 5

Tris(2,6-dimethyl-4-(trifluoroborate)phenyl)borane, tripotassium salt (BG0BF$_3$K) 6

Tris(4-(bis(2,6-dimethylphenyl)boranyl)-2,6-dimethylphenyl)borane (BG1H) 6

Tris(4-(bis(2,6-dimethyl-4-(4,4,5,5-tetramethyl-1,3,2-dioxaborolan-2-yl)phenyl)boranyl)-2,6-dimethylphenyl)borane (BG1Bpin) 7

Tris(4-(dimesitylboranyl)-2,6-dimethylphenyl)borane (BG1Me) 7

Tris(4-(bis(4-bromo-2,6-dimethylphenyl)boranyl)-2,6-dimethylphenyl)-borane (BG1Br) 8

4,4',4'',4''',4'''',4'''''-((Boranetriyltris(3,5-dimethylbenzene-4,1-diyl))tris-(boranetriyl))hexakis(N,N,3,5-tetramethylaniline) (BG1NMe$_2$) 9

Tris(4-(bis(2,6-bis(trifluoromethyl)phenyl)boranyl)-2,6-dimethylphenyl)-borane (BFG1H) 10

Tris(4-(bis(2,6-dimethyl-4-(trifluoroborate)phenyl)boraneyl)-2,6-dimethylphenyl)borane, hexa(tetrabutylammonium) salt (BG1BF$_3$N(*n*Bu)$_4$) 11

Tris(4-(bis(4-(bis(2,6-dimethylphenyl)boraneyl)-2,6-dimethylphenyl)boraneyl)-2,6-dimethylphenyl)borane (BG2H) 12

NMR Spectra 13

Crystal structure determination 32

Photophysical data 36

Electrochemistry 38

TD-DFT Calculations 39

Theoretical calculations: Cartesian coordinates 56

References 69

General experimental procedures

Unless otherwise noted, the following conditions apply.

All syntheses were carried out using standard Schlenk and glovebox techniques under an argon atmosphere. The solvents used were dried using a solvent purification system (SPS) from Innovative Technology and were degassed and stored over molecular sieves under argon. Deuterated solvents (CD_2Cl_2, $CDCl_3$, C_6D_6, acetone-*d*$_6$, and DMSO-*d*$_6$) used for NMR spectroscopy were purchased from Cambridge Isotope Laboratories. C_6D_6 was dried over molecular sieves and stored under an argon atmosphere before use. Boron trifluoride diethyl etherate was purchased from Merk Millipore. *n*-BuLi (2.5 M solution in hexane), methyllithium (1.6 M solution in Et_2O) and *tert*-butyllithium (1.7 M solution in pentane) were purchased from Acros Organics and used as received. The compounds 2-bromo-1,3-dimethylbenzene and 1,3-bis(trifluoromethyl)benzene were purchased from ABCR. B_2pin_2 was kindly provided by AllyChem Co. Ltd. (Dalian, China). $[Ir(COD)(OMe)]_2$ was synthesized by our group according to a literature procedure from 1985,[1] 4-bromo-N,N,3,5-tetramethylaniline[2] and [N(*n*Bu$_4$)][HF$_2$][3] were synthesized according to literature procedures and bis(2,6-bis(trifluoromethyl)phenyl)(4-iodo-3,5-dimethylphenyl)borane (**BFD1H**) was synthesized according to our previously published procedure.[4] Mesitylmagnesiumbromide and (2,6-dimethylphenyl)magnesiumbromide were synthesized according to literature procedures and their concentrations were determined via titration with iodine,[5, 6] and tris(2,6-dimethylphenyl)borane[7] was synthesized according to a literature procedure.

Solution NMR spectra were recorded on a Bruker Avance 200 (operating at ^{1}H: 199.9 MHz, ^{11}B{^{1}H}: 64.1 MHz, ^{19}F{^{1}H}: 188.1 MHz) or a Bruker Avance 500 FT NMR spectrometer (operating at ^{1}H: 500 MHz, ^{11}B{^{1}H}: 160 MHz, ^{13}C{^{1}H}: 126 MHz, ^{19}F{^{1}H}: 470.6 MHz. Chemical shifts (δ) are given in ppm and are referenced to external $BF_3 \cdot Et_2O$ (^{11}B{^{1}H}) and $CFCl_3$ (^{19}F{^{1}H}). ^{1}H NMR spectra were referenced via residual proton resonances of $CDCl_3$ (7.26 ppm), CD_2Cl_2 (5.32 ppm), acetone-*d*$_6$ (2.05 ppm), and C_6D_6 (7.16 ppm).[8] ^{13}C{^{1}H} spectra were referenced to $CDCl_3$ (77.16 ppm), CD_2Cl_2 (53.84 ppm), acetone-*d*$_6$ (29.84 ppm) and C_6D_6 (128.06 ppm).[8]

Solid-state magic angle spinning (MAS) NMR spectra were recorded using a Bruker DSX-400 solid state spectrometer (^{11}B: 128.3 MHz, ^{13}C: 100.6 MHz, ^{19}F: 376.5 MHz, rotor 4 mm OD).

GCMS analyses were performed on an Agilent Technologies GCMS system (GC 7890A, EI-MS 5975C). HRMS were recorded using a Thermo Scientific Exactive Plus Orbitrap MS system with either an Atmospheric Sample Analysis Probe (ASAP) or by Electro-Spray Ionization (ESI).

Column chromatography was performed with silica gel 60 (40-63 μ) (purchased from VWR), or alumina 90 active basic (purchased from Merck), and automated flash chromatography was performed on silica gel (Biotage SNAP catridge KP-Sil 10 g or KP-Sil

100 g), obtained from Biotage, using a Biotage® Isolera Four Flash system. Solvents were generally removed using a rotary evaporator *in vacuo* at a maximum temperature of 55 °C.

Single-crystal X-ray diffraction: Crystals suitable for single-crystal X-ray diffraction were selected, coated in perfluoropolyether oil, and mounted on MiTeGen sample holders. Diffraction data were collected on Bruker X8 Apex II 4-circle diffractometers with CCD area detectors using Mo-K_α radiation monochromated by graphite (**BFG1H**) or multi-layer focusing mirrors (**BG1BPin** and **BG1NMe₂**). The crystals were cooled using an open flow N_2 Oxford Cryostream or Bruker Cryoflex II low-temperature device. Data were collected at 100 K. The images were processed and corrected for Lorentz-polarization effects and absorption as implemented in the Bruker software packages. The structures were solved using the intrinsic phasing method (SHELXT)[9] and Fourier expansion technique. All non-hydrogen atoms were refined in anisotropic approximation, with hydrogen atoms 'riding' in idealized positions, by full-matrix least squares against F^2 of all data, using SHELXL[10] software and the SHELXLE graphical user interface.[11] The unit cell of **BG1BPin** contains six disordered ethanol solvent molecules, which have been treated as a diffuse contribution to the overall scattering without specific atom positions by SQUEEZE/PLATON.[12] In **BG1NMe$_2$**, one of the voids of the crystal structure is fully occupied by a dichloromethane solvent molecule, while another void is nearly empty being only partly occupied by disordered dichloromethane molecules. Fractions of 0.085 and 0.069 were refined for these disordered parts resulting in an overall fraction of only 0.154 molecules in this void. Diamond[13] software was used for graphical representation. Other structural information was extracted using OLEX2[14] software. Crystal data and experimental details are listed in Table S1; full structural information has been deposited with Cambridge Crystallographic Data Centre. CCDC-1990178 (**BG1Bpin**), 1990179 (**BG1NMe$_2$**), and 1990180 (**BFG1H**).

Photophysical measurements: All measurements were performed in standard quartz cuvettes (1 cm x 1 cm cross-section). UV–visible absorption spectra were recorded using an Agilent 8453 diode array UV-visible spectrophotometer. The extinction coefficients of **BG1H** and **BG1Bpin** were calculated from 3 independently prepared samples in THF and chloroform, respectively.

Emission spectra were recorded using an Edinburgh Instruments FLSP920 spectrometer equipped with a double monochromator for both excitation and emission, operating in right-angle geometry mode, and all spectra were fully corrected for the spectral response of the instrument. All solutions used for photophysical measurements had a concentration lower than 2×10^{-5} M to minimize inner filter effects during fluorescence measurements.

Fluorescence quantum yields of solutions were measured using a calibrated integrating sphere (inner diameter: 150 mm) from Edinburgh Instruments combined with the FLSP920 spectrometer described above. For solution-state measurements, the longest-wavelength absorption maximum of the compound in the respective solvent was chosen as the excitation wavelength.

Fluorescence lifetimes were recorded using the time-correlated single-photon counting (TCSPC) method using the same FLSP920 spectrometer described above. Solutions were

excited with a picosecond pulsed diode laser at wavelengths 320 nm (for **BG1H** and **BG1Bpin)** and 376 nm (for **BG1Me**, **BG1Br**, **BG1CF$_3$H** and **BG1NMe$_2$**). The full width at half maximum (FWHM) of the laser pulses were ca. 50–100 ps, while the instrument response function (IRF) had a FWHM of ca. 1.0 ns, measured from the scatter of pure solvent at the excitation wavelength. Decays were recorded to at least 10000 counts in the peak channel with a record length of at least 4800 channels. The band pass of the monochromator was adjusted to give a signal count rate of <10 kHz. Iterative reconvolution of the IRF with one decay function and non-linear least-squares analysis were used to analyze the data. The quality of the fit was judged by the calculated value of the reduced χ^2 and visual inspection of the weighted residuals.

Electrochemical measurements: All cyclic voltammetry experiments were conducted in an argon-filled glovebox using a Gamry Instruments Reference 600 potentiostat. A standard three-electrode cell configuration was employed using a platinum disk working electrode, a platinum wire counter electrode, and a silver wire reference electrode separated by a Vycor frit, serving as the reference electrode. The redox potentials are referenced to the ferrocene/ferrocenium ([Fc/Fc$^+$]) redox couple by using decamethylferrocene ([Cp*$_2$Fe]; $E_{1/2}$ = –0.532 V in CH_2Cl_2) as an internal standard. Tetra-*n*-butylammonium hexafluorophosphate ([*n*Bu$_4$N][PF$_6$]) was employed as the supporting electrolyte. Compensation for resistive losses (*iR* drop) was employed for all measurements.

Theoretical Studies: All calculations (DFT and TD-DFT) were carried out with the Gaussian 09 (9.E.01)[15] program package and were performed on a parallel cluster system. GaussView (6.0.16), Avogadro (1.2.0)[16] and multiwfn[17] were used to visualize the results, to measure calculated structural parameters, and to plot orbital surfaces (isovalue: ± 0.030 [e a_0^{-3}]$^{1/2}$). The ground-state geometries were optimized using the B3LYP functional[18] in combination with the 6-31G basis set.[19, 20] The ultrafine integration grid and symmetry constraints were used for all molecules. Frequency calculations were performed on the optimized structures to confirm them to be local minima showing no negative (imaginary) frequencies. Based on these optimized structures, the lowest-energy vertical transitions (gas-phase and solvent correction using the polarizable continuum model) were calculated (singlets, at least 12 states) by TD-DFT, using the B3LYP functional in combination with the 6-31G+(d) basis set.

Synthesis

Tris(2,6-dimethyl-4-(4,4,5,5-tetramethyl-1,3,2-dioxaborolan-2-yl)phenyl)borane (BG0Bpin)

Tris(2,6-dimethylphenyl)borane (4.0 g, 12.3 mmol), B_2pin_2 (13.7 g, 49.0 mmol), $[Ir(COD)(OMe)]_2$ (243.8 mg, 0.37 mmol) and dtbpy (197 mg, 0.74 mmol) were dissolved in THF (120 mL) and stirred at 70 °C for 3 d. The solvent was removed under reduced pressure and the residue dissolved in a hexane/CH_2Cl_2 mixture (1:1, 150 mL) and filtered through a plug (silica: hexane/CH_2Cl_2). The solvent was removed under reduced pressure and the remaining residue washed with methanol. Tris(2,6-dimethyl-4-(4,4,5,5-tetramethyl-1,3,2-dioxaborolan-2-yl)phenyl)borane was isolated as a white solid in 87% yield (7.58 g, 10.7 mmol).

^{1}H NMR (300 MHz, $CDCl_3$): δ (ppm) = 7.36 (s, 6H), 2.02 (s, 18H), 1.37 (s, 36H).

^{11}B NMR (128 MHz, $CDCl_3$): δ (ppm) = 84.0 (br), 31.5 (br).

^{13}C{^{1}H} NMR (75 MHz, $CDCl_3$): δ (ppm) = 149.8 (C_q, 3C), 139.7(C_q, 6C), 133.9 (CH, 6C), 129.9 (C_q, 3C), 83.9 (C_q, 6C), 25.1 (CH_3, 6C), 25.0 (CH_3, 6C), 22.9 (CH_3, 6C).

HRMS (APCI neg): calc.: 704.4768 [m/z]; found: 704.4778 [M^-]

Elemental analysis calcd. [%] for $C_{42}H_{60}B_4$: C 71.64, H 8.59; found: C 71.20 H 8.69.

Tris(2,6-dimethyl-4-(trifluoroborate)phenyl)borane, tripotassium salt (BG0BF$_3$K)

To a solution of tris(2,6-dimethyl-4-(4,4,5,5-tetramethyl-1,3,2-dioxaborolan-2-yl)phenyl)borane (2.18 g, 3.1 mmol) in THF (60 mL), a solution of K[HF$_2$] (2.90 g, 37.1 mmol) in water (7 mL) was added and stirred for 1 h. The precipitate was isolated by filtration and washed with THF (4 x 10 mL) and an acetone/water mixture (1:9, 50 mL), successively. Tris(2,6-dimethyl-4-(trifluoroboranyl)phenyl)borane, tripotassium salt was obtained as a white solid in 91% yield (1.81 g).

^{1}H NMR (200 MHz, 298 K, DMSO-d$_6$): δ (ppm) = 6.82 (s, 6H), 1.86 (s, 12H).

^{11}B NMR (95 MHz, 298 K, DMSO-d$_6$): δ (ppm) = 3.3 (s, br).

^{11}B NMR (128 MHz, 298 K, D$_2$O): δ (ppm) = 82.3 (br), 3.9 (br).

^{19}F NMR (188 MHz, 298 K, DMSO-d$_6$): δ (ppm) = –138.9 (br).

^{13}C{^{1}H} NMR (101 MHz, 298 K, D$_2$O): δ (ppm) = 146.5 (C$_q$, 3C), 139.9 (C$_q$, 6C), 130.0 (CH, 6C), 22.0 (CH$_3$, 6C).

^{13}C-SS NMR (249 MHz, 298 K): δ (ppm) = 146.4, 139.5, 130.8, 22.6.

^{11}B-SS NMR (128 MHz, 298 K): δ (ppm) = 78.5 (η_Q = 0.1), 4.9 (η_Q = 0.35).

^{19}F-SS NMR (377 MHz, 298 K): δ (ppm) = –141.1.

HRMS (ESI neg): calc.: 175.7374 [m/z]; found: 175.7365 [M^{3-} - 3 K^+]

Tris(4-(bis(2,6-dimethylphenyl)boranyl)-2,6-dimethylphenyl)borane (BG1H)

To a suspension of tris(2,6-dimethyl-4-(trifluoroborate)phenyl)borane, tripotassium salt (1.0 g, 1.6 mmol) in THF (50 mL), a solution of (2,6-dimethylphenyl)magnesium bromide in THF (13 mL, 1.1 M, 10.2 mmol) was added. The reaction was stirred for 18 h at ambient temperature. The excess Grignard reagent was quenched with water (10 mL), then the solvent was removed under reduced pressure. The remaining solid was dissolved in refluxing chloroform precipitated with cold ethanol. **BG1H** was obtained by filtration as a white solid in 31% yield (504 mg, 0.5 mmol).

^{1}H NMR (500 MHz, 298 K, CDCl$_3$) δ (ppm) = 7.17 (t, J = 8 Hz, 6H), 7.05 (s, 6H), 6.97 (d, J = 8 Hz, 12H), 2.04 (s, 36H), 1.99 (s, 18H).

^{11}B-SS NMR (128 MHz) , 298 K δ (ppm) = 78.2 (η_Q = 0.0), 75.0 (η_Q = 0.0).

^{11}B NMR (160 Hz, 298 K, CDCl$_3$): δ (ppm) = 83.4 (br).

^{13}C{^{1}H} NMR (126 MHz, 298 K, CDCl$_3$): δ (ppm) = 150.6 (C$_q$, 3C), 147.7 (C$_q$, 3C), 144.9 (C$_q$, 6C), 140.8 (C$_q$, 6C), 139.9 (C$_q$, 6C), 135.2 (CH, 6C), 129.0 (CH, 6C), 127.4 (CH, 12C), 23.7 (CH$_3$, 6C), 23.1 (CH$_3$, 12C).

MALDI-TOF m/z = 986.367 [M^-]

Elemental analysis calcd. [%] for $C_{72}H_{78}B_4$: C 87.65, H 7.97; found: C 87.15 H 8.11.

Tris(4-(bis(2,6-dimethyl-4-(4,4,5,5-tetramethyl-1,3,2-dioxaborolan-2-yl)phenyl)boranyl)-2,6-dimethylphenyl)borane (BG1Bpin)

BG1H (300 mg, 0.3 mmol), B_2pin_2 (926 mg, 3.7 mmol), $[Ir(COD)(OMe)]_2$ (36 mg, 0.4 mmol) and dtbpy (30 mg, 0.7 mmol) were suspended in THF (50 mL) and stirred at 70 °C for 2 d. The reaction was filtrated and the remaining solid was washed with hexane (2 x 10 mL) and MeOH (2 x 10 mL), then dissolved in hot chloroform and precipitated with cold ethanol. **BG1Bpin** was isolated by filtration as a white solid in 76% yield (400 mg, 0.23 mmol).

^{1}H NMR (500 MHz, 298 K, $CDCl_3$): δ (ppm) = 7.41 (s, 12H), 6.99 (s, 6H), 2.04 (s, 36H), 1.93 (s, 18H), 1.35 (s, 74H).

^{11}B-SS NMR (128.3 MHz, 298 K): δ (ppm) = 80.3 (η_Q = 0.0), 74.4 (η_Q = 0.0), 31.1 (η_Q = 0.67).

^{13}C{^{1}H} NMR (126 MHz, 298 K, $CDCl_3$): δ (ppm) = 150.7 (C_q, 3C), 148.2 (C_q, 6C), 147.1 (C_q, 3C), 140.1 (C_q, 12C), 139.9 (C_q, 6C), 135.2 (CH, 6C), 133.4 (C_q, 12C), 129.2 (C_q, 6C), 83.8 (C_q, 12C), 25.1 (CH_3, 24C), 23.5 (CH_3, 6C), 23.0 (CH_3, 12C).

Elemental analysis calcd. [%] for $C_{108}H_{144}B_{10}O_{12}$: C 74.45; H 8.33; found: C 73.93; H 8.59.

Tris(4-(dimesitylboranyl)-2,6-dimethylphenyl)borane (BG1Me)

To a suspension of tris(2,6-dimethyl-4-(trifluoroborate)phenyl)borane, tripotassium salt (100 mg, 0.16 mmol) in THF (80 mL), a solution of mesitylmagnesium bromide in THF (1.4 mL, 1 M, 1.4 mmol) was added. The reaction was stirred for 18 h at ambient temperature and the excess Grignard reagent quenched with MeOH (1 mL). All volatiles were removed under reduced pressure. The remaining solid was washed with 50 mL of hexane, water (3 x 10 mL) and acetone (3 x 10 mL) to give **BG1Me** as a white solid in 41% yield (68 mg, 0.06 mmol).

^{1}H-NMR (500 Hz, 298 K, $CDCl_3$): δ (ppm) = 7.03 (s, 6H), 6.79 (s, 12H), 2.29 (s, 18H), 1.99 (s, 36H), 1.97 (s, 18H).

^{11}B NMR (160 Hz, 298 K, $CDCl_3$): δ (ppm) = N.D.

^{13}C{^{1}H} NMR (126 Hz, 298 K, $CDCl_3$): δ (ppm) = 150.4 (C_q, 3C), 148.2 (C_q, 3C), 142.1 (C_q, 6C), 141.0 (C_q, 12C), 139.8 (C_q, 6C), 138.6 (C_q, 6C), 134.2 (CH, 6C), 128.1 (CH, 12C), 23.5 (CH_3, 6C); 23.1 (CH_3, 12C); 21.4 (CH_3, 6C).

HRMS (APCI pos): calc.: 1071.7488 [m/z]; found: 1071.7478 [MH^+]

Tris(4-(bis(4-bromo-2,6-dimethylphenyl)boranyl)-2,6-dimethylphenyl)-borane (BG1Br)

A solution of 5-bromo-2-iodo-1,3-dimethylbenzene (5.60 mmol, 1.74 g) in THF (20 mL) was cooled to –15 °C and a solution of *iso*propylmagnesium chloride lithium chloride complex in THF (5.54 mmol, 0.8 M, 6.93 mL) was slowly added. The reaction mixture was stirred for 1.5 h at –15 °C and 1 h at ambient temperature. A suspension of tris(2,6-dimethyl-4-(trifluoroboranyl)phenyl)borane, tripotassium salt (0.47 mmol, 0.30 g) in THF (75 mL) was added and the yellow suspension was stirred for 4 d at ambient temperature. The excess Grignard reagent was quenched with MeOH (3 mL) and all volatiles were removed under reduced pressure. The remaining solid was washed with water (3 x 20 mL) and hexane (3 x 20 mL). The yellow solid was extracted with chloroform (120 mL). The solution was concentrated and layered with ethanol. The crystallized white solid was further purified by column chromatography (hexane/CH_2Cl_2) to give **BG1Br** as a white solid in 30% yield (0.14 mmol 206 mg).

^{1}H NMR (500 Hz, 298 K, $CDCl_3$): δ (ppm) = 7.16 (s, 12H), 7.00 (s, 6H), 2.00 (s, 36H), 1.98 (s, 18H).

^{11}B NMR (160 Hz, 298 K, $CDCl_3$): δ (ppm) = N.D.

^{13}C{^{1}H} NMR (126 Hz, 298 K, $CDCl_3$): δ (ppm) = 150.9 (C_q, 3C), 146.9 (C_q, 3C), 143.0 (C_q, 6C), 142.9 (C_q, 12C), 140.1 (C_q, 12C), 135.2 (CH, 6C), 130.4 (CH, 12C), 123.7 (C_q, 6C), 23.3 (CH_3, 6C), 22.9 (CH_3, 12C).

HRMS (APCI neg): calc.: 1460.1051 [m/z]; found: 1460.1090 [M^-]

4,4',4'',4''',4'''',4'''''-((Boranetriyltris(3,5-dimethylbenzene-4,1-diyl))tris-(boranetriyl))hexakis(N,N,3,5-tetramethylaniline) (BG1NMe$_2$)

In a three-necked flask with reflux condenser and dropping funnel, magnesium tunings (4.45 mmol, 0.11 g) were covered with THF. Then, a few drops of a solution of 4-bromo-N,N,3,5-tetramethylaniline (4.37 mmol, 1.00 g) in THF (20 mL) were added. The reaction mixture was heated until the reaction started and the rest of the solution was then added dropwise. The reaction mixture was heated at reflux for 1 h and then cooled to rt. The Grignard reagent was slowly added to a white suspension of tris(2,6-dimethyl-4-(trifluoroboranyl)phenyl)borane, tripotassium salt (0.36 mmol, 0.23 g) in THF (50 mL). The orange reaction mixture was stirred for 3 days at ambient temperature and then all volatiles were removed under reduced pressure. The green solid was slurried in a 1:1 CH_2Cl_2:hexane solution and filtered and all volatiles removed under reduced pressure. The remaining solid was purified by column chromatography (hexane/CH_2Cl_2 with 1% Et_3N as an additive). The product was dissolved in 10 mL of CH_2Cl_2 and precipitated with ethanol to give **BG1NMe$_2$** as a yellow solid in 79% yield (0.28 mmol, 354 mg).

^{1}H NMR (500 Hz, 298 K, $CDCl_3$): δ (ppm) = 7.06 (s, 6H), 6.36 (s, 12H), 2.97 (s, 36H), 2.00 (s, 18H), 1.98 (s, 36H).

^{11}B NMR (160 Hz, 298 K, $CDCl_3$): δ (ppm) = N.D.

^{13}C{^{1}H} NMR (126 Hz, 298 K, $CDCl_3$): δ (ppm) = 150.9 (C_q, 6C), 150.2 (C_q, 3C), 149.6 (C_q, 6C), 143.0 (C_q, 12C), 139.5 (C_q, 6C), 138.7 (C_q, 6C), 134.6 (CH, 6C), 111.4 (CH, 12C), 40.3 (CH_3, 12C), 24.4 (CH_3, 6C), 23.1 (CH_3, 12C).

HRMS (APCI neg): calc.: 1244.9013 [m/z]; found: 1244.9023 [M^-]

Tris(4-(bis(2,6-bis(trifluoromethyl)phenyl)boranyl)-2,6-dimethylphenyl)-borane (BFG1H)

A solution of **BFD1H** (1.50 mmol, 1.00 g) in diethyl ether (25 mL) was cooled to –78 °C. Then *n*BuLi (1.65 mmol, 1.6 M, 1.10 mL) was added dropwise and the reaction stirred for 3 h at –78 °C. Then $BF_3(OEt_2)$ (0.50 mmol, 0.06 mL) in diethyl ether (12 mL) was slowly added and the solution slowly warmed to ambient temperature. The reaction was stirred for 3 days at ambient temperature and then quenched with MeOH (2 mL). All volatiles were removed under reduced pressure. The remaining solid was extracted with hexane and toluene. The solvent was evaporated under reduced pressure and the product was purified by column chromatography (hexane/CH_2Cl_2), to give **BFG1H** as a white solid in 39% yield (0.2 mmol, 319 mg).

^{1}H NMR (500 Hz, 298 K, $CDCl_3$): δ (ppm) =8.01 (d, ^{3}J = 8 Hz, 12H), 7.96 (*t*, 6H, ^{3}J = 8 Hz), 6.71 (*s*, 6H), 1.90 (*s*, 18H).

^{11}B SS NMR (128.3 MHz): δ (ppm) = 77.4 (η_Q = 0.0); 71.0 (η_Q = 0.16); 69.6 (η_Q = 0.13).

^{13}C{^{1}H} NMR (126 Hz, 298 K, $CDCl_3$): δ (ppm) = 151.0 (C_q, 3C), 147.6 (C_q, 3C), 139.1 (C_q, 6C), 139.0 (C_q, 6C), 136.1 (CH, 6C), 130.2 (CH, 12C), 130.0 (CH, br, 6C, assignment by HSQC) 124.0 (CF_3, 12C, $^{1}J_{CF}$ = 275 Hz); 22.5 (CH_3, 6C).

^{19}F NMR (470.6 Hz, 298 K, $CDCl_3$): δ (ppm) = –50.46 (br.), 55.50 (br.).

HRMS (APCI neg): calc.: 1633.3115 [m/z]; found: 1633.3105 [M^-]

Tris(4-(bis(2,6-dimethyl-4-(trifluoroborate)phenyl)boraneyl)-2,6-dimethylphenyl)borane, hexa(tetrabutylammonium) salt (BG1BF$_3$N(*n*Bu)$_4$)

To a suspension of **BG1Bpin** (0.12 mmol, 206 mg) in $CHCl_3$ (50 mL), a solution of [N(*n*Bu$_4$)][HF_2] (2.13 mmol, 599 mg) was added. Shortly after addition the residual solid dissolved. The reaction was stirred for 1 h. Then all volatiles were removed *in vacuo*. The resulting oil was washed with water (3 x 30 mL) to give the tris(4-(bis(2,6-dimethyl-4-(trifluoroborate)phenyl)boraneyl)-2,6-dimethylphenyl)borane,hexa(tetra-butylammonium) salt as light yellow solid in 92% yield (0.11 mmol, 310 mg).

^{1}H NMR (500 Hz, 298 K, CD_2Cl_2): δ (ppm) = 7.06 (s, 12H), 7.01 (s, 6H), 3.13 – 3.04 (m, 48H), 2.01 – 1.96 (m, 54H), 1.60 – 1.48 (m, 48H), 1.35 (sex., ^3J = 7 Hz, 48H), 0.95 (t, ^3J = 7 Hz, 72H).

^{11}B NMR (160 Hz, 298 K, CD_2Cl_2): δ (ppm) = 2.8 (broad).

^{13}C{^{1}H NMR (126 Hz, 298 K, CD_2Cl_2): δ (ppm) = 149.9 (C_q, 3C), 149.9 (C_q, 3C), 143.0 (C_q, 6C), 139.7 (C_q, 6C), 138.7 (C_q, 12C), 134.8 (CH, 6C), 131.2 (CH, 12C), 75.1 (CH_2, 24C), 24.1 (CH_2, 24C), 23.8 (CH_3, 12C), 23.2 (CH_3, 6C), 20.0 (CH_2, 24C), 13.7 (CH_3, 24C).

^{19}F NMR (470.6 Hz, 298 K, CD_2Cl_2): δ (ppm) = –141.19 (s, br).

HRMS (ESI neg): calc.: 325.9852 [m/z]; found: 325.9847 [M^{5-}(N(*n*Bu$_4$))]

calc.: 468.0525 [m/z]; found: 468.0517 [M^{4-}(N(*n*Bu$_4$))$_2$]

calc.: 704.8314 [m/z]; found: 704.8314 [M^{3-}(N(*n*Bu$_4$))$_3$]

Elemental analysis calcd. [%] for $C_{168}H_{300}B_{10}F_{18}N_6$: C 70.69; H 10.59; N 2.94; found: C 70.94 H 10.15 N 2.63.

Tris(4-(bis(4-(bis(2,6-dimethylphenyl)boraneyl)-2,6-dimethylphenyl)boraneyl)-2,6-dimethylphenyl)borane (BG2H)

To a suspension of **BG1BF$_3$N(*n*Bu)$_4$** (0.04 mmol, 100 mg) in THF (40 mL), a solution of mesitylmagnesium bromide in THF (0.8 mL, 0.8 M, 0.64 mmol) was added. The reaction was stirred for 2 d at ambient temperature. Then the excess Grignard reagent was quenched with aqueous Et_2O (5 mL) and all volatiles were removed *in vacuo*. The resulting solid was extracted with toluene (3 x 20 mL). The solvent was removed under reduced pressure and the raw product purified by column chromatography (hexane:CH_2Cl_2) to give **BG2H** as a white solid in 31% yield (0.01 mmol, 25 mg).

^{1}H NMR (500 Hz, 298 K, CD_2Cl_2): δ (ppm) = 7.17 (t, ^{3}J = 8 Hz, 12H), 7.09 (s, 12H), 7.05 (s, 6H), 6.98 (d, ^{3}J = 8 Hz, 24H), 2.05 (s, 72H), 2.01 (s, 36H), 1.98 (s, 18H).

^{11}B NMR (160 Hz, 298 K, CD_2Cl_2): δ (ppm) = N.D.

^{13}C{^{1}H} NMR (126 Hz, 298 K, CD_2Cl_2): δ (ppm) =151.1 (C_q, 3C), 149.5 (C_q, 6C), 147.2 (C_q, 3C), 147.0 (C_q, 6C), 145.2 (C_q, 12C), 141.1 (C_q, 24C), 140.5 (C_q, 6C), 140.3 (C_q, 12C), 135.6 (CH, 6C), 135.0 (CH, 12C), 129.2 (CH, 12C), 127.6 (CH, 24C), 23.6 (CH_3, 24C), 23.2 (CH_3, 12C), 23.1 (CH_3, 6C).

HRMS (APCI neg): calc.: 2307.5129 [m/z]; found: 2307.5156 [M^{-}]

Elemental analysis calcd. [%] for $C_{174}H_{194}B_{10}$: C 87.31; H 8.17; found: C 86.43; H 8.28.

NMR Spectra

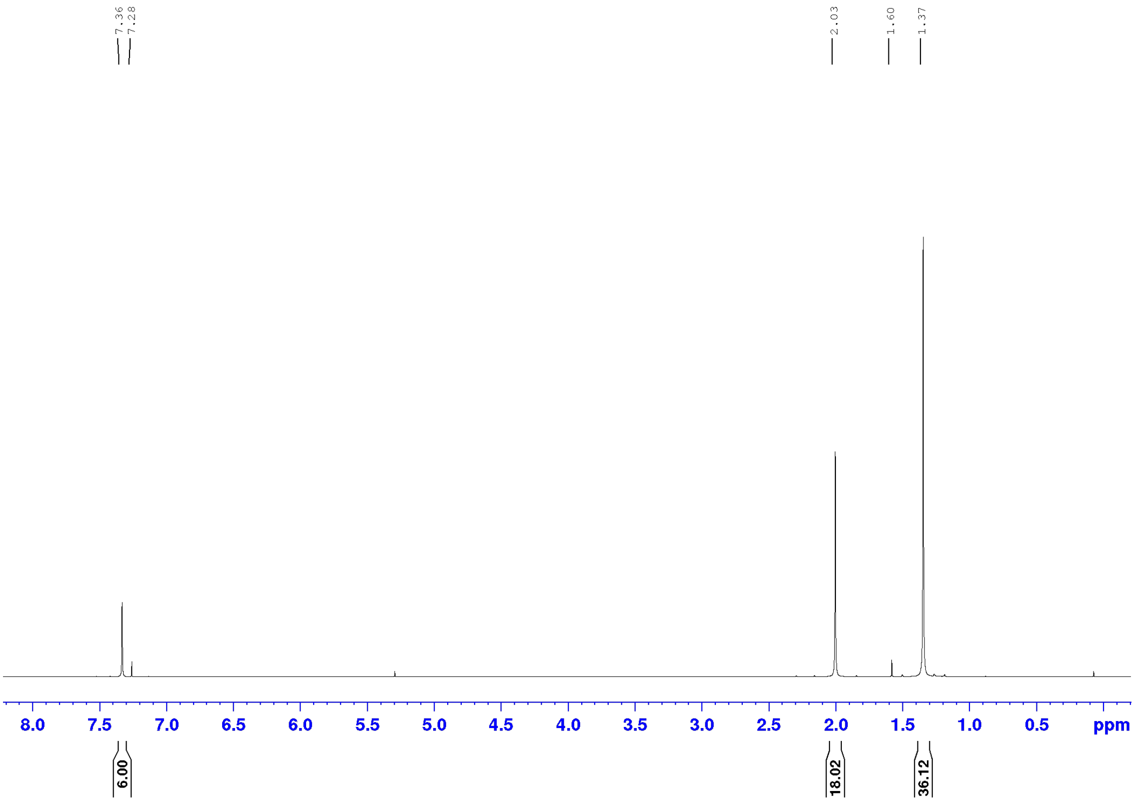

Figure S1: ^{1}H NMR spectrum (400 MHz, 298 K, $CDCl_3$) of tris(2,6-dimethyl-4-(4,4,5,5-tetramethyl-1,3,2-dioxaborolan-2-yl)phenyl)borane.

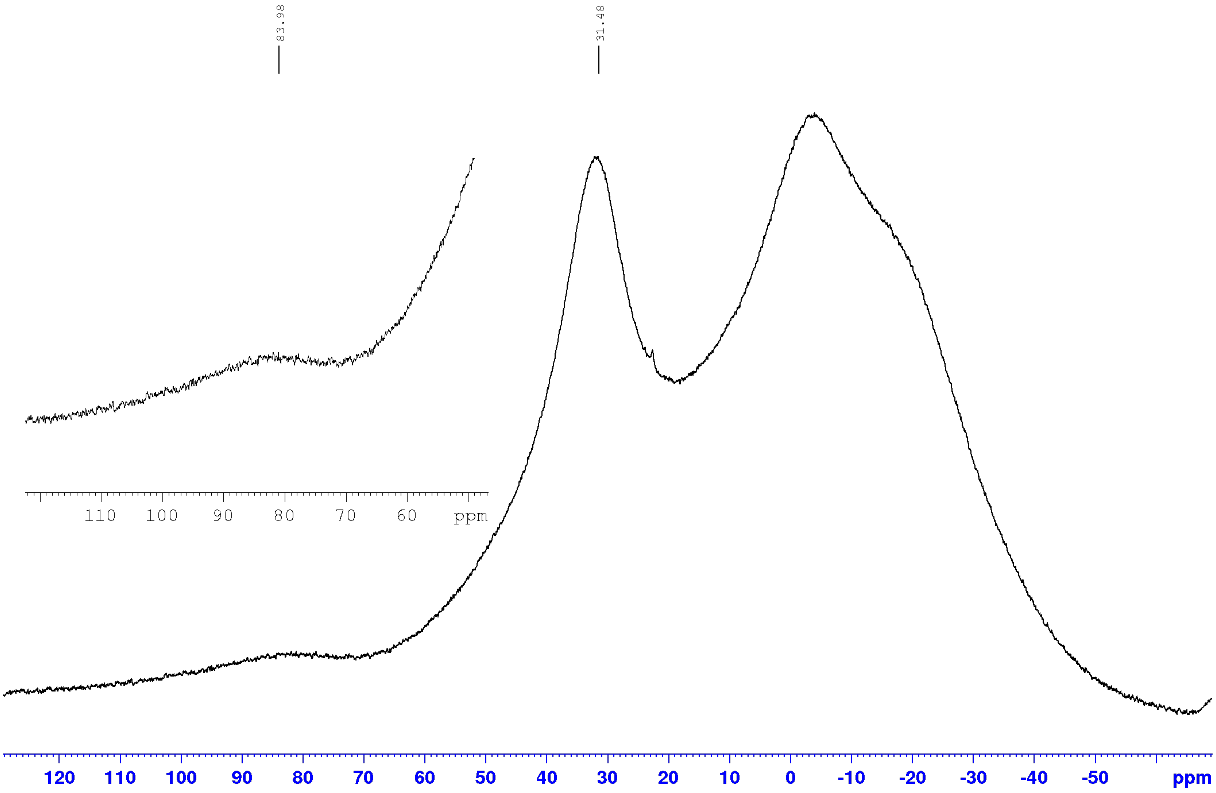

Figure S2: ^{11}B NMR spectrum (128 MHz, 298 K, $CDCl_3$) of tris(2,6-dimethyl-4-(4,4,5,5-tetramethyl-1,3,2-dioxaborolan-2-yl)phenyl)borane.

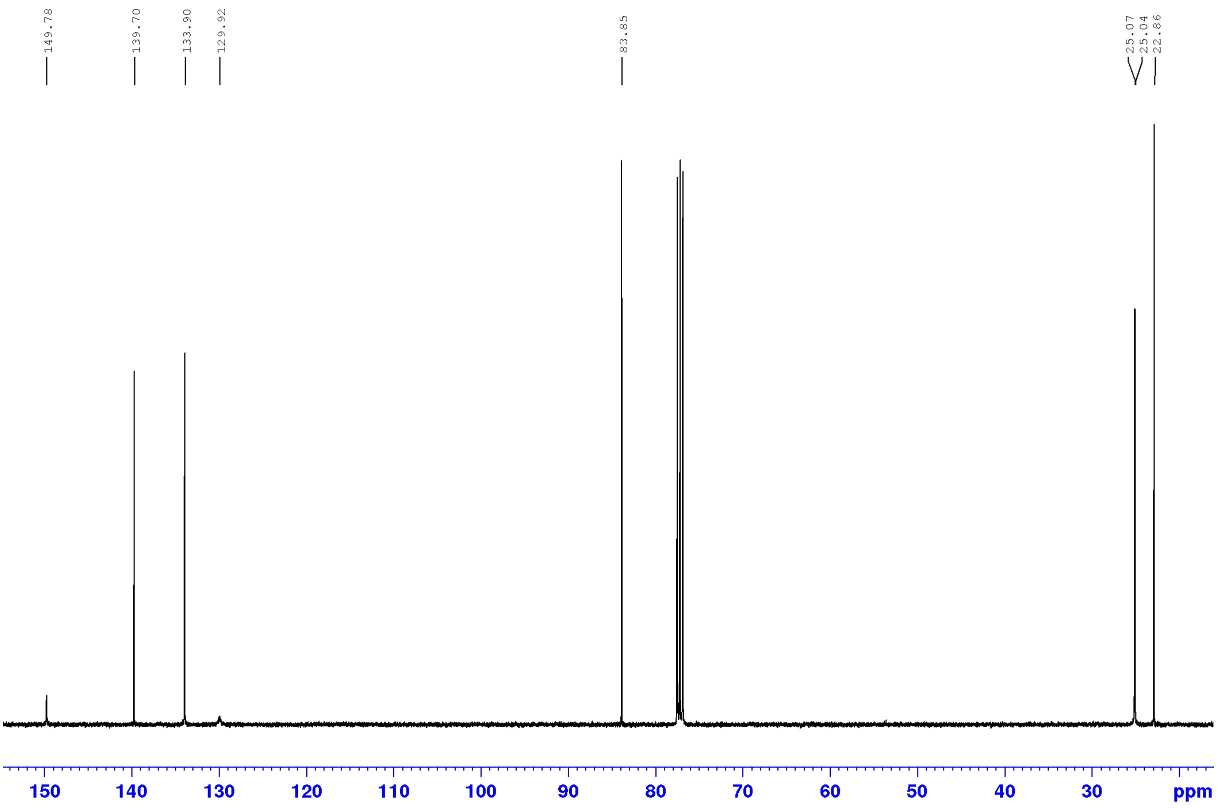

Figure S3: $^{13}C\{^{1}H\}$ NMR spectrum (101 MHz, 298 K, $CDCl_3$) of tris(2,6-dimethyl-4-(4,4,5,5-tetramethyl-1,3,2-dioxaborolan-2-yl)phenyl)borane.

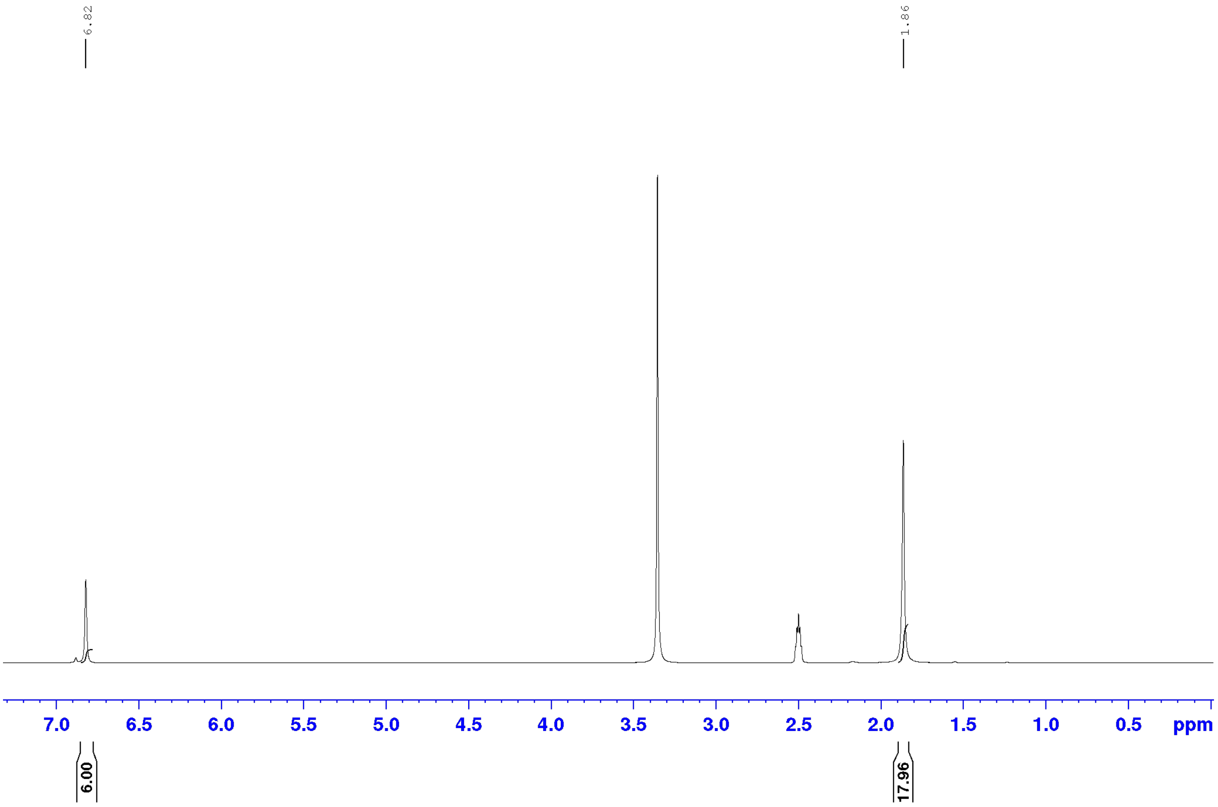

Figure S4: ^{1}H NMR spectrum (200 MHz, 298 K, DMSO-d_6) of tris(2,6-dimethyl-4-(trifluoroboranyl)phenyl)borane, tripotassium salt.

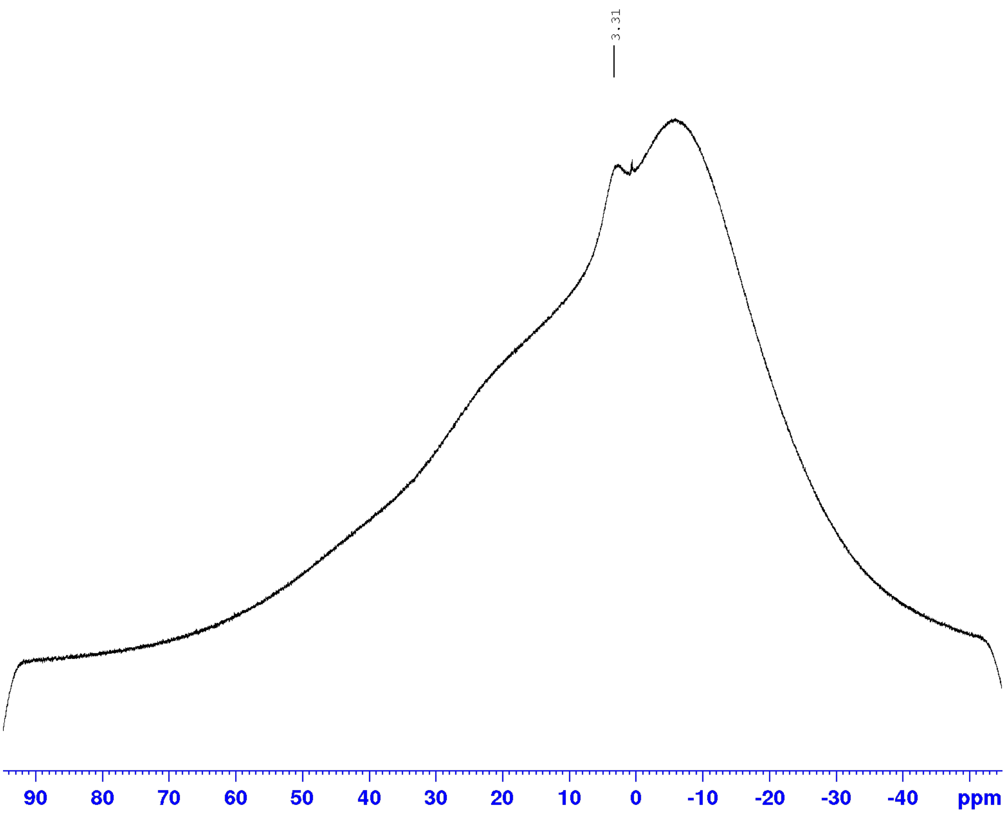

Figure S5: ^{11}B NMR spectrum (95 MHz, 298 K, DMSO-d_6) of tris(2,6-dimethyl-4-(trifluoroboranyl)phenyl)borane, tripotassium salt.

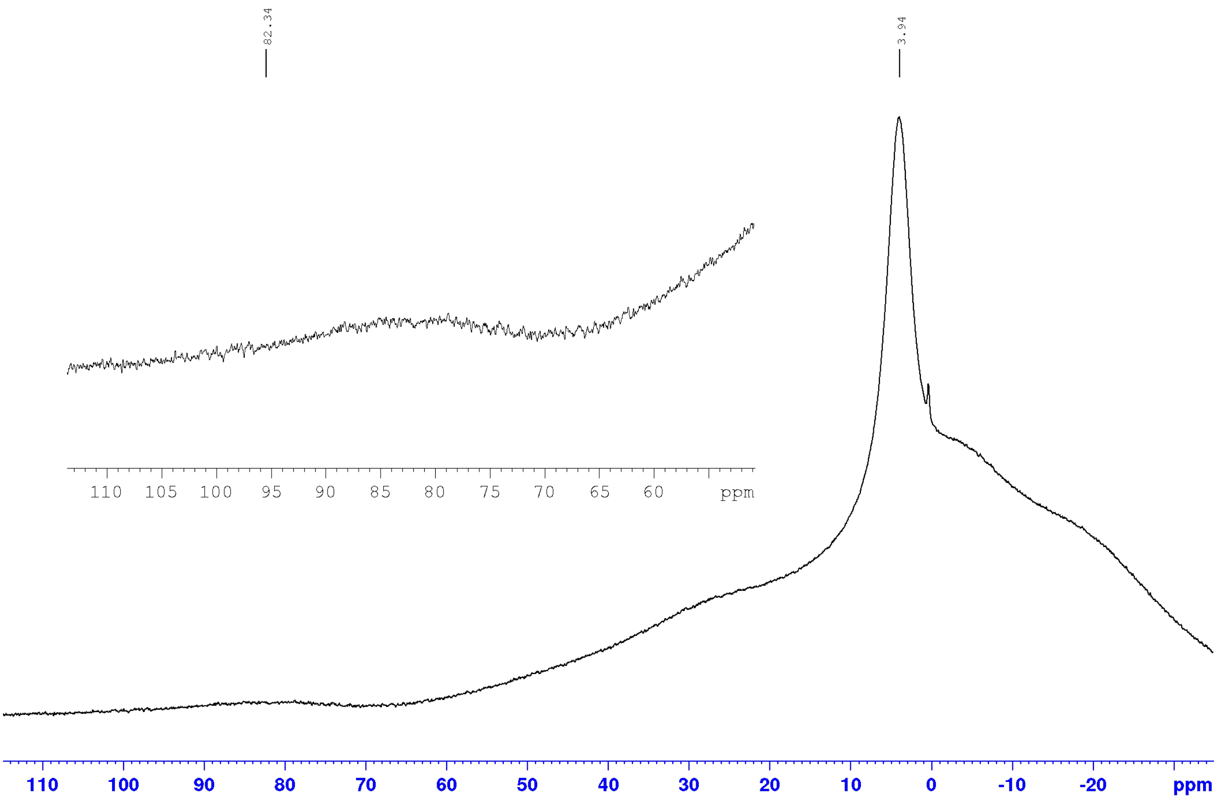

Figure S6: ^{11}B NMR spectrum (128 MHz, 298 K, D_2O) of tris(2,6-dimethyl-4-(trifluoroboranyl)phenyl)borane, tripotassium salt.

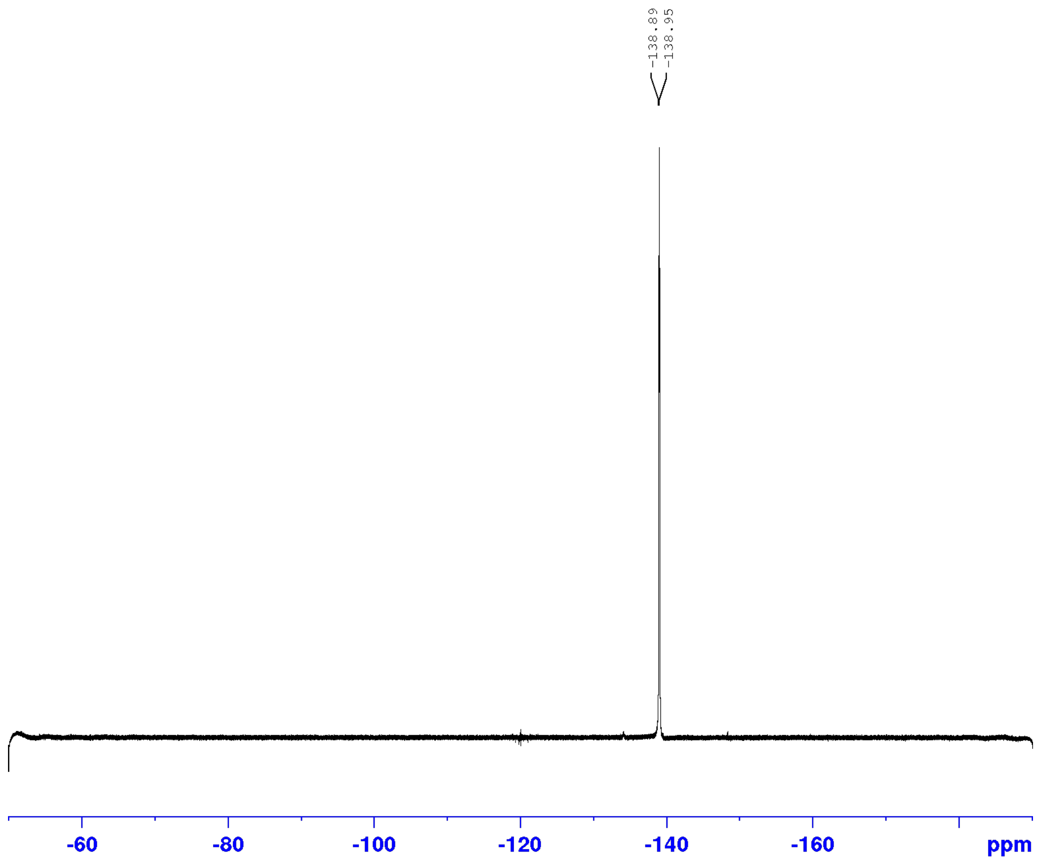

Figure S7: ^{19}F NMR spectrum (471 MHz, 298 K, DMSO-d_6) of tris(2,6-dimethyl-4-(trifluoroboranyl)phenyl)borane, tripotassium salt.

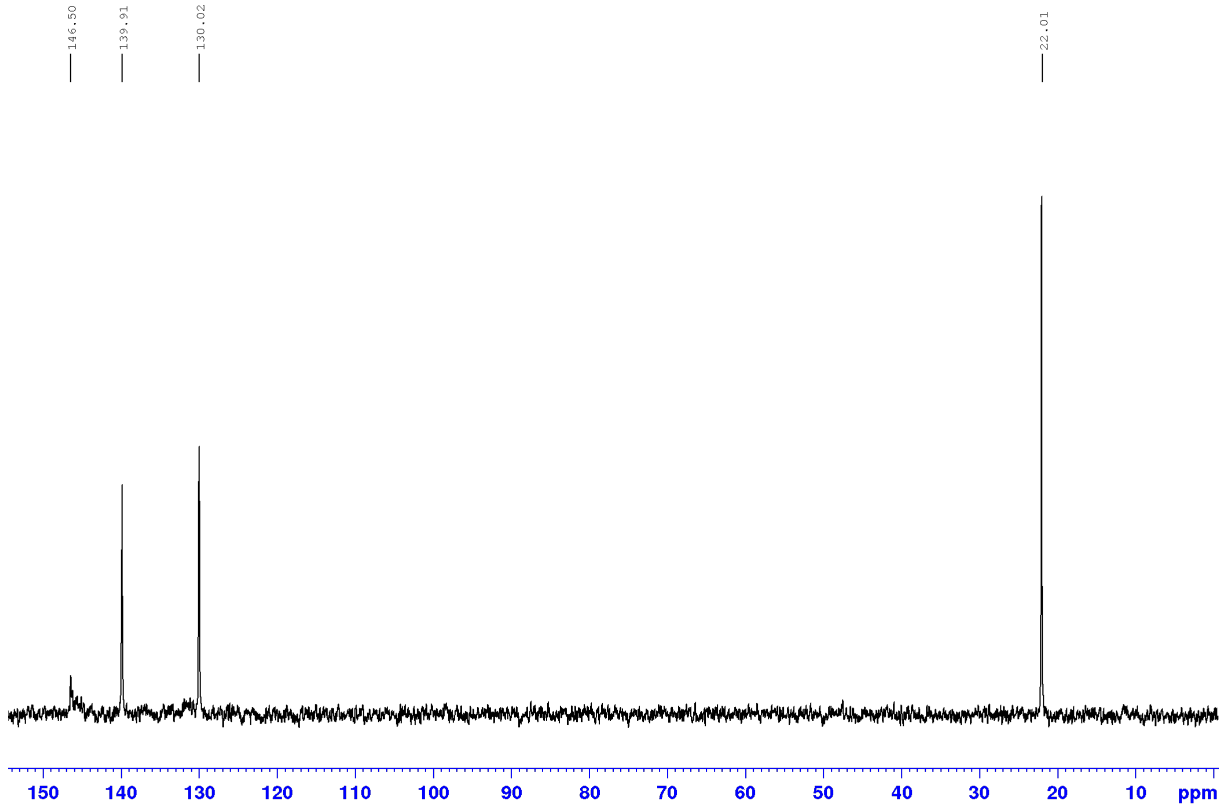

Figure S8: ^{13}C{^{1}H} NMR spectrum (101 MHz, 298 K, D_2O) of tris(2,6-dimethyl-4-(trifluoroboranyl)phenyl)borane, tripotassium salt.

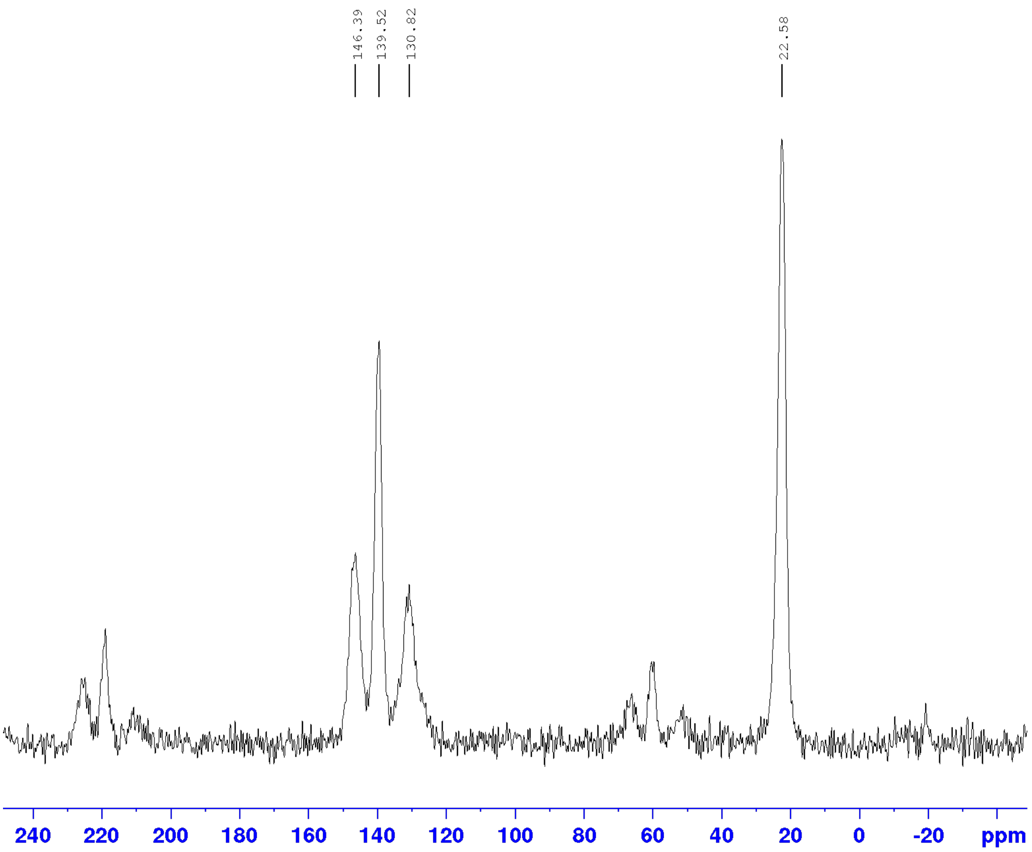

Figure S9: ^{13}C-SS NMR spectrum (248.8 MHz, 298 K) of tris(2,6-dimethyl-4-(trifluoroboranyl)phenyl)borane, tripotassium salt.

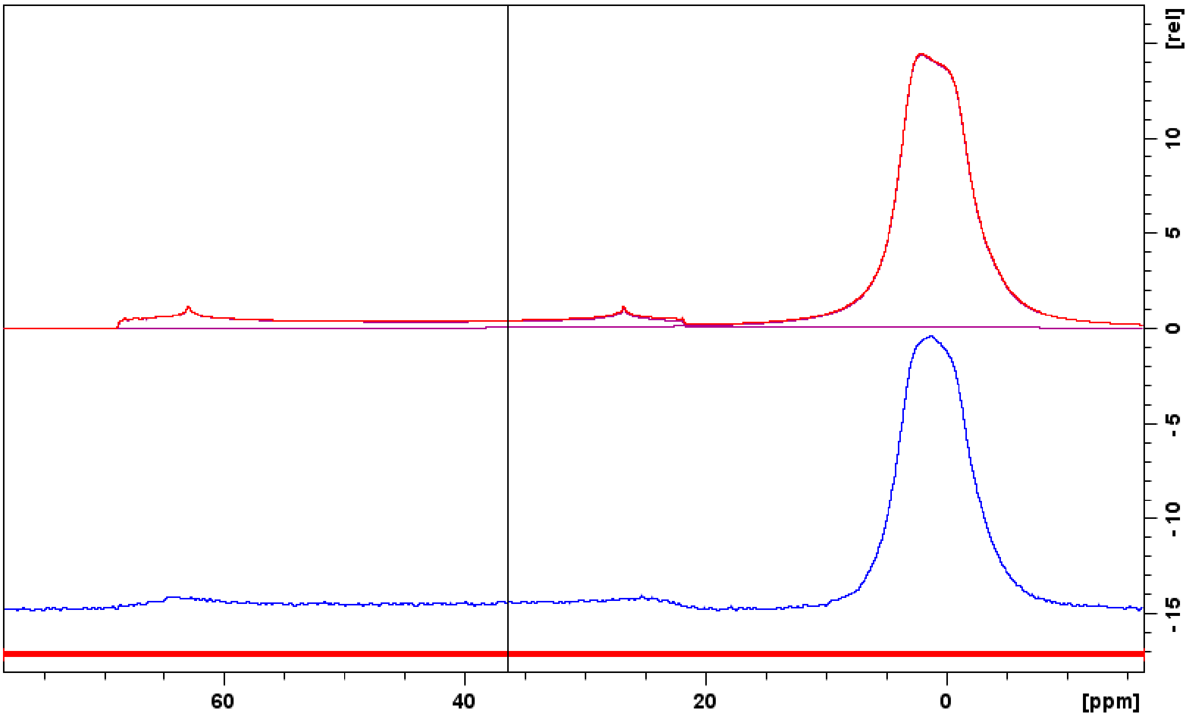

Figure S10: ^{11}B-SS NMR spectrum (128.3 MHz, 298 K) of tris(2,6-dimethyl-4-(trifluoroboranyl)phenyl)borane, tripotassium salt (bottom). Simulated spectrum (top).

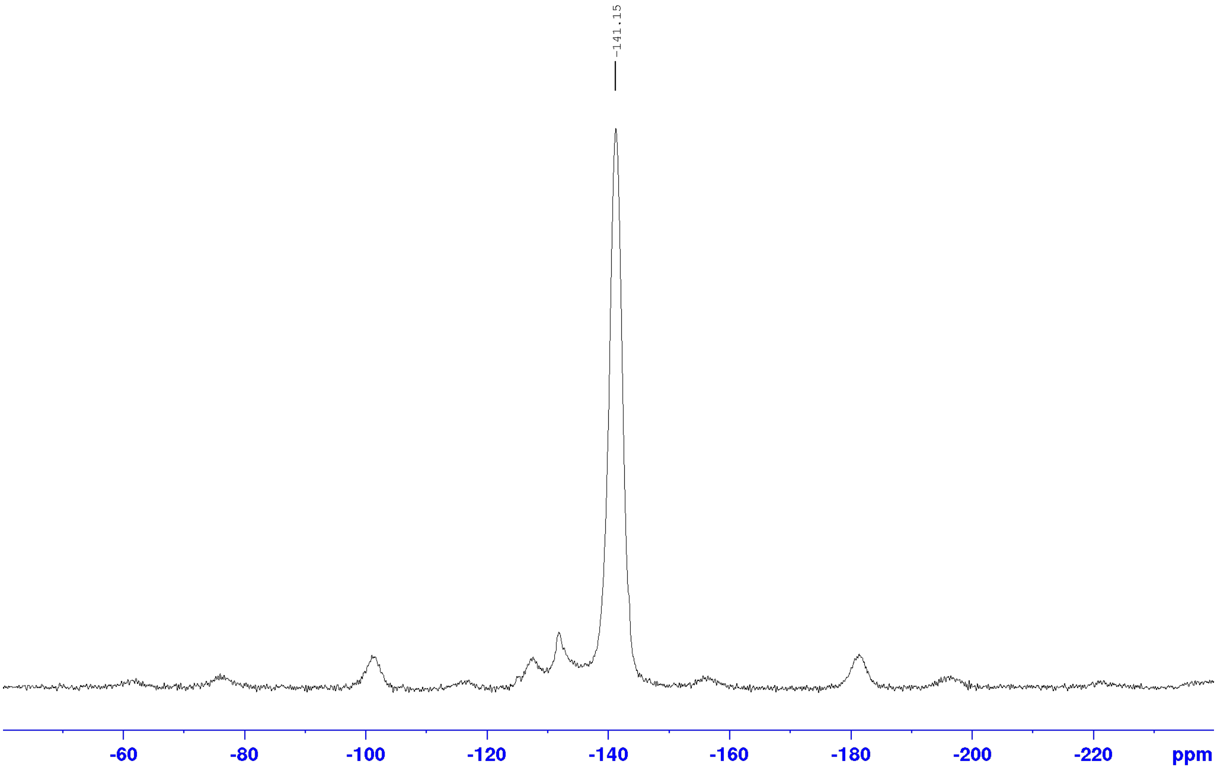

Figure S11: ^{19}F-SS NMR spectrum (376.5 MHz, 298 K) of tris(2,6-dimethyl-4-(trifluoroboranyl)phenyl)borane, tripotassium salt

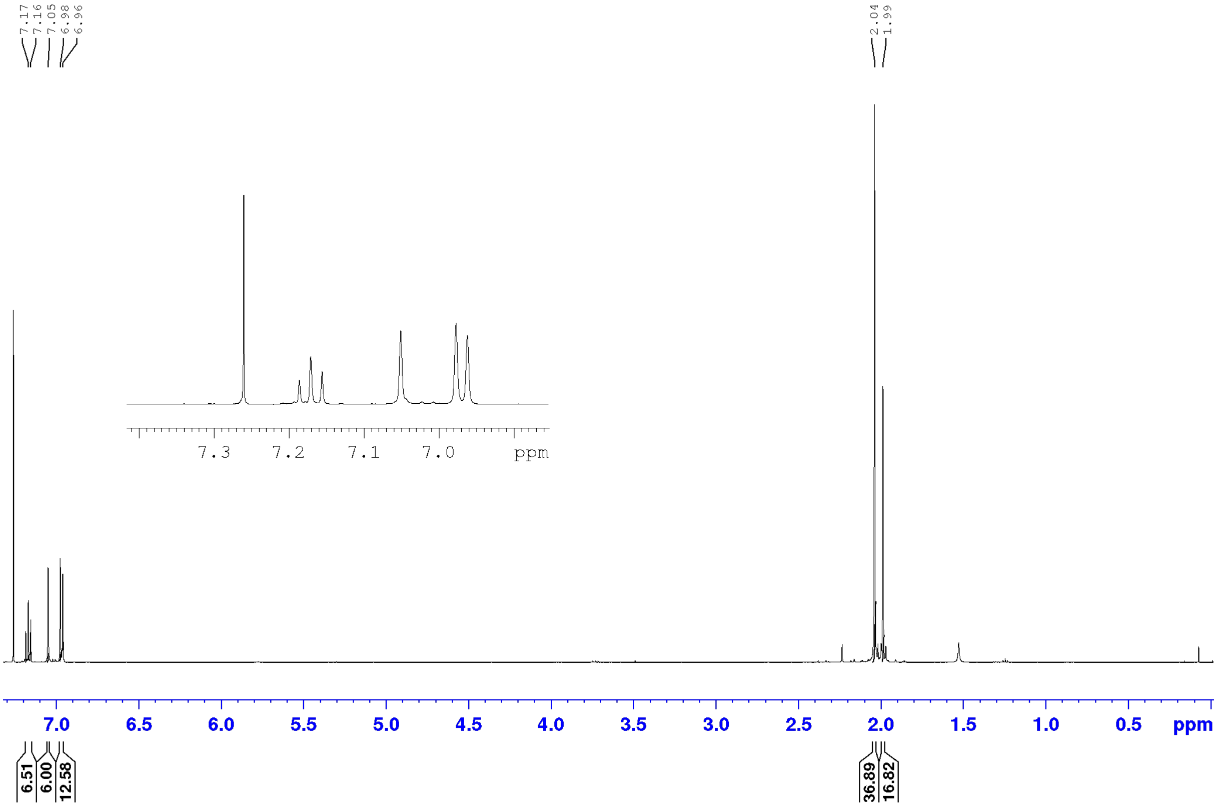

Figure S12: ^{1}H NMR spectrum (500 MHz, 298 K, $CDCl_3$) of **BG1H**.

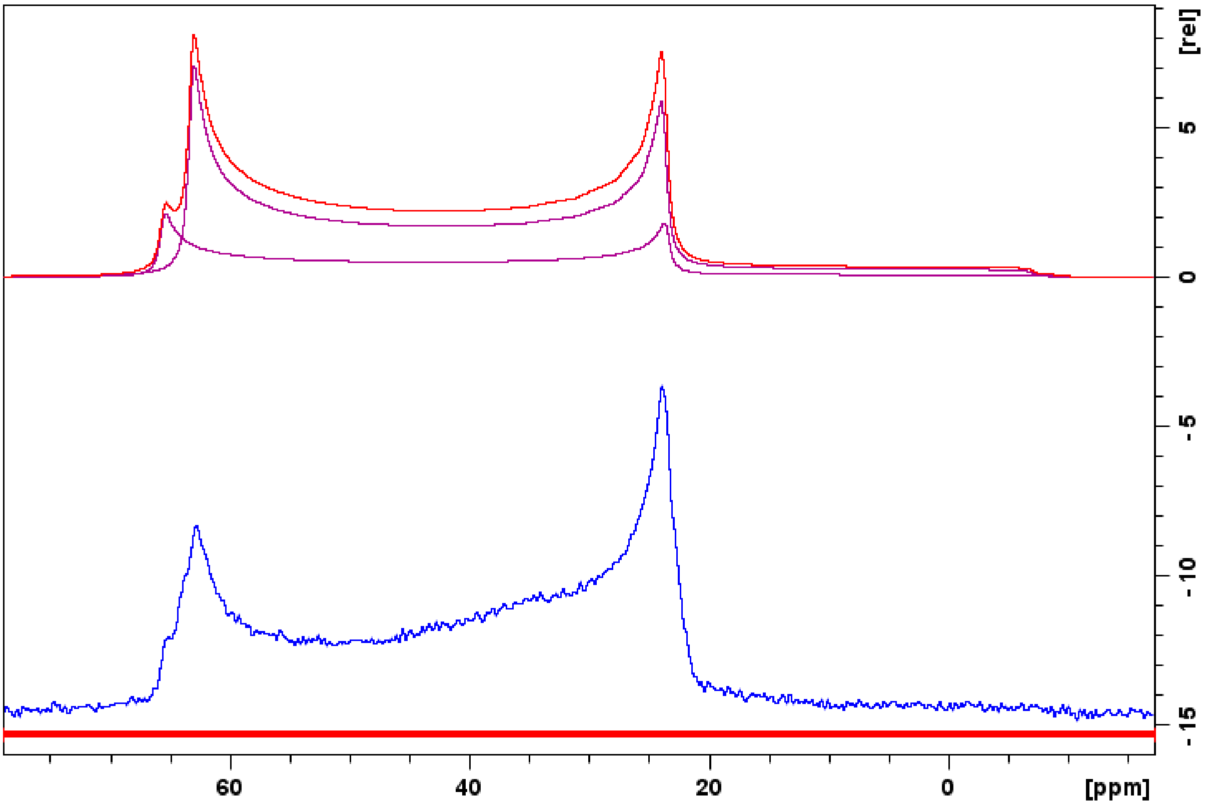

Figure S13: ^{11}B-SS NMR spectrum (128.3 MHz, 298 K) of **BG1H** (bottom). Simulated spectrum (top).

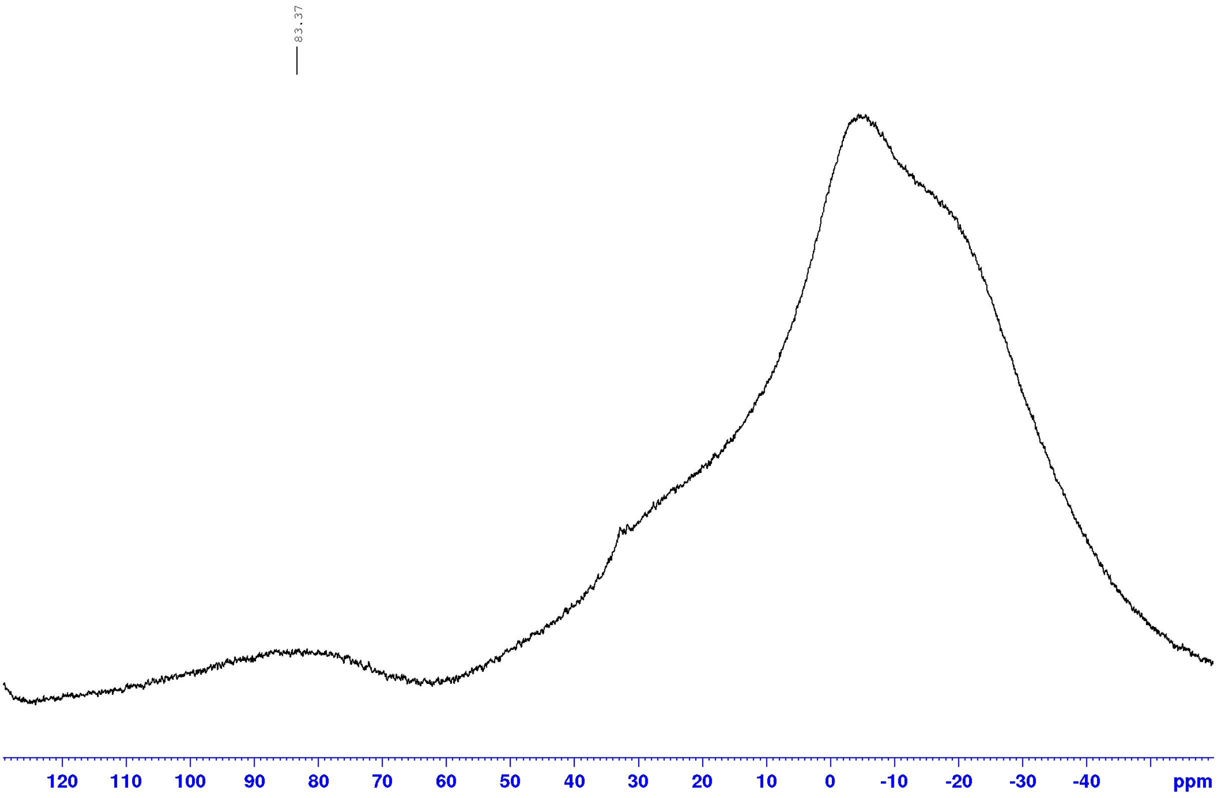

Figure S14: ^{11}B NMR spectrum (128 MHz, 298 K, $CDCl_3$) of **BG1H**.

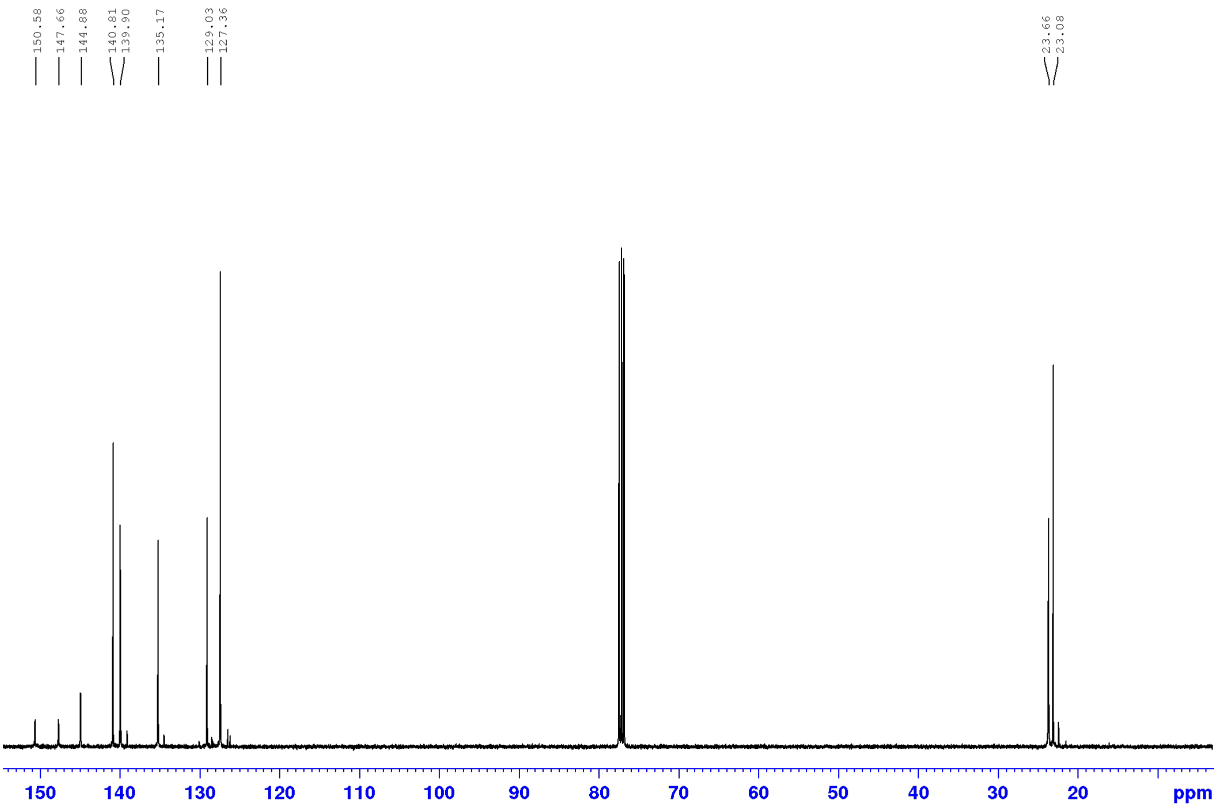

Figure S15: $^{13}C\{^{1}H\}$ NMR spectrum (126 MHz, 298 K, $CDCl_3$) of **BG1H**.

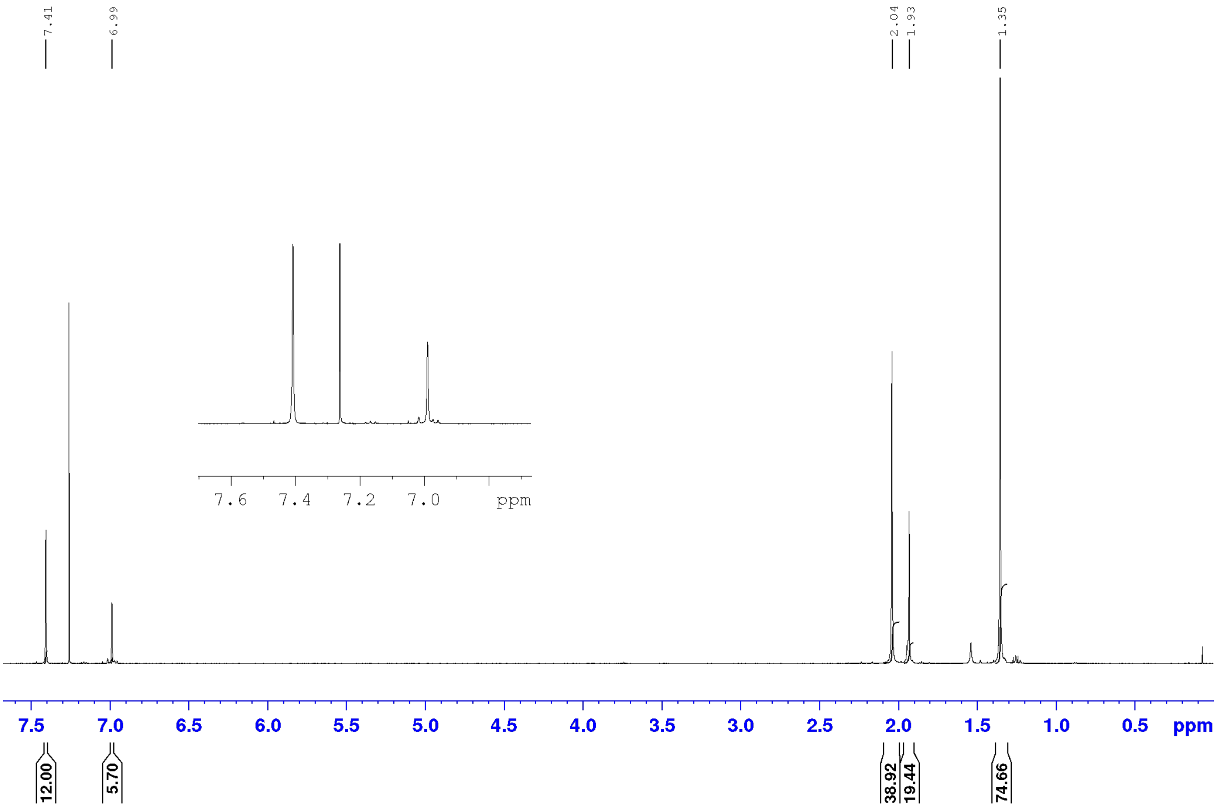

Figure S16: ^{1}H NMR spectrum (500 MHz, 298 K, $CDCl_3$) of **BG1Bpin**.

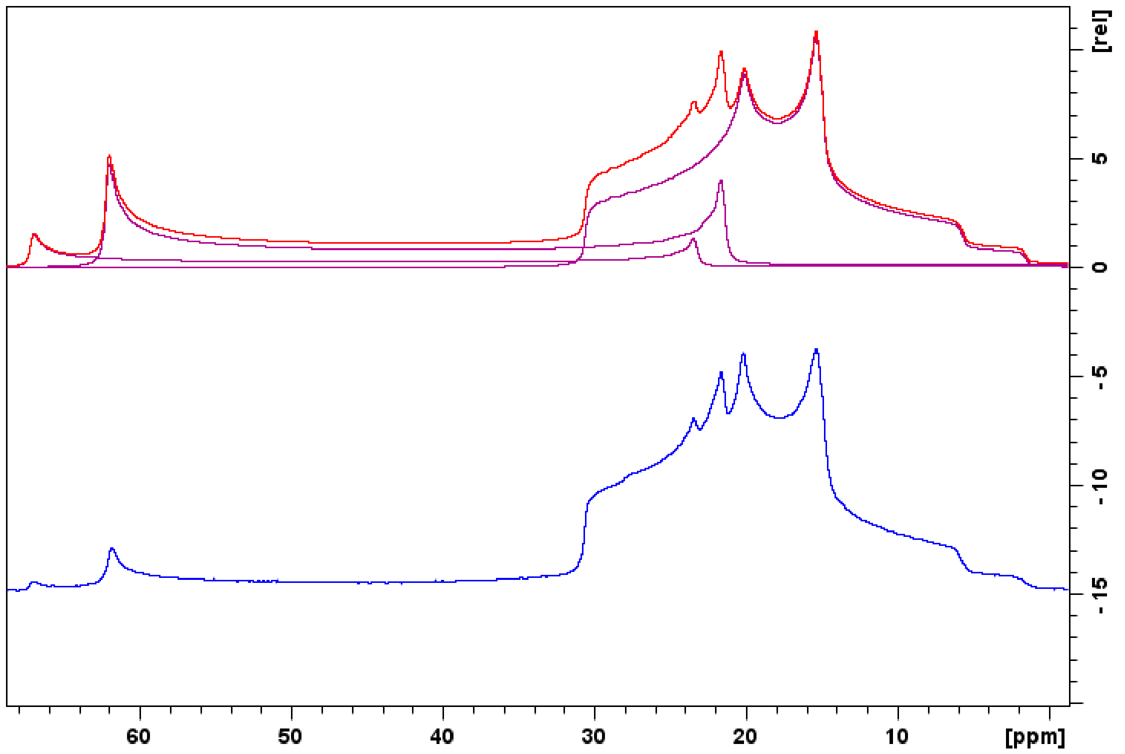

Figure S17: ^{11}B-SS NMR spectrum (128.3 MHz, 298 K) of **BG1Bpin** (bottom). Simulated spectrum (top)

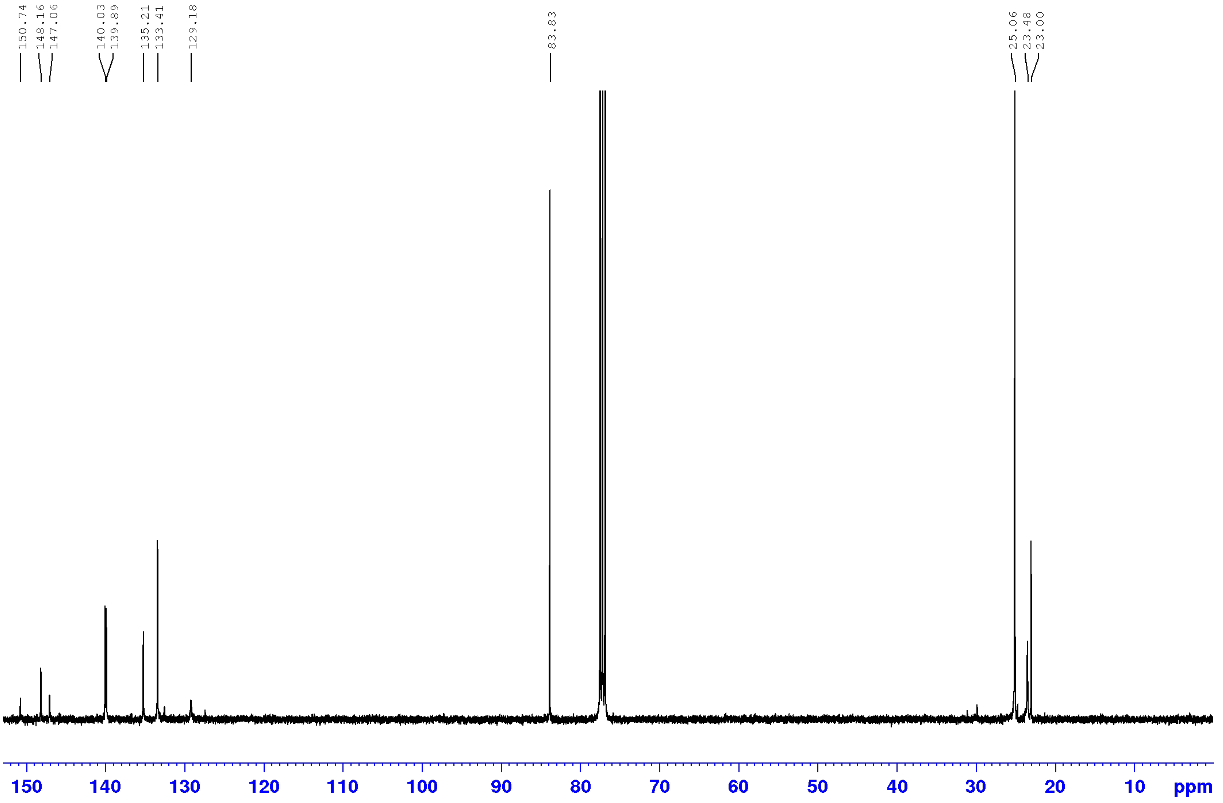

Figure S18: $^{13}C\{^{1}H\}$ NMR spectrum (126 MHz, 298 K, $CDCl_3$) of **BG1Bpin**.

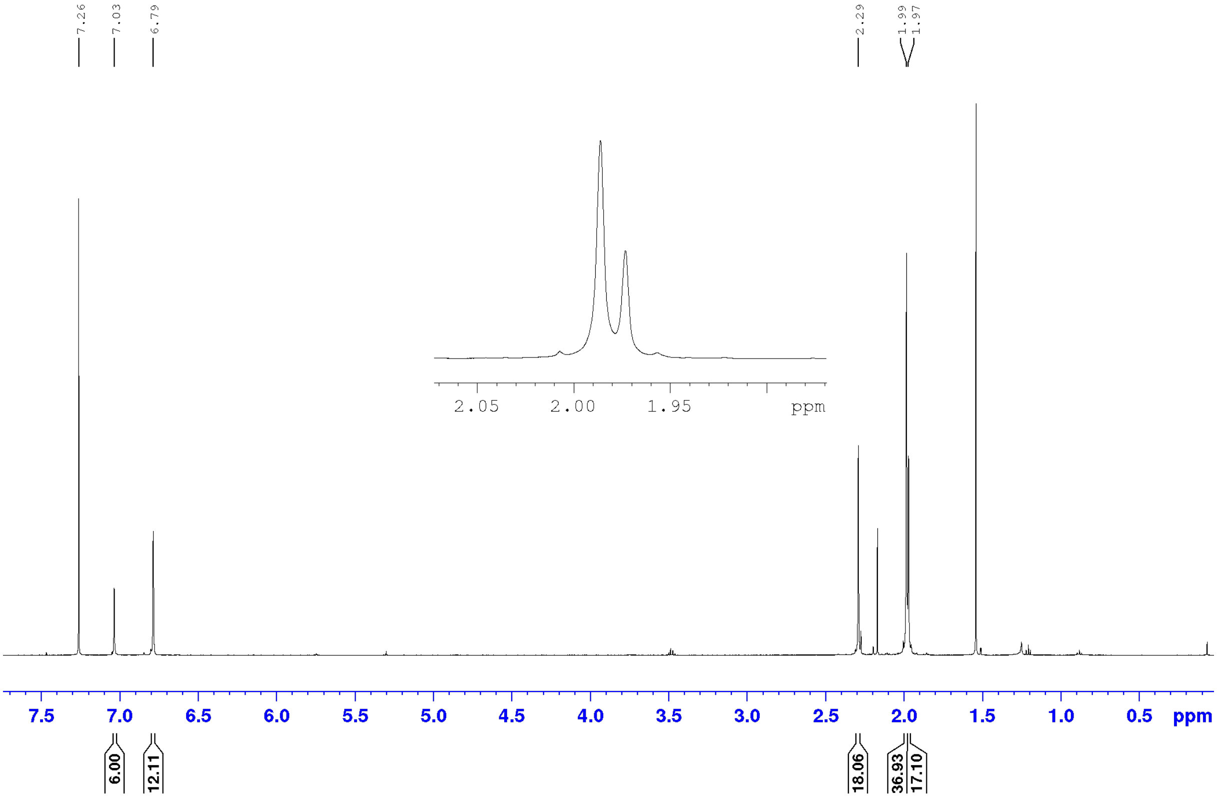

Figure S19: ^{1}H NMR spectrum (500 MHz, 298 K, $CDCl_3$) of **BG1Me**.

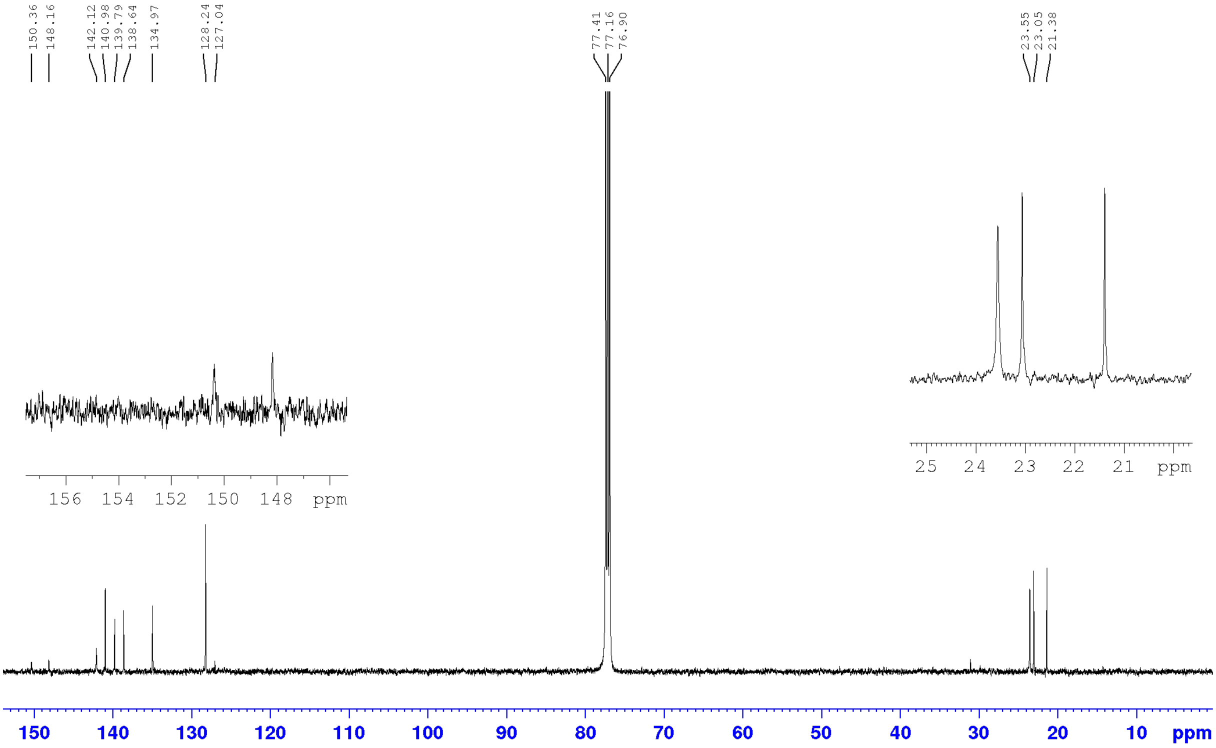

Figure S20: ^{13}C{^{1}H} NMR spectrum (126 MHz, 298 K, $CDCl_3$) of **BG1Me**.

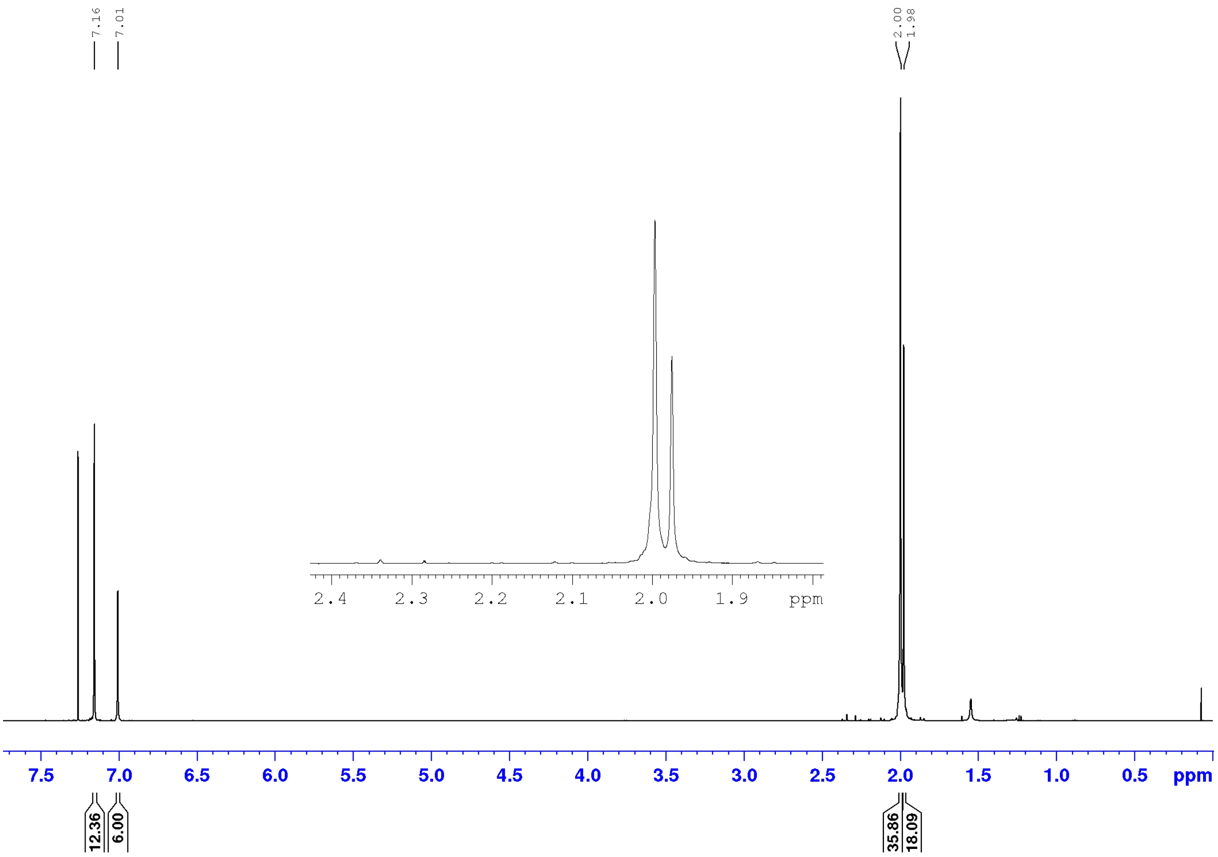

Figure S21: ^{1}H NMR spectrum (500 MHz, 298 K, $CDCl_3$) of **BG1Br**.

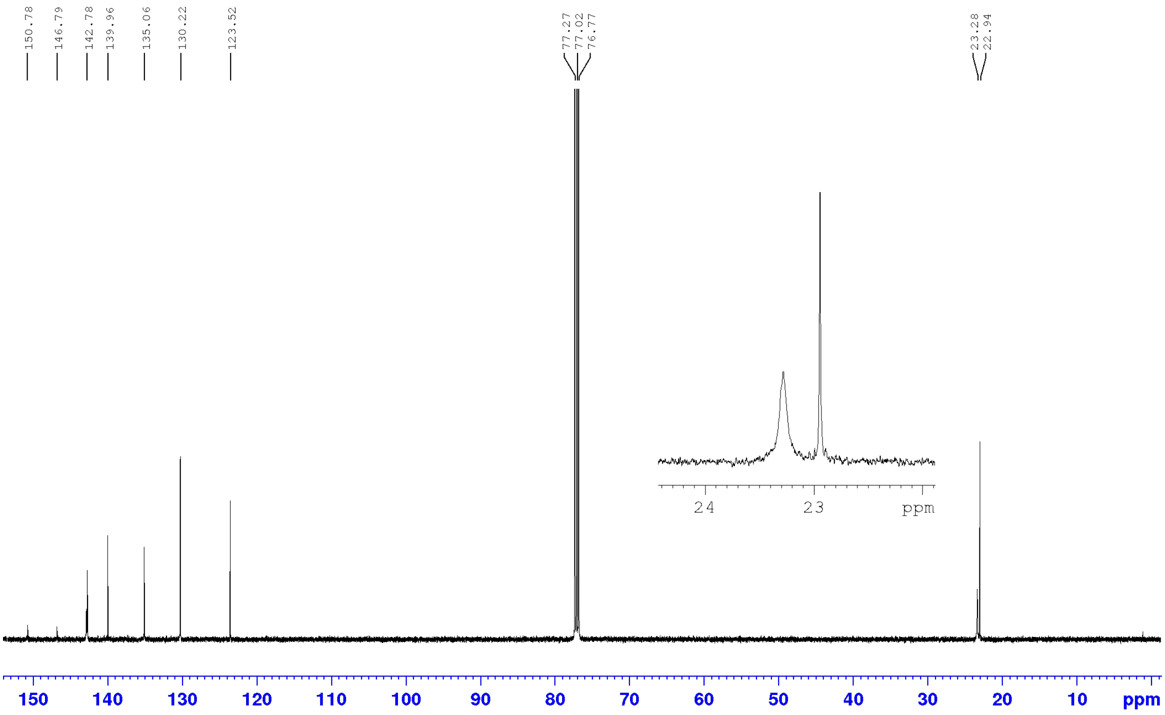

Figure S22: $^{13}C\{^{1}H\}$ NMR spectrum (126 MHz, 298 K, $CDCl_3$) of **BG1Br**.

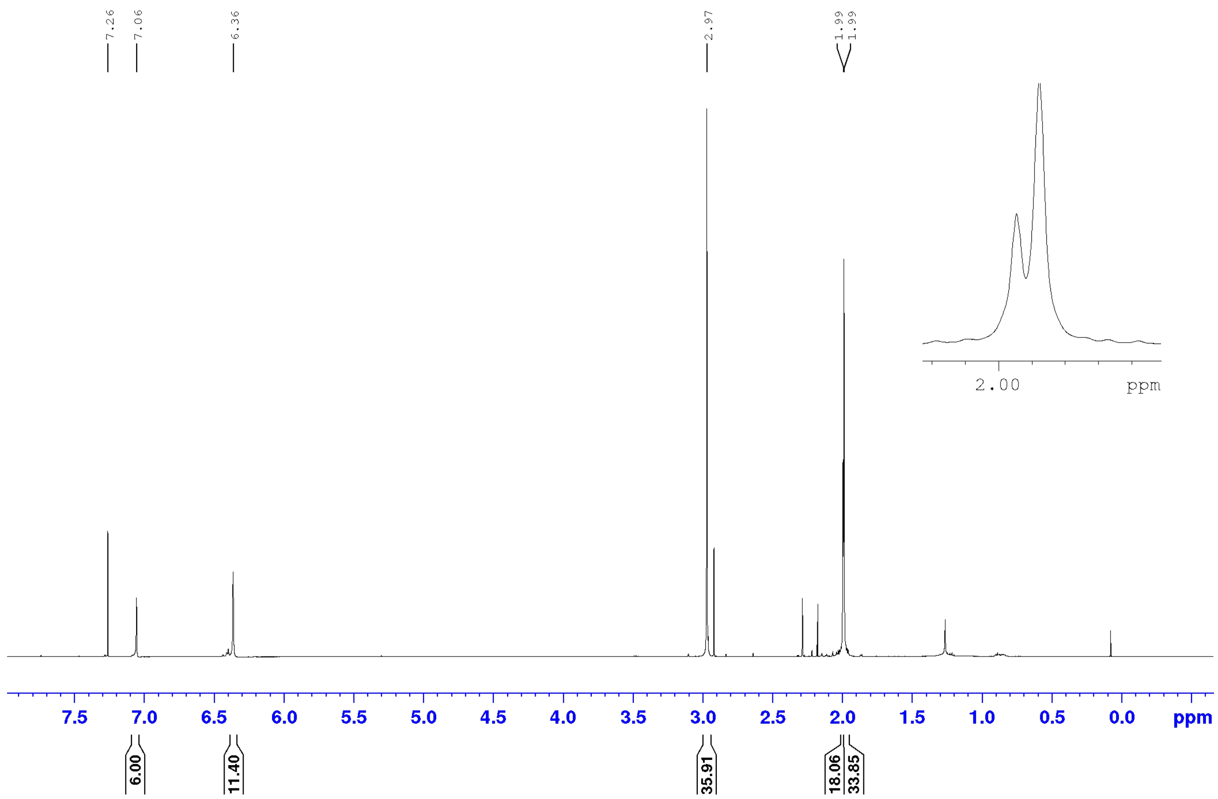

Figure S23: ^{1}H NMR spectrum (500 MHz, 298 K, $CDCl_3$) of **BG1NMe2**.

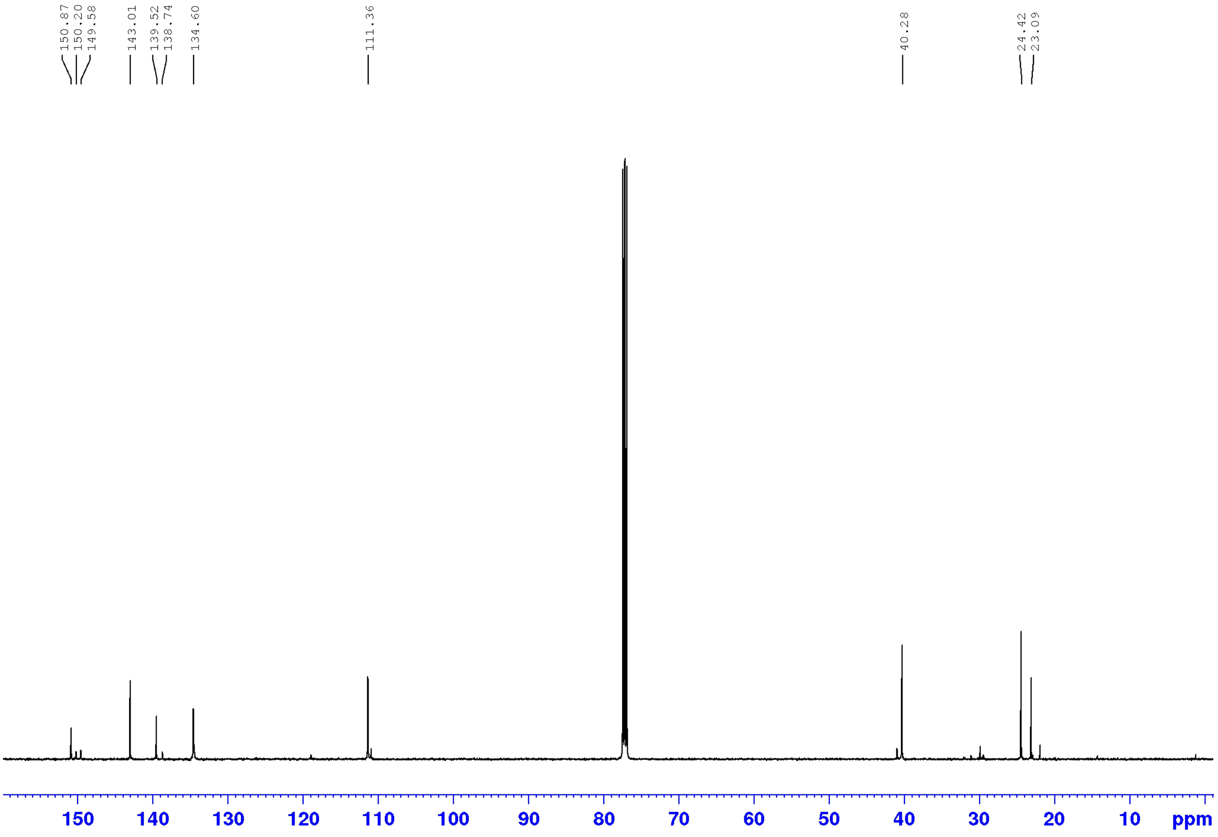

Figure S24: ^{13}C{^{1}H} NMR spectrum (126 MHz, 298 K, $CDCl_3$) of **BG1NMe2**.

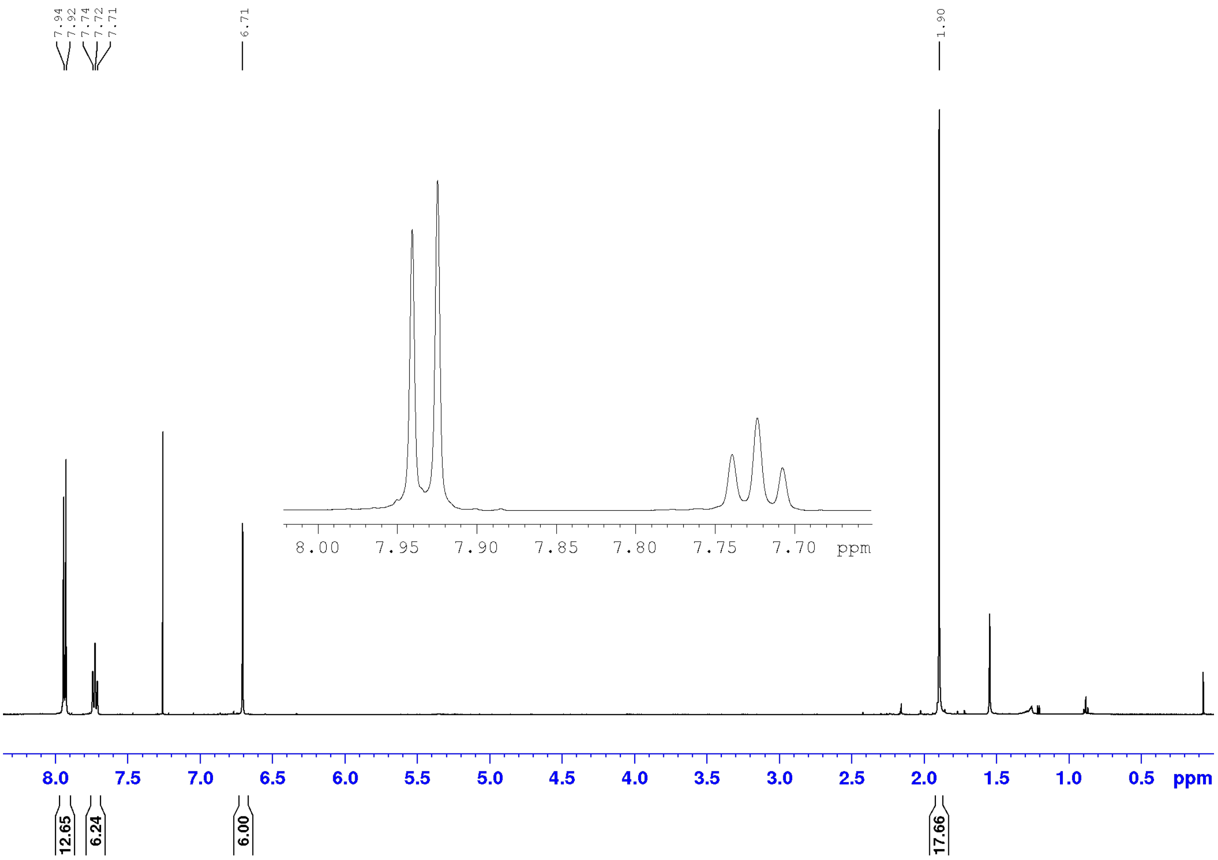

Figure S25: ^{1}H NMR spectrum (500 MHz, 298 K, $CDCl_3$) of **BFG1H**.

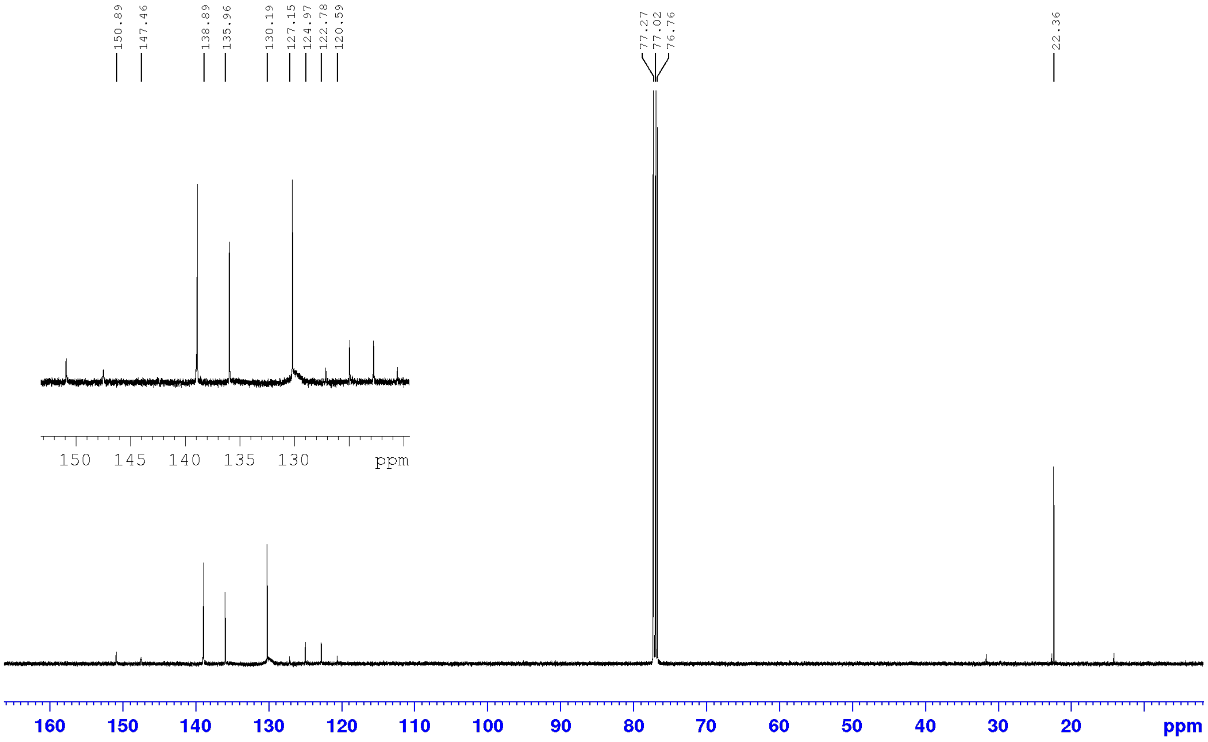

Figure S26: ^{13}C{^{1}H} NMR spectrum (126 MHz, 298 K, $CDCl_3$) of **BFG1H**.

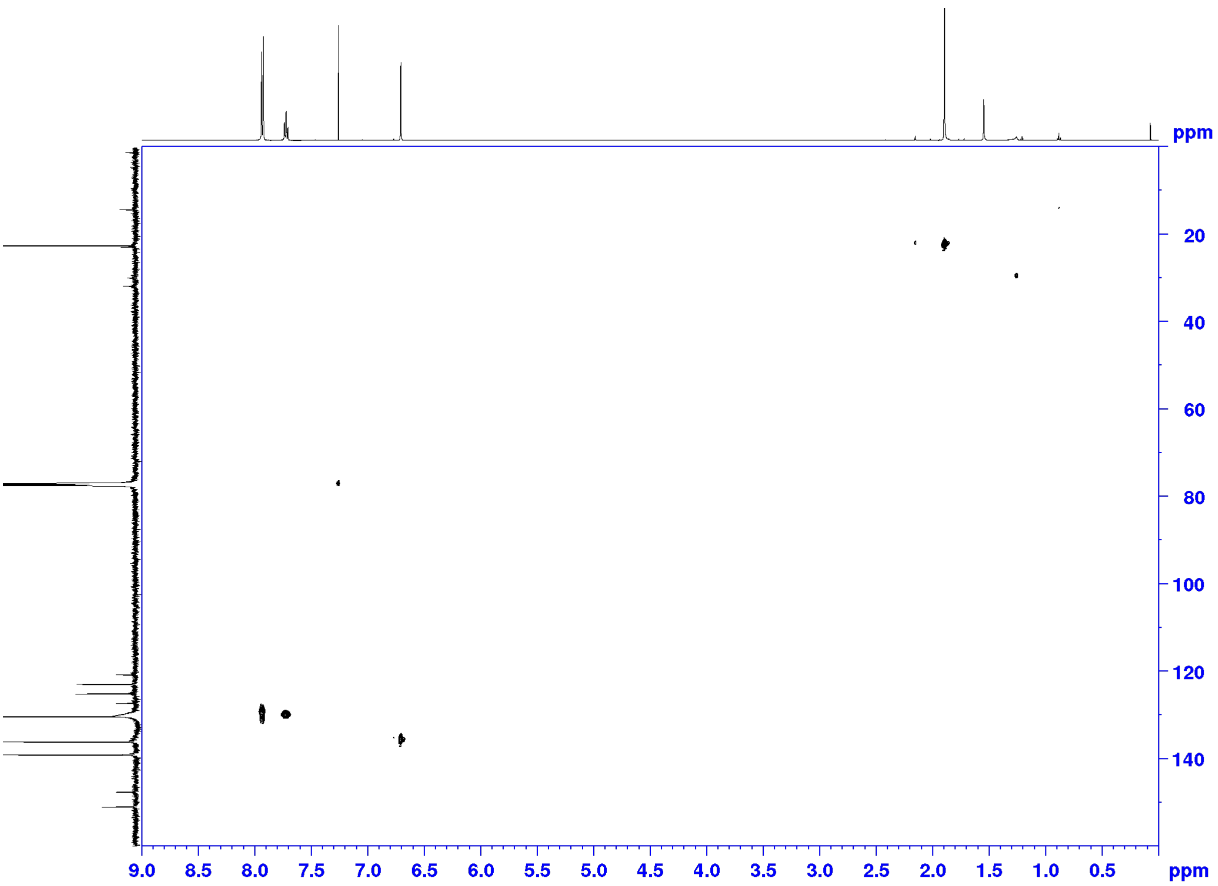

Figure S27: HSQC NMR spectrum ($^{13}C\{^{1}H\}$ 126 MHz, ^{1}H 500 MHz, 298 K, $CDCl_3$) of **BFG1H**.

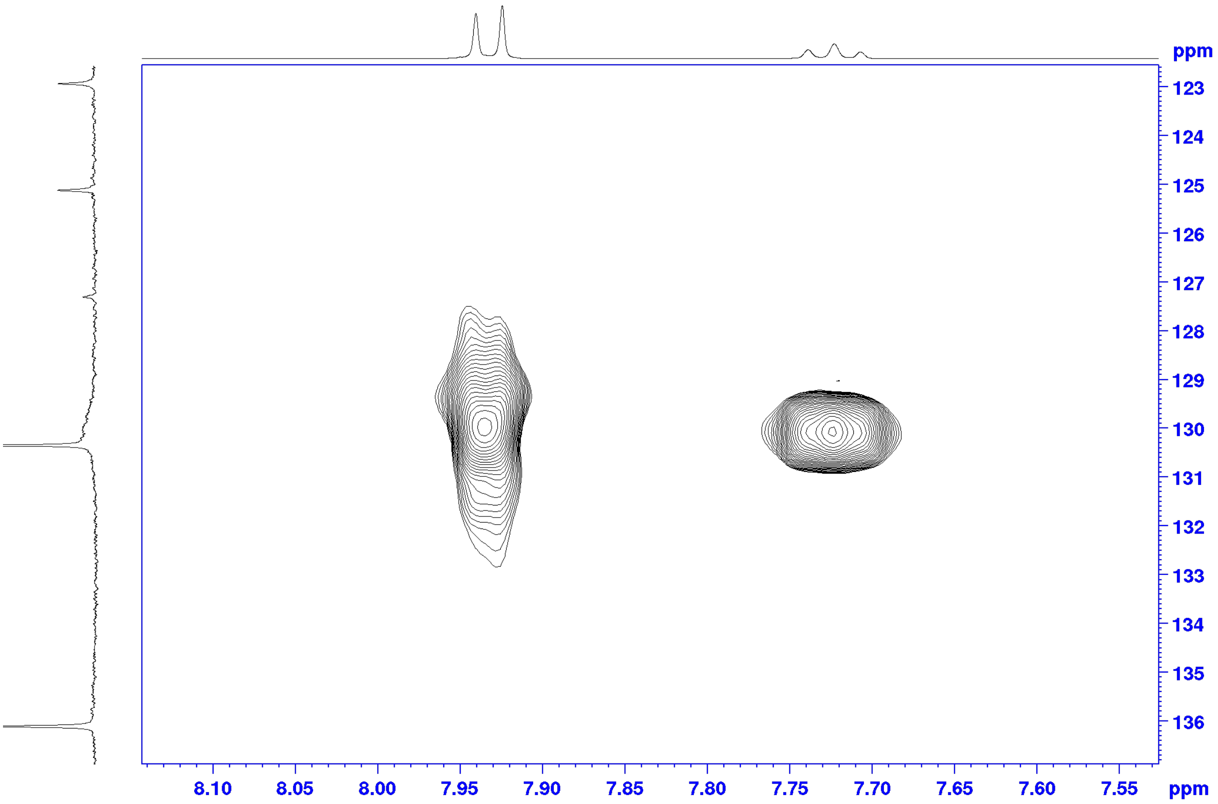

Figure S28: HSQC NMR spectrum ($^{13}C\{^{1}H\}$ 126 MHz, ^{1}H 500 MHz, 298 K, $CDCl_3$) of **BFG1H**.

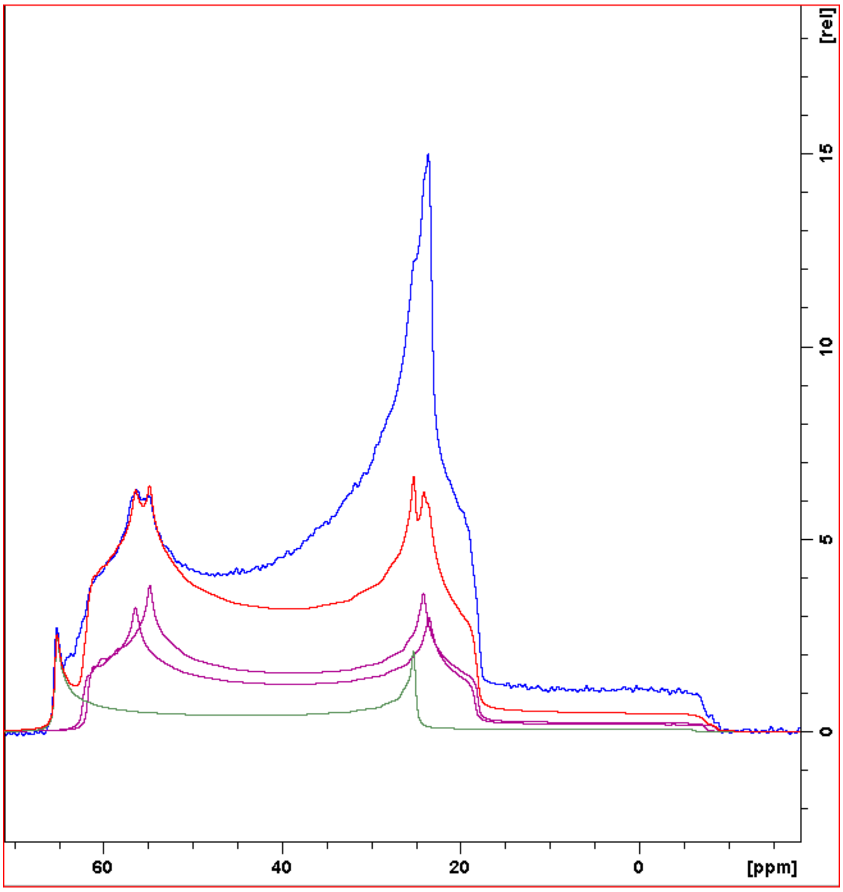

Figure S29: ^{11}B SS NMR spectrum (128.3 MHz, 298 K) of **BFG1H**.

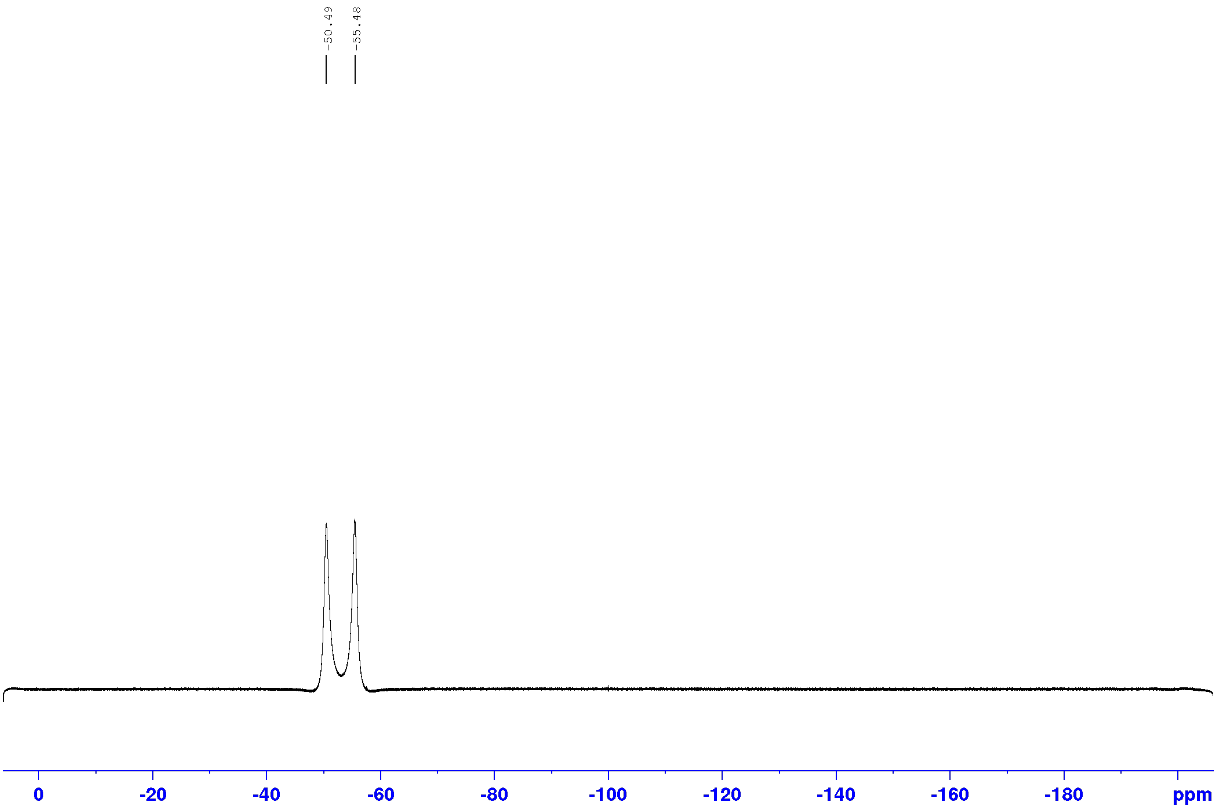

Figure S30: ^{19}F NMR spectrum (471 MHz, 298 K, $CDCl_3$) of **BFG1H**.

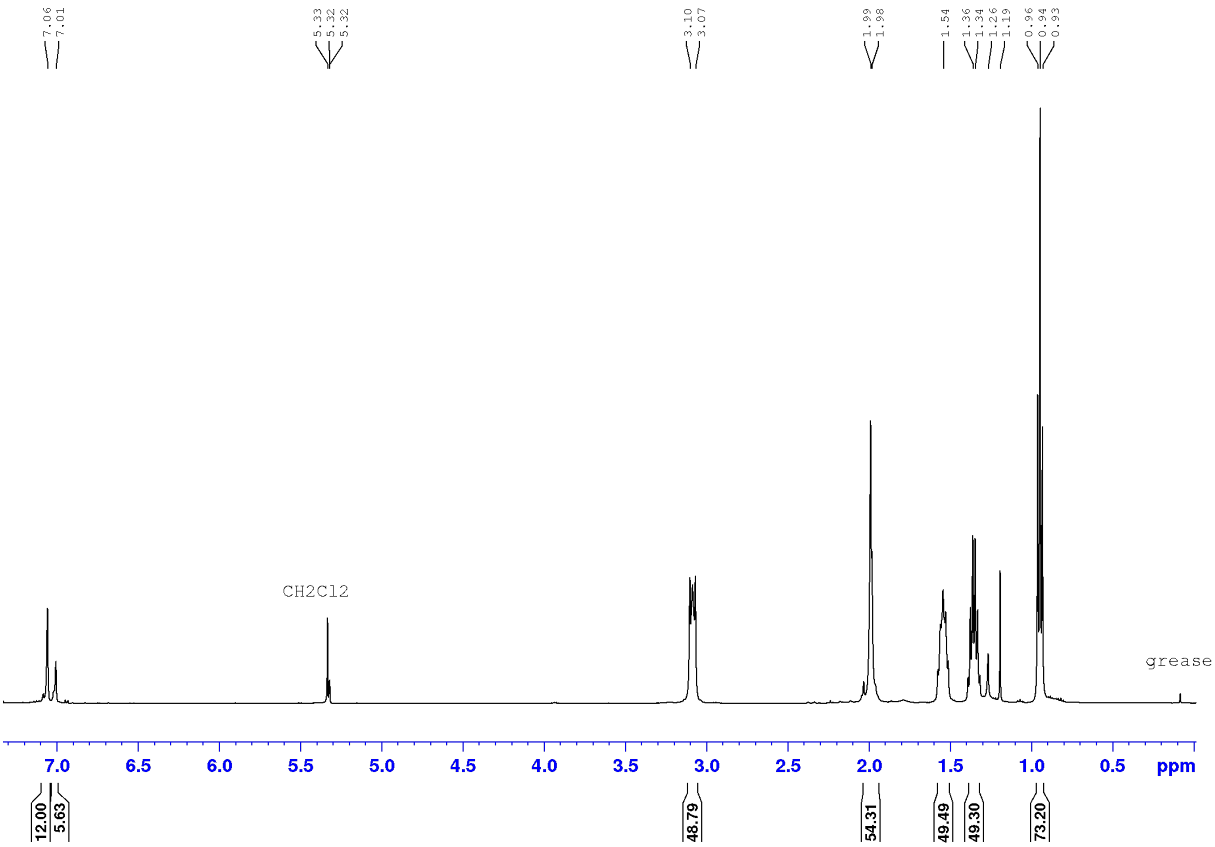

Figure S31: ^{1}H NMR spectrum (500 MHz, 298 K, CD_2Cl_2) of **BG1BF$_3$N(*n*Bu)$_4$**.

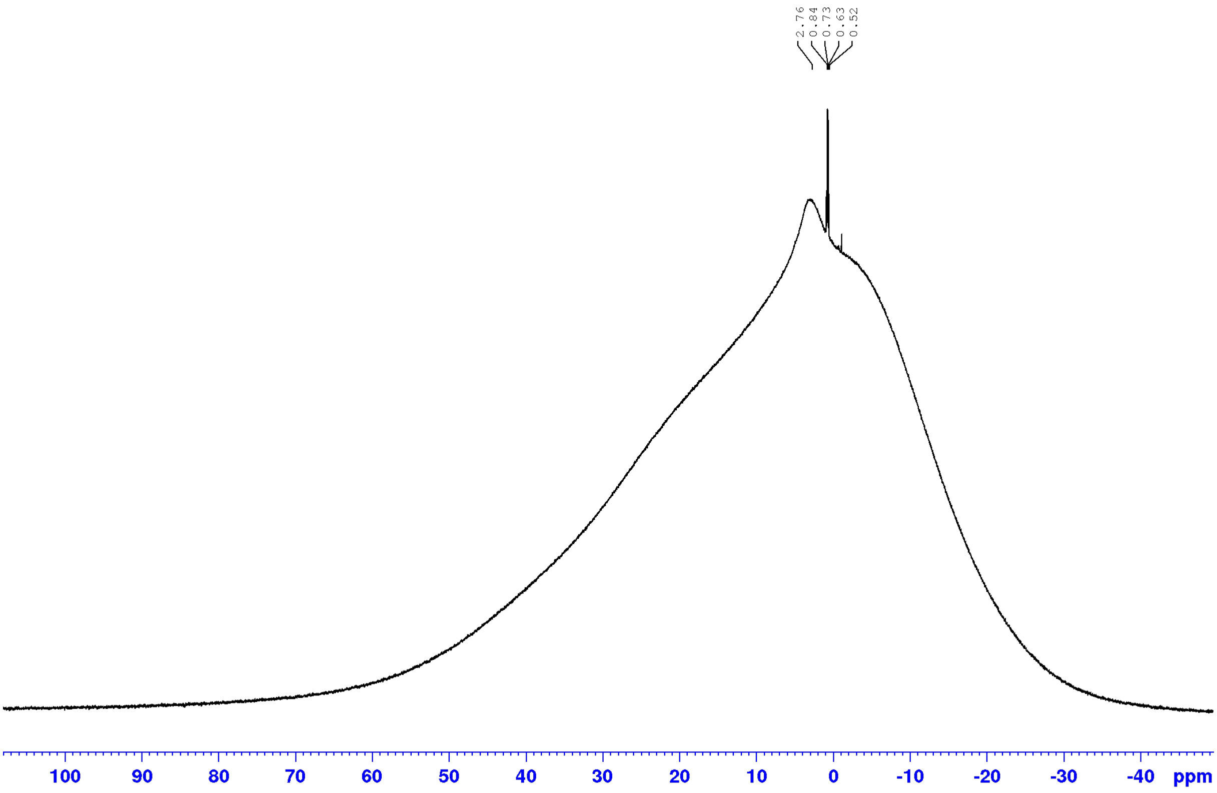

Figure S32: ^{11}B NMR spectrum (128 MHz, 298 K, CD_2Cl_2) of **BG1BF$_3$N(*n*Bu)$_4$**.

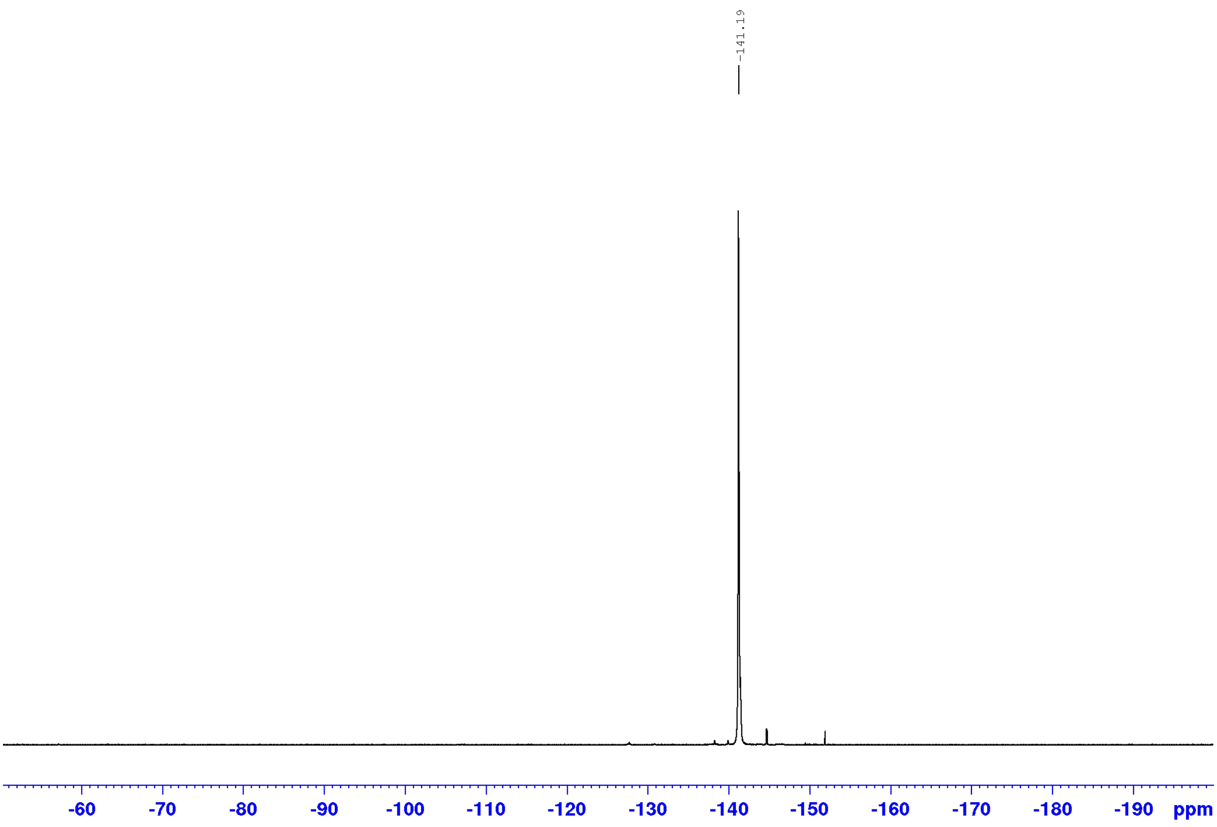

Figure S33: ^{19}F NMR spectrum (471 MHz, 298 K, CD_2Cl_2) of **BG1BF$_3$N(*n*Bu)$_4$**.

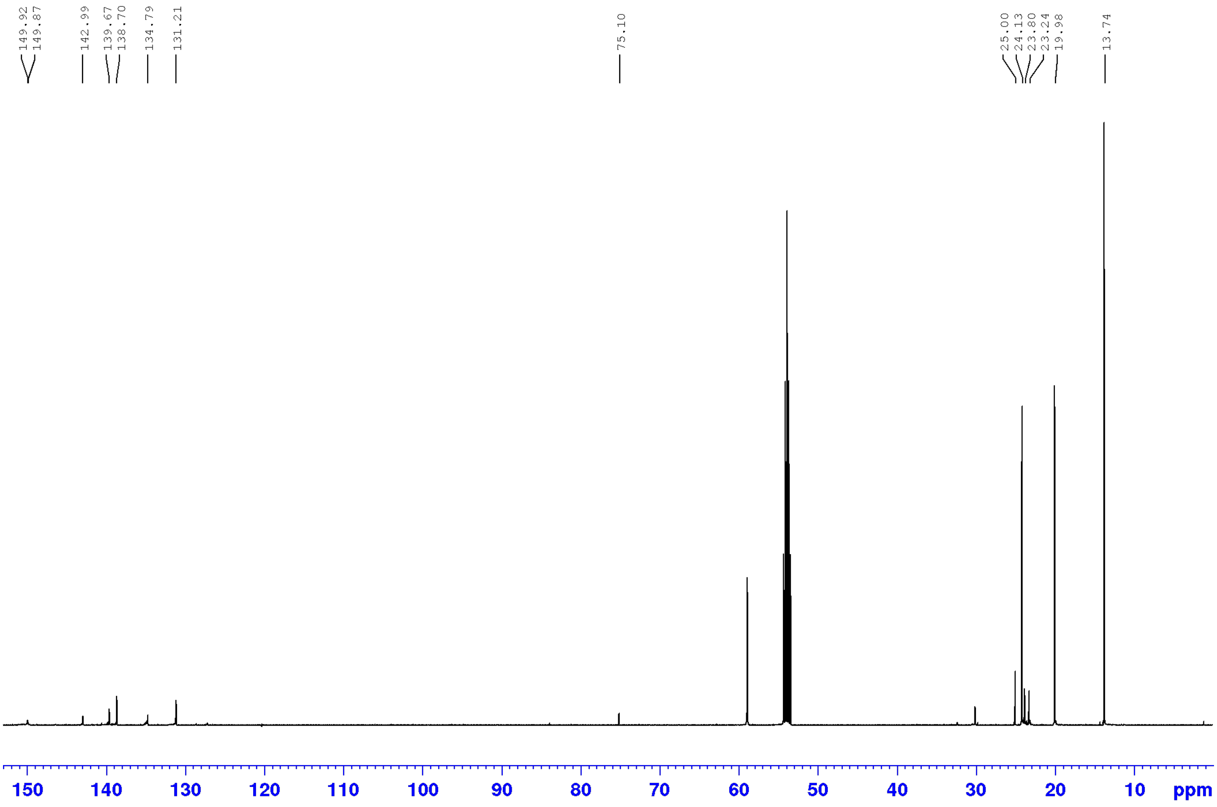

Figure S34: $^{13}C\{^1H\}$ NMR spectrum (126 MHz, 298 K, CD_2Cl_2) of **BG1BF$_3$N(*n*Bu)$_4$**.

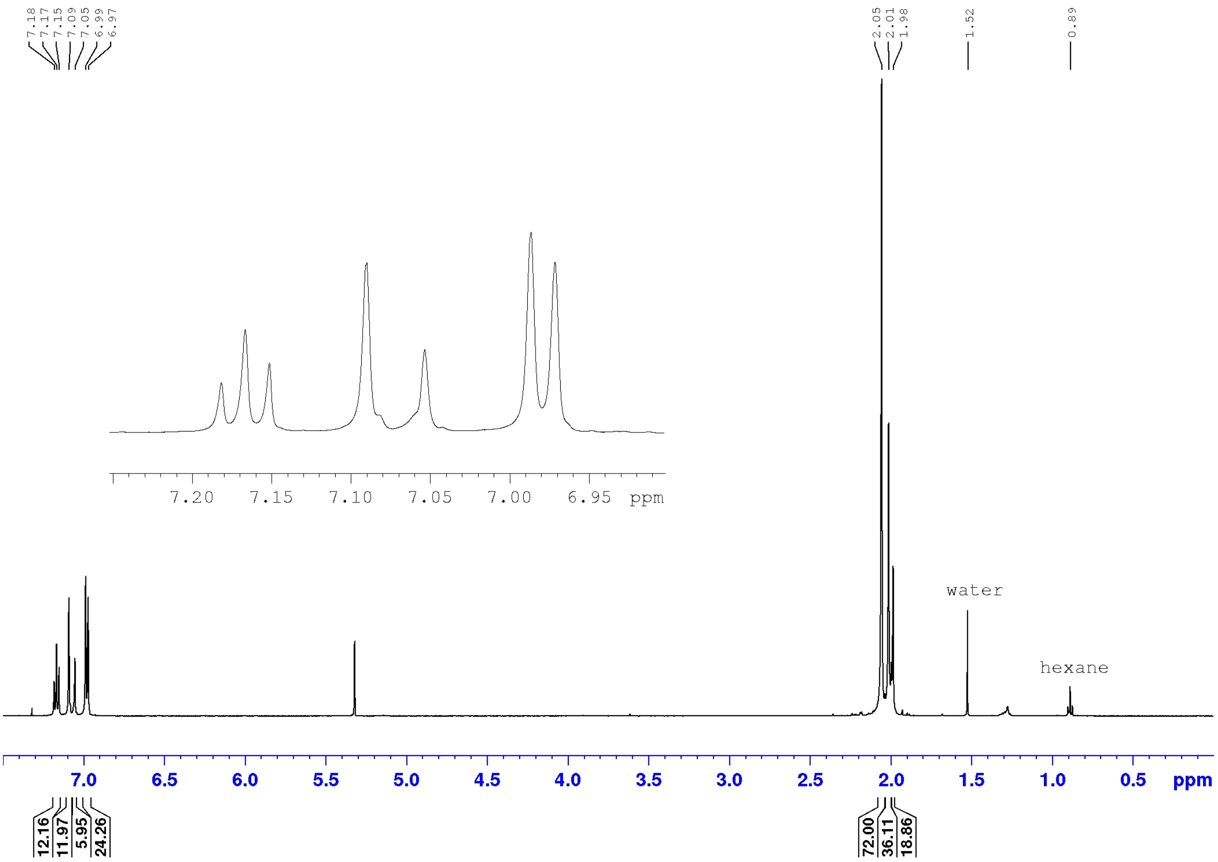

Figure S35: ^{1}H NMR spectrum (500 MHz, 298 K, CD_2Cl_2) of **BG2H**.

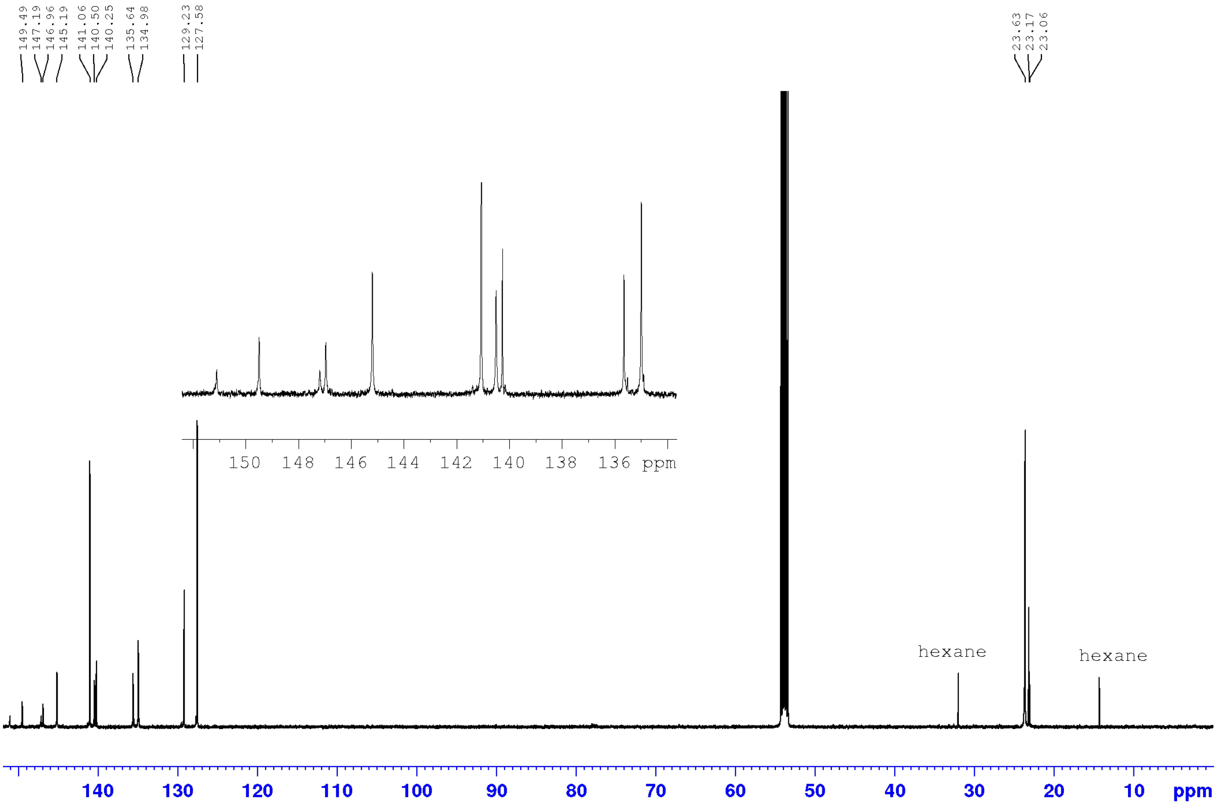

Figure S36: $^{13}C\{^1H\}$ NMR spectrum (126 MHz, 298 K, CD_2Cl_2) of **BG2H**.

Crystal structure determination

Table S1: Single-crystal X-ray diffraction data and structure refinements of **BG1Bpin**, **BG1NMe$_2$**, and **BFG1H**.

Data	**BG1Bpin**	**BG1NMe$_2$**	**BFG1H**
CCDC number	1990178	1990179	1990180
Empirical formula	$C_{108}H_{144}B_{10}O_{12}$	$C_{84}H_{108}B_4N_6$, 1.154(CH_2Cl_2)	$C_{72}H_{42}B_4F_{36}$
Formula weight (g·mol^{-1})	1742.32	1342.95	1634.29
Temperature (K)	100(2)	100(2)	100(2)
Radiation, λ (Å)	Mo-K$_\alpha$ 0.71073	Mo-K$_\alpha$ 0.71073	Mo-K$_\alpha$ 0.71073
Crystal size (mm³)	0.21×0.20×0.15	0.46×0.41×0.12	0.65×0.45×0.37
Crystal color, habit	Colorless block	Yellow plate	Colorless block
Crystal system	Trigonal	Monoclinic	Triclinic
Space group	$P\bar{3}1c$	$P2_1/c$	$P\bar{1}$
a (Å)	22.484(6)	15.1535(15)	8.807(3)
b (Å)	22.484(6)	27.079(3)	16.050(6)
c (Å)	13.358(5)	20.2435(19)	25.018(8)
α (°)	90	90	106.517(10)
β (°)	90	109.323(3)	97.039(11)
γ (°)	120	90	91.027(6)
Volume (Å^3)	5848(4)	7838.9(13)	3359(2)
Z	2	4	2
ρ_{cal} (g·cm^{-3})	0.989	1.138	1.616
μ (mm^{-1})	0.061	0.141	0.163
F(000)	1876	2890	1636
θ range (°)	1.811 – 25.032	1.424 – 24.886	1.774 – 30.999
Reflections collected	33242	43194	148305
Unique reflections	3459	13515	21404
Min. / max. transmission	0.6978/0.7453	0.6762/0.7451	0.7072/0.7463
Parameters / restraints	224 / 18	990 / 126	1241 / 441
GooF on F^2	1.082	1.027	1.009
R1 [I>2σ(I)]	0.0561	0.0902	0.0430
wR^2 (all data)	0.1733	0.2767	0.1080
Max. / min. residual electron density (e·Å^{-3})	0.456 / –0.222	0.598 / –0.929	0.435 / –0.374

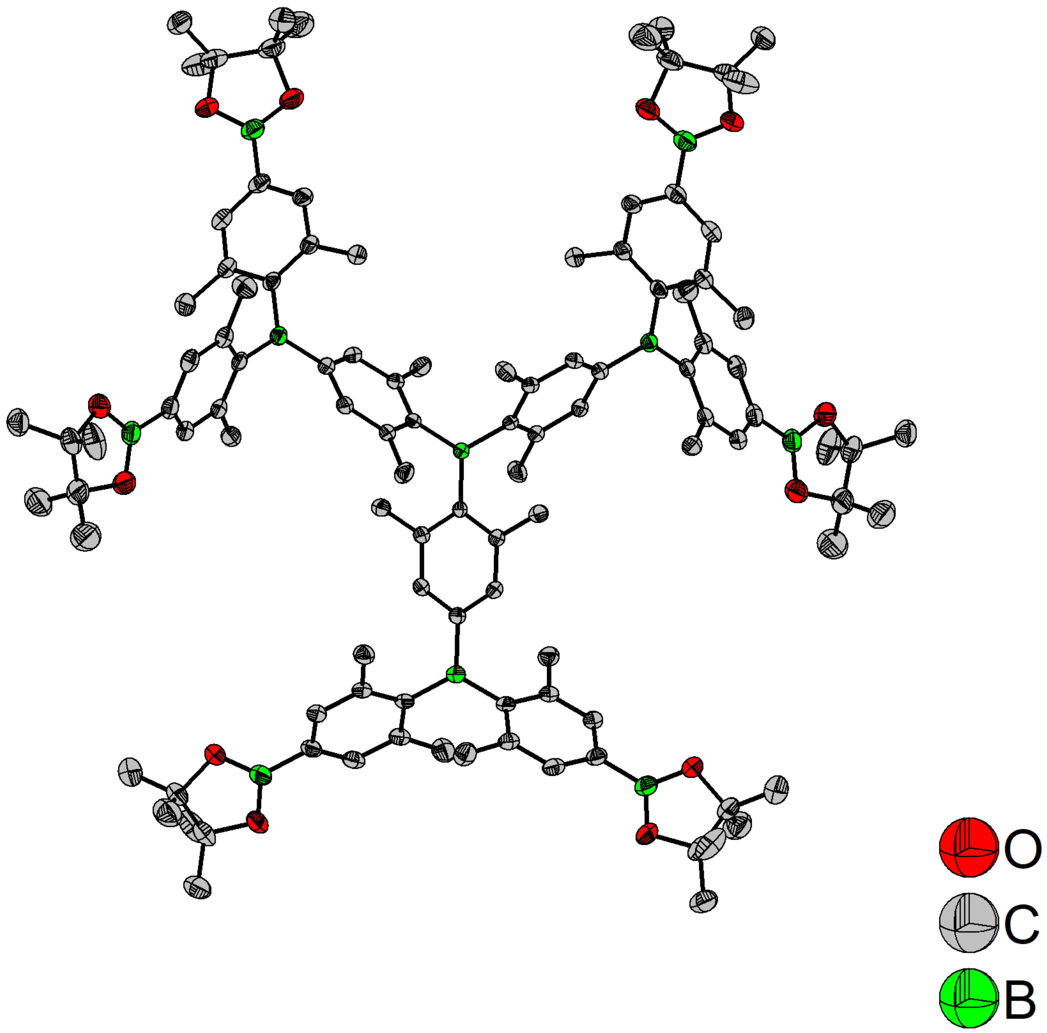

Figure S37: The solid-state molecular structure of **BG1Bpin** determined by single-crystal X-ray diffraction at 100 K. All ellipsoids are drawn at the 50% probability level. H atoms are omitted for clarity. The Bpin moieties are slightly disordered and only the major part (94%) is shown here. The molecule has 3-fold and 2-fold rotational symmetries.

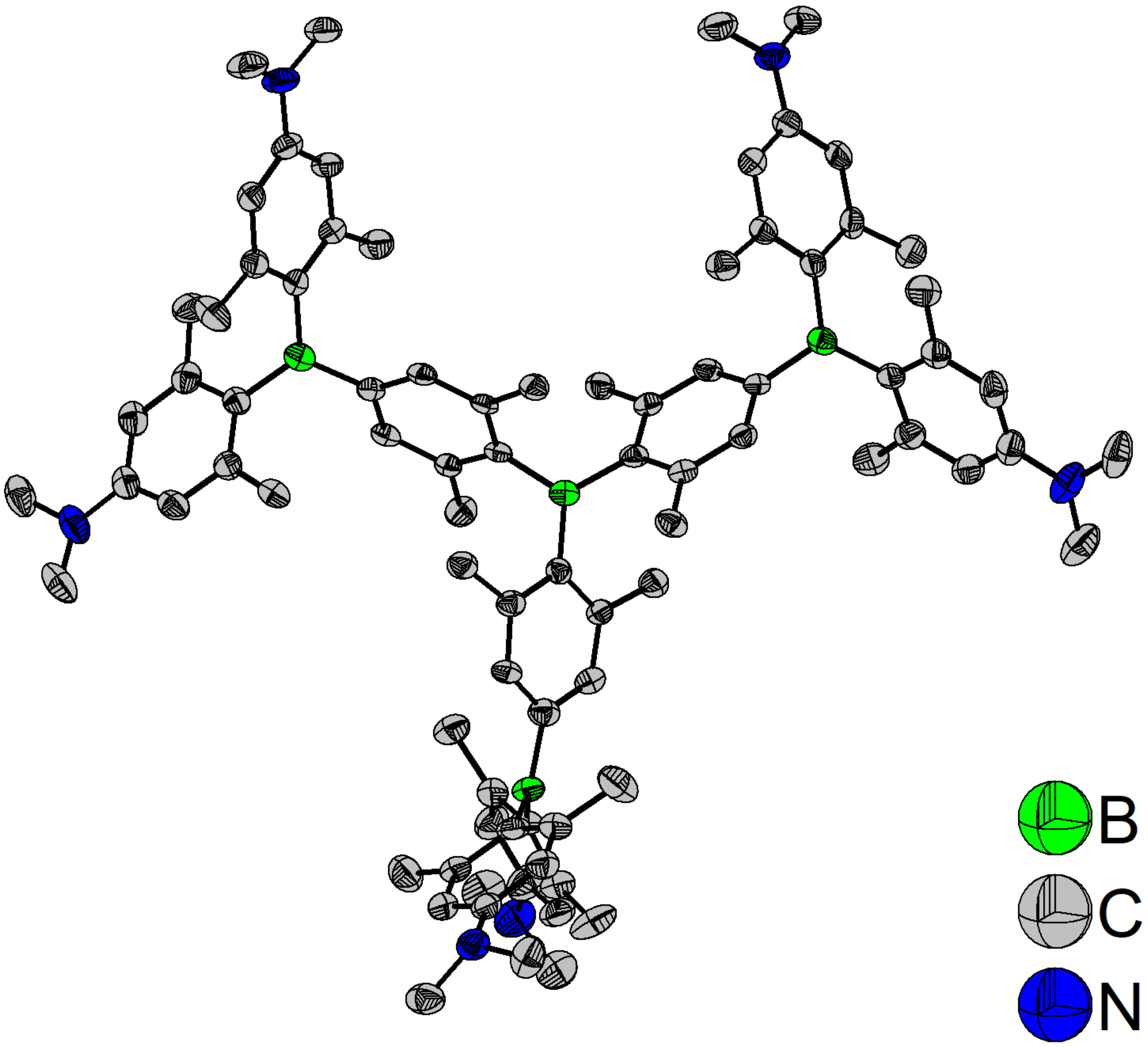

Figure S38: The solid-state molecular structure of **BG1NMe$_2$** determined by single-crystal X-ray diffraction at 100 K. All ellipsoids are drawn at the 50% probability level. H atoms and solvent molecules are omitted for clarity. One of the NMe$_2$ moieties is equally disordered and only one part is shown here.

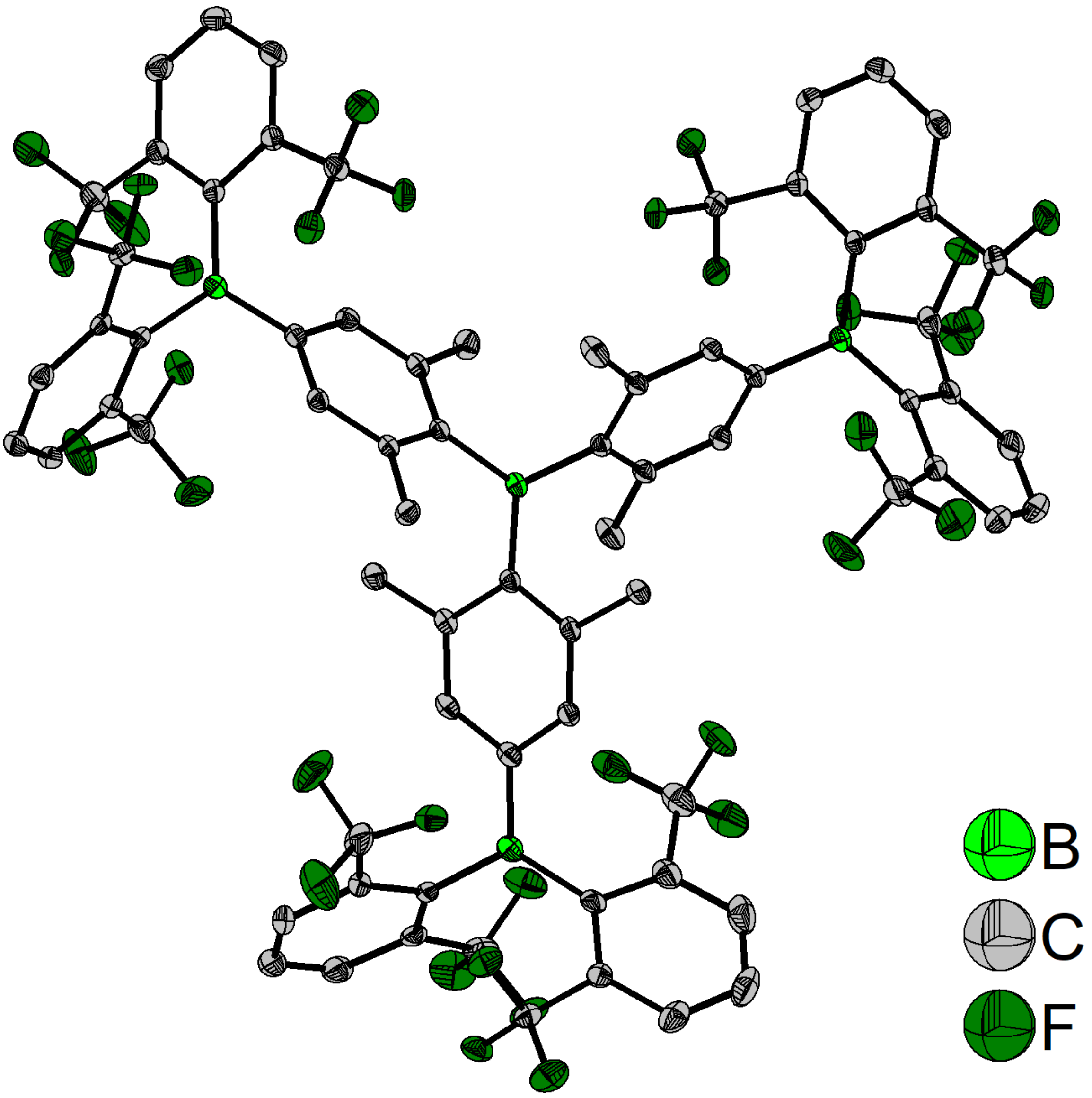

Figure S39: The solid-state molecular structure of **BFG1H** determined by single-crystal X-ray diffraction at 100 K. All ellipsoids are drawn at the 50% probability level. H atoms are omitted for clarity. One of the bis(2,6-bis(trifluoromethyl)phenyl)boranyl (B(^{F}Xyl)$_2$) groups is disordered and only the major part (69%) is shown here.

Photophysical data

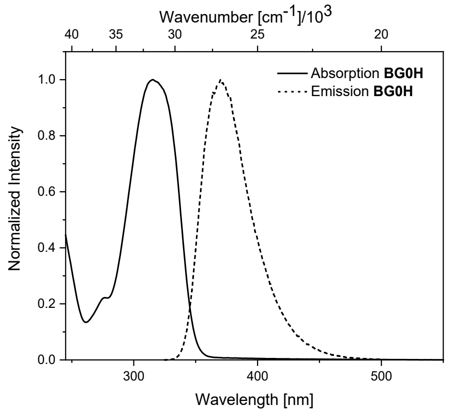

Figure S 40: Absorption and emission spectra of **BG0H** in $CHCl_3$.

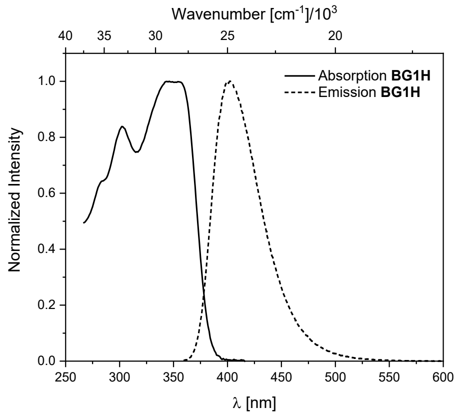

Figure S41: Absorption and emission spectra of **BG1H** in $CHCl_3$.

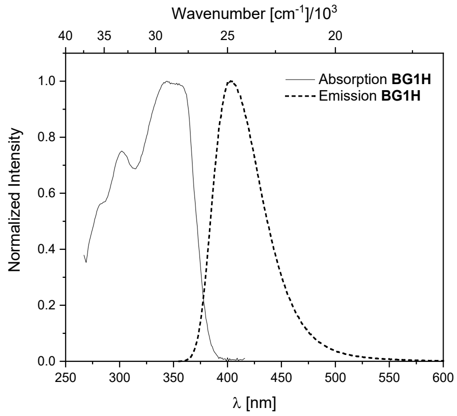

Figure S42: Absorption and emission spectra of **BG1H** in THF.

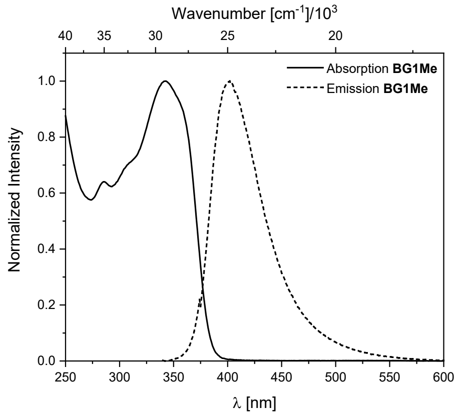

Figure S43: Absorption and emission spectra of **BG1Me** in $CHCl_3$.

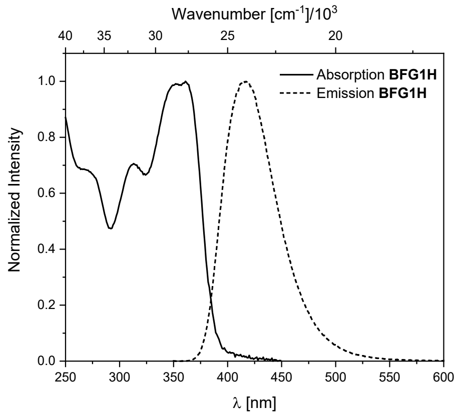

Figure S44: Absorption and emission spectra of **BFG1H** in $CHCl_3$.

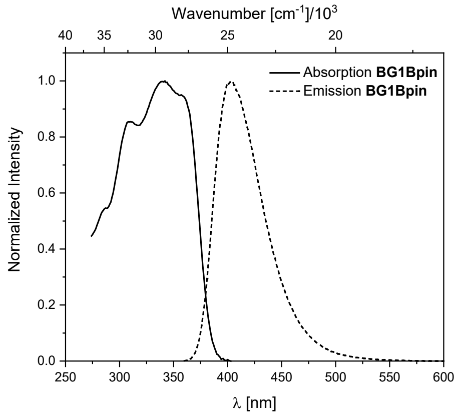

Figure S45: Absorption and emission spectra of **BG1Bpin** in $CHCl_3$.

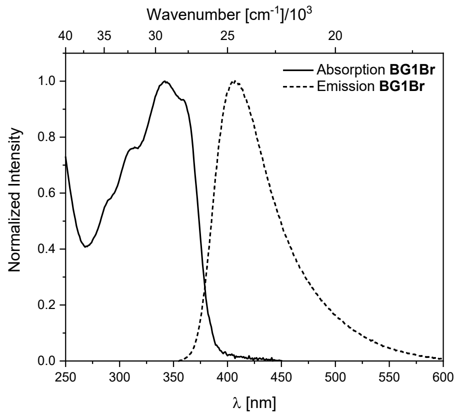

Figure S46: Absorption and emission spectra of **BG1Br** in $CHCl_3$.

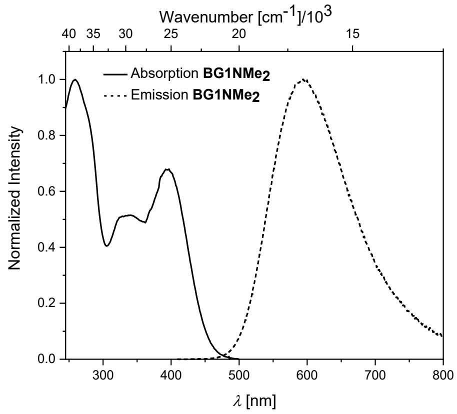

Figure S47: Absorption and emission spectra of **BG1NMe$_2$** in $CHCl_3$.

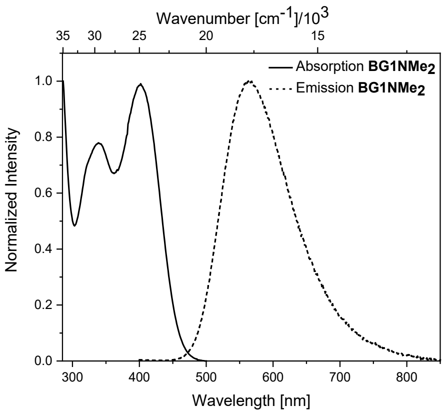

Figure S48: Absorption and emission spectra of **BG1NMe$_2$** in toluene.

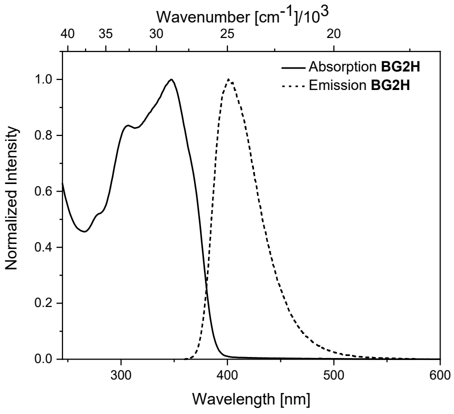

Figure S49: Absorption and emission spectra of **BG2H** in $CHCl_3$.

Electrochemistry

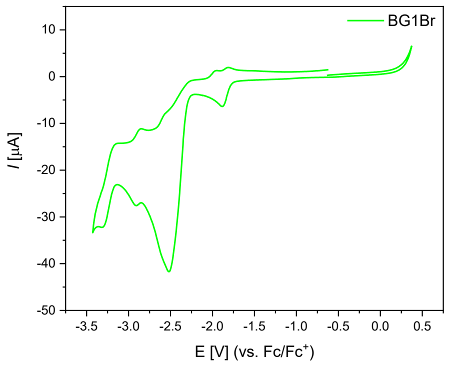

Figure S50: Cyclic voltammogram of **BG1Br** in THF with [nBu$_4$N][PF$_6$] as the electrolyte and a scan rate of 250 mVs^{-1} referenced vs. the Fc/Fc$^+$ redox couple, including irreversible reduction waves.

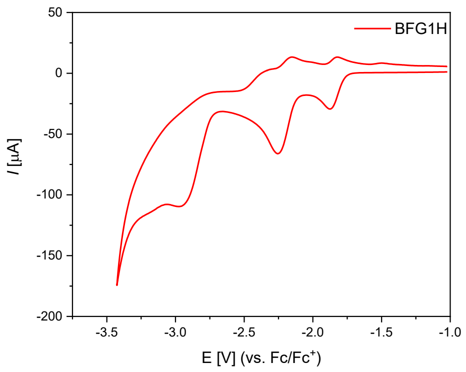

Figure S51: Cyclic voltammogram of **BFG1H** in THF with [nBu$_4$N][PF$_6$] as the electrolyte and a scan rate of 250 mVs^{-1} referenced vs. the Fc/Fc$^+$ redox couple, including irreversible reduction waves.

TD-DFT Calculations

BG1H

Calculated absorption spectrum

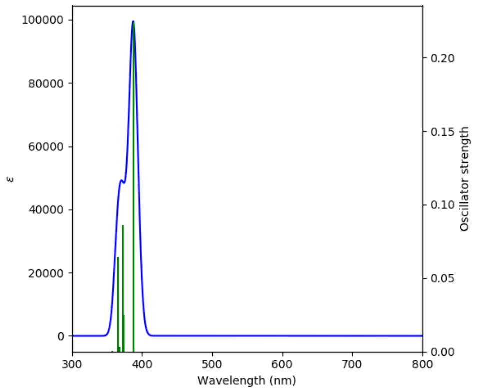

TD-DFT B3LYP/6-31+G(d), gas phase

Orbital	Energy [eV]	Symmetry
L+4	-0.55	E
L+3	-1.26	A
L+2	-2.03	E
L+1	-2.03	E
LUMO	-2.64	A
HOMO	-6.44	E
H-1	-6.44	E
H-2	-6.44	A
H-3	-6.45	E
H-4	-6.45	E

Table S2: Lowest energy singlet electronic transition of **BG1H** (TD-DFT B3LYP/6-31+G(d), gas phase).

State	E [eV]	λ [nm]	*f*	Symmetry	Major contributions	Λ
**1**	3.20	387.54	0.2242	E	H-7->LUMO (43%), H-6->LUMO (20%)	0.49
**2**	3.20	387.54	0.2242	E	H-7->LUMO (20%), H-6->LUMO (43%)	0.49
**3**	3.32	373.22	0.0248	E	H-6->LUMO (16%), H-4->LUMO (62%)	0.42
**4**	3.32	373.22	0.0249	E	H-7->LUMO (16%), H-3->LUMO (62%)	0.41
**5**	3.32	373.08	0.0861	A	H-5->LUMO (83%)	0.36
**6**	3.36	368.67	0.0000	A	H-2->LUMO (88%)	0.25
**7**	3.37	368.30	0.0013	A	H-12->LUMO (71%), H-8->LUMO (25%)	0.48
**8**	3.37	368.26	0.0033	E	HOMO->LUMO (78%)	0.33
**9**	3.37	368.26	0.0033	E	H-1->LUMO (78%)	0.32
**10**	3.39	365.41	0.0645	E	H-14->LUMO (73%), H-9->LUMO (15%)	0.49
**11**	3.39	365.41	0.0645	E	H-13->LUMO (73%), H-10->LUMO (15%)	0.46
**12**	3.47	357.00	0.0003	E	H-14->LUMO (14%), H-9->LUMO (74%)	0.33

Orbitals relevant to the $S_1 \leftarrow S_0$ and $S_2 \leftarrow S_0$ transition

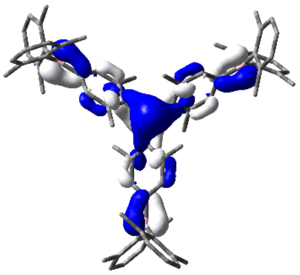

LUMO: –2.637 eV

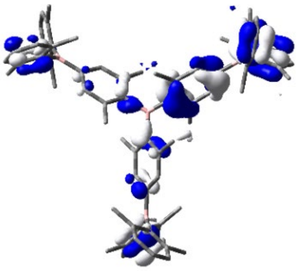

HOMO-6: –6.484 eV

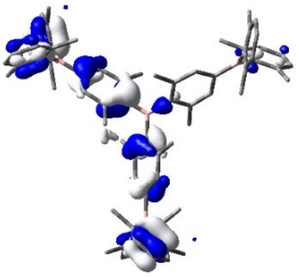

HOMO-7: –6.484 eV

NTOs of the $S_1 \leftarrow S_0$ transition

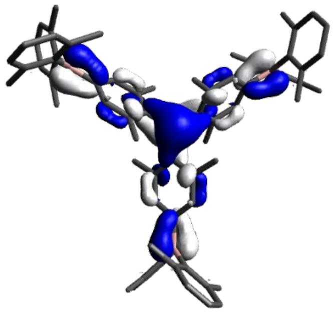

virtual

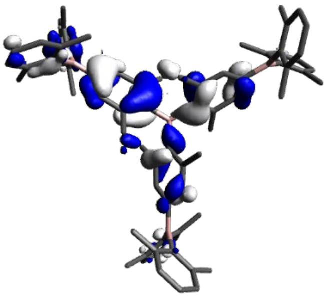

occupied

NTOs of the $S \leftarrow S_0$ transition

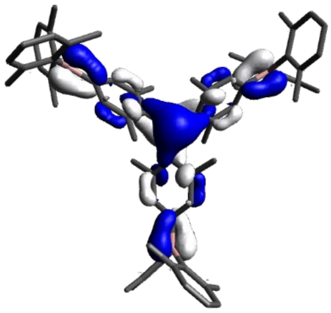

virtual

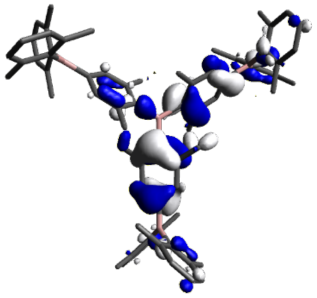

occupied

BFG1H

Calculated absorption spectrum

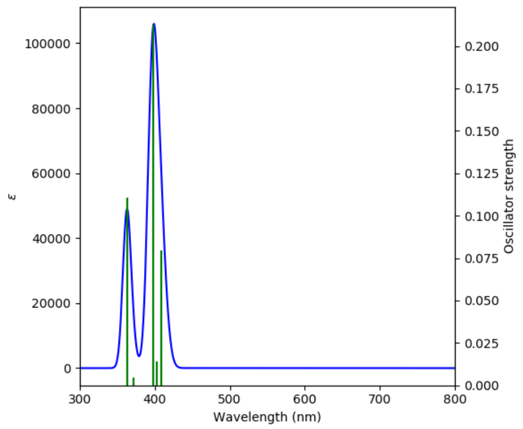

TD-DFT B3LYP/6-31+G(d), gas phase

Orbital	Energy [eV]	Symmetry
L+4	-1.86	A
L+3	-1.96	A
L+2	-2.74	E
L+1	-2.74	E
LUMO	-2.95	A
HOMO	-6.58	E
H-1	-6.58	E
H-2	-6.62	A
H-3	-6.66	E
H-4	-6.66	E

Table S3: Lowest energy singlet electronic transition of BFG1H (TD-DFT B3LYP/6-31+G(d), gas phase).

State	E [eV]	λ [nm]	*f*	Symmetry	Major contributions	Λ
**1**	3.04	408.50	0.0794	E	HOMO->LUMO (89%)	0.50
**2**	3.04	408.50	0.0794	E	H-1->LUMO (89%)	0.51
**3**	3.08	402.38	0.0139	A	H-2->LUMO (81%)	0.41
**4**	3.12	397.39	0.2126	E	H-4->LUMO (85%)	0.51
**5**	3.12	397.39	0.2126	E	H-3->LUMO (85%)	0.52
**6**	3.34	371.35	0.0048	E	H-4->LUMO (11%), H-2->L+2 (29%), H-1->L+1 (23%), HOMO->L+2 (23%)	0.38
**7**	3.34	371.35	0.0048	E	H-3->LUMO (11%), H-2->L+1 (29%), H-1->L+2 (23%), HOMO->L+1 (23%)	0.38
**8**	3.34	371.20	0.0011	A	H-2->LUMO (16%), H-1->L+1 (31%), HOMO->L+2 (31%)	0.38
**9**	3.42	362.99	0.1108	A	H-4->L+2 (27%), H-3->L+1 (27%), H-2->L+2 (14%), H-1->L+1 (12%), HOMO->L+2 (12%)	0.36
**10**	3.42	362.99	0.1108	A	H-4->L+1 (27%), H-3->L+2 (27%), H-2->L+1 (14%), H-1->L+2 (12%), HOMO->L+1 (12%)	0.39
**11**	3.42	362.56	0.0	A	H-1->L+2 (40%), HOMO->L+1 (40%)	0.39
**12**	3.48	356.23	0.0	A	H-4->L+2 (38%), H-3->L+1 (38%), H-1->L+1 (11%), HOMO->L+2 (11%)	0.36

Orbitals relevant to the $S_1 \leftarrow S_0$ transition **Orbitals relevant to the $S_2 \leftarrow S_0$ transition**

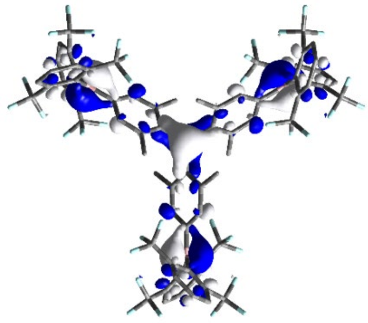

LUMO: –2.953 eV

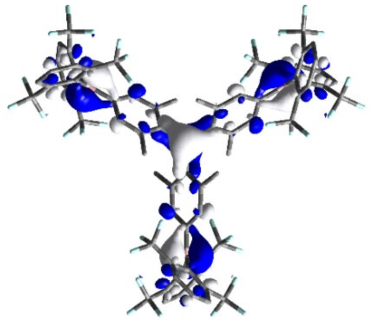

LUMO: –2.953 eV

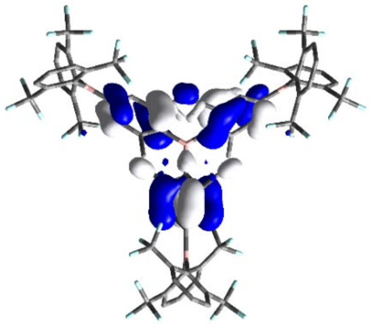

HOMO: –6.577 eV

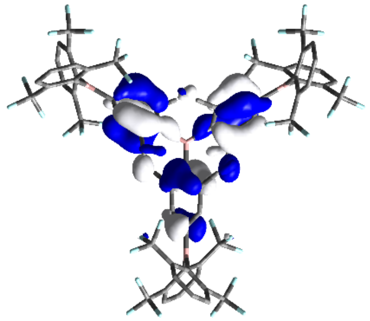

HOMO-1: –6.577 eV

BG1NMe$_2$

Calculated absorption spectrum

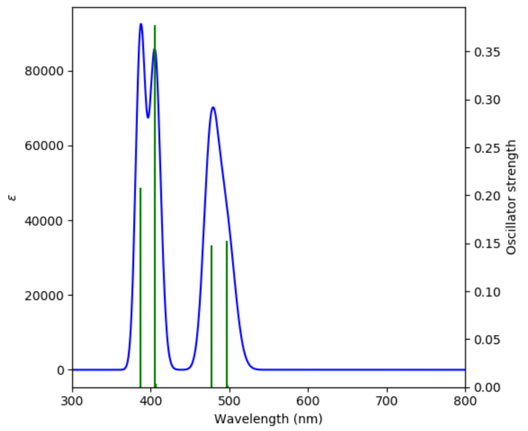

TD-DFT B3LYP/6-31+G(d), gas phase

Orbital	Energy [eV]	Symmetry
L+4	-0.09	A
L+3	-0.66	A
L+2	-1.37	E
L+1	-1.37	E
LUMO	-2.03	A
HOMO	-4.89	E
H-1	-4.89	E
H-2	-4.89	A
H-3	-5.00	E
H-4	-5.00	E

Table S4: Lowest energy singlet electronic transition of **BG1NMe$_2$** (TD-DFT B3LYP/6-31+G(d), gas phase).

State	E [eV]	λ [nm]	*f*	Symmetry	Major contributions	Λ
**1**	2.49	497.95	0.0016	E	H-1->LUMO (96%)	0.25
**2**	2.49	497.95	0.0016	E	HOMO->LUMO (96%)	0.28
**3**	2.49	497.29	0.1525	A	H-2->LUMO (96%)	0.30
**4**	2.60	477.41	0.1482	E	H-4->LUMO (94%)	0.31
**5**	2.60	477.41	0.1482	E	H-3->LUMO (94%)	0.34
**6**	2.65	468.61	0.0000	A	H-5->LUMO (98%)	0.37
**7**	3.05	406.01	0.0034	E	H-2->L+1 (46%), H-1->L+2 (23%), HOMO->L+1 (23%)	0.32
**8**	3.05	406.01	0.0034	E	H-2->L+2 (46%), H-1->L+1 (23%), HOMO->L+2 (23%)	0.43
**9**	3.06	405.47	0.3778	A	H-1->L+1 (46%), HOMO->L+2 (46%)	0.44
**10**	3.20	387.12	0.2080	E	H-5->L+1 (39%), H-4->L+2 (24%), H-3->L+1 (24%)	0.40
**11**	3.20	387.12	0.2080	E	H-5->L+2 (39%), H-4->L+1 (24%), H-3->L+2 (24%)	0.51
**12**	3.23	384.11	0.0000	A	H-4->L+1 (46%), H-3->L+2 (46%)	0.52

Orbitals relevant to the $S_1 \leftarrow S_0$ transition **Orbitals relevant to the $S_2 \leftarrow S_0$ transition**

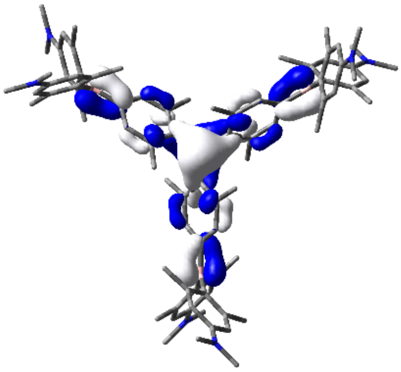

LUMO: –2.028 eV

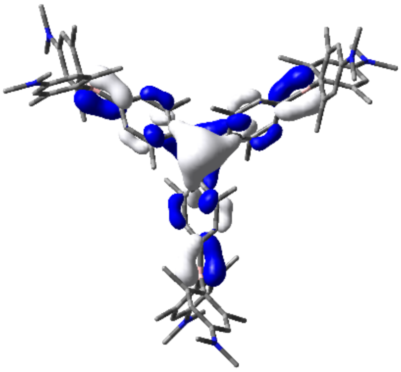

LUMO: –2.028 eV

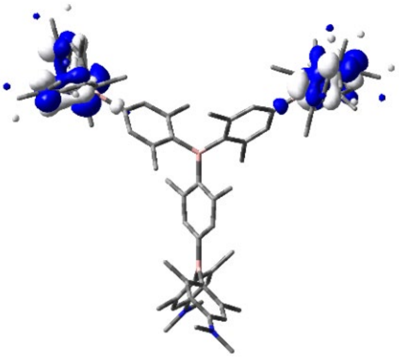

HOMO–1: –4.893 eV

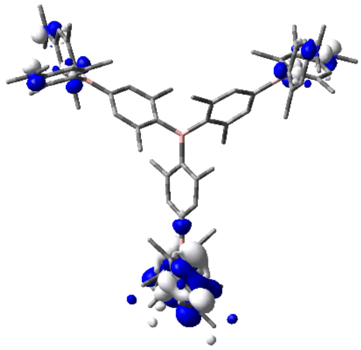

HOMO: –4.893 eV

BG1Br

Calculated absorption spectrum

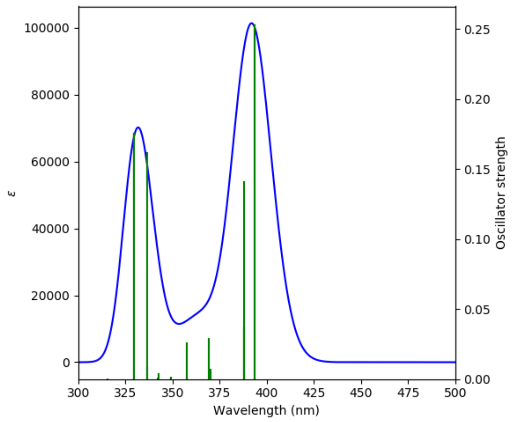

TD-DFT B3LYP/6-31+G(d), gas phase

Orbital	Energy [eV]	Symmetry
L+4	-1.00	E
L+3	-1.70	A
L+2	-2.47	E
L+1	-2.47	E
LUMO	-3.04	A
HOMO	-6.68	E
H-1	-6.68	E
H-2	-6.68	A
H-3	-6.73	E
H-4	-6.73	E

Table S5: Lowest energy singlet electronic transition of BG1Br (TD-DFT CAM-B3LYP/6-31+G(d), gas phase).

State	E [eV]	λ [nm]	*f*	Symmetry	Major contributions	Λ
**1**	3.15	393.71	0.2535	E	H-6->LUMO (11%), H-4->LUMO (13%), H-3->LUMO (53%)	0.46
**2**	3.15	393.71	0.2535	E	H-7->LUMO (11%), H-4->LUMO (53%), H-3->LUMO (13%)	0.45
**3**	3.20	387.95	0.0371	E	H-1->LUMO (13%), HOMO->LUMO (67%)	0.33
**4**	3.20	387.95	0.0371	E	H-1->LUMO (67%), HOMO->LUMO (13%)	0.32
**5**	3.20	387.75	0.1415	A	H-2->LUMO (87%)	0.34
**6**	3.33	372.25	0.0000	A	H-5->LUMO (92%)	0.41
**7**	3.35	370.04	0.0072	A	H-8->LUMO (92%)	0.52
**8**	3.36	369.20	0.0294	E	H-10->LUMO (25%), H-7->LUMO (37%), H-6->LUMO (14%)	0.53
**9**	3.36	369.20	0.0294	E	H-11->LUMO (25%), H-7->LUMO (14%), H-6->LUMO (37%)	0.53
**10**	3.47	357.60	0.0264	E	H-16->LUMO (25%), H-12->LUMO (25%), H-11->LUMO (12%), H-6->LUMO (12%), H-3->LUMO (10%)	0.54
**11**	3.47	357.60	0.0264	E	H-15->LUMO (25%), H-13->LUMO (25%), H-10->LUMO (12%), H-7->LUMO (12%), H-4->LUMO (10%)	0.53
**12**	3.54	350.15	0.0000	A	H-9->LUMO (88%)	0.28
**13**	3.55	348.98	0.0014	E	H-13->LUMO (13%), H-10->LUMO (40%), H-6->LUMO (12%)	0.49
**14**	3.55	348.98	0.0014	E	H-12->LUMO (13%), H-11->LUMO (40%), H-7->LUMO (12%)	0.49
**15**	3.62	342.35	0.0042	E	H-15->LUMO (39%), H-13->LUMO (42%)	0.56

Table S5: continued

State	E [eV]	λ [nm]	*f*	Symmetry	Major contributions	Λ
**16**	3.62	342.35	0.0042	E	H-16->LUMO (39%), H-12->LUMO (42%)	0.57
**17**	3.62	342.18	0.0011	A	H-14->LUMO (84%)	0.38
**18**	3.69	336.46	0.0085	E	H-2->L+1 (26%), H-2->L+2 (15%), H-1->LUMO (12%), H-1->L+1 (18%), HOMO->L+2 (18%)	0.40
**19**	3.69	336.46	0.0085	E	H-2->L+1 (15%), H-2->L+2 (26%), H-1->L+2 (18%), HOMO->LUMO (12%), HOMO->L+1 (18%)	0.41
**20**	3.69	336.36	0.1621	A	H-2->LUMO (13%), H-1->L+1 (21%), H-1->L+2 (21%), HOMO->L+1 (21%), HOMO->L+2 (21%)	0.39
**21**	3.74	331.60	0.0000	A	H-17->LUMO (36%), H-4->L+1 (13%), H-4->L+2 (15%), H-3->L+1 (15%), H-3->L+2 (13%)	0.57
**22**	3.76	329.63	0.1757	A	H-5->L+1 (16%), H-5->L+2 (11%), H-4->L+1 (10%), H-4->L+2 (18%), H-3->L+1 (18%), H-3->L+2 (10%)	0.48
**23**	3.76	329.63	0.1757	A	H-5->L+1 (11%), H-5->L+2 (16%), H-4->L+1 (18%), H-4->L+2 (10%), H-3->L+1 (10%), H-3->L+2 (18%)	0.48
**24**	3.84	323.15	0.0000	A	H-17->LUMO (55%)	0.63
**25**	3.93	315.26	0.0002	A	H-4->L+1 (19%), H-4->L+2 (16%), H-3->L+1 (16%), H-3->L+2 (19%)	0.49

Orbitals relevant to the $S_1 \leftarrow S_0$ and $S_2 \leftarrow S_0$ transition

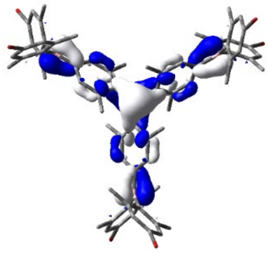

LUMO: –3.036 eV

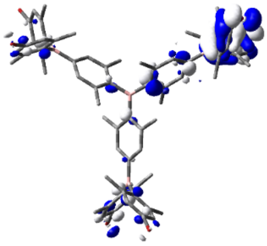

HOMO-3: –6.732 eV

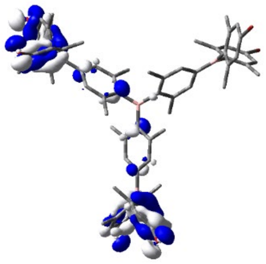

HOMO-4: –6.732 eV

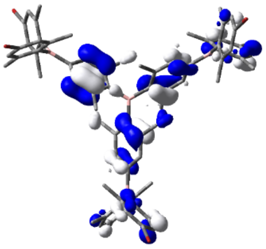

HOMO-6: –6.999 eV

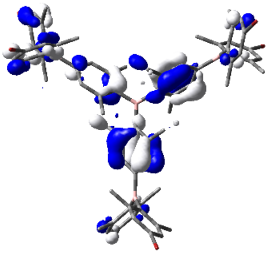

HOMO-7: –6.999 eV

NTOs of the $S_1 \leftarrow S_0$ transition

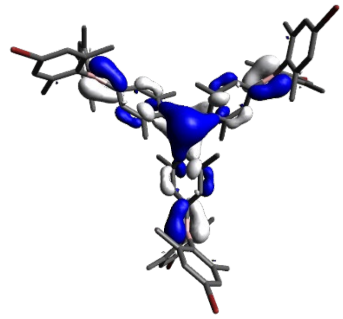

virtual

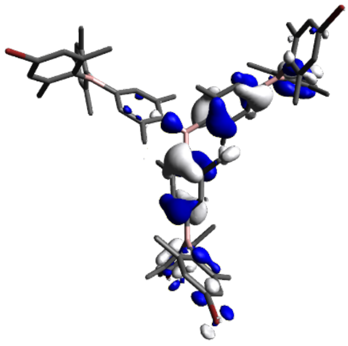

occupied

NTOs of the $S_2 \leftarrow S_0$ transition

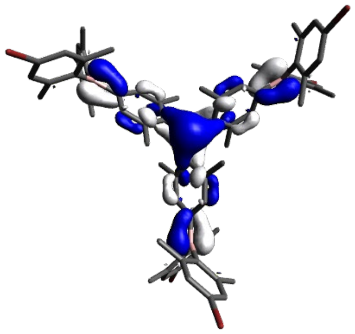

virtual

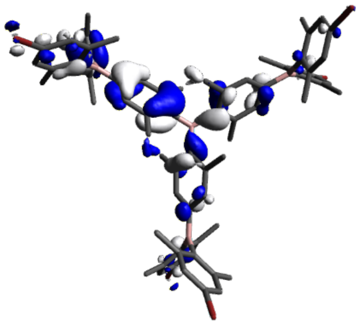

occupied

BG1Me

Calculated absorption spectrum

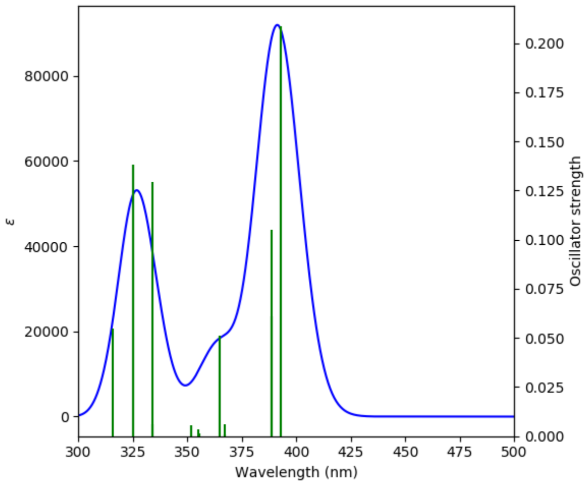

TD-DFT B3LYP/6-31+G(d), gas phase

Orbital	Energy [eV]	Symmetry
L+4	-0.44	E
L+3	-1.14	A
L+2	-1.90	E
L+1	-1.90	E
LUMO	-2.52	A
HOMO	-6.18	E
H-1	-6.18	E
H-2	-6.19	A
H-3	-6.25	E
H-4	-6.25	E

Table S6: Lowest energy singlet electronic transition of **BG1Me** (TD-DFT CAM-B3LYP/6-31+G(d), gas phase).

State	E [eV]	λ [nm]	*f*	Symmetry	Major contributions	Λ
**1**	3.16	392.77	0.2088	E	H-3->LUMO (61%), H-1->LUMO (16%)	0.46
**2**	3.16	392.77	0.2088	E	H-4->LUMO (61%), HOMO->LUMO (16%)	0.44
**3**	3.19	388.91	0.1052	A	H-2->LUMO (87%)	0.36
**4**	3.19	388.88	0.0608	E	H-4->LUMO (14%), HOMO->LUMO (71%)	0.36
**5**	3.19	388.88	0.0608	E	H-3->LUMO (14%), H-1->LUMO (71%)	0.33
**6**	3.33	371.95	0.0000	A	H-5->LUMO (90%)	0.41
**7**	3.38	367.32	0.0059	A	H-14->LUMO (70%), H-9->LUMO (26%)	0.47
**8**	3.38	367.23	0.0011	E	H-13->LUMO (38%), H-7->LUMO (38%)	0.42
**9**	3.38	367.23	0.0011	E	H-12->LUMO (38%), H-6->LUMO (38%)	0.44
**10**	3.40	364.73	0.0512	E	H-15->LUMO (12%), H-13->LUMO (28%), H-11->LUMO (10%), H-7->LUMO (44%)	0.42
**11**	3.40	364.73	0.0512	E	H-16->LUMO (12%), H-12->LUMO (28%), H-10->LUMO (10%), H-6->LUMO (44%)	0.43
**12**	3.42	362.40	0.0	A	H-8->LUMO (91%)	0.34
**13**	3.49	355.68	0.0012	E	H-13->LUMO (17%), H-11->LUMO (72%)	0.35
**14**	3.49	355.68	0.0012	E	H-12->LUMO (17%), H-10->LUMO (72%)	0.33
**15**	3.49	355.18	0.0036	A	H-14->LUMO (26%), H-9->LUMO (65%)	0.39
**16**	3.52	351.86	0.0056	E	H-16->LUMO (75%)	0.63
**17**	3.52	351.86	0.0056	E	H-15->LUMO (75%)	0.60

Table S6: Continued

State	E [eV]	λ [nm]	*f*	Symmetry	Major contributions	Λ
**18**	3.71	334.03	0.006	E	H-2->L+1 (41%), H-1->LUMO (12%), H-1->L+2 (21%), HOMO->L+1 (21%)	0.36
**19**	3.71	334.03	0.006	E	H-2->L+2 (41%), H-1->L+1 (21%), HOMO->LUMO (12%), HOMO->L+2 (21%)	0.47
**20**	3.71	333.96	0.1293	A	H-2->LUMO (12%), H-1->L+1 (41%), HOMO->L+2 (41%)	0.48
**21**	3.77	329.03	0.0	A	H-17->LUMO (46%), H-4->L+1 (23%), H-3->L+2 (23%)	0.67
**22**	3.81	325.26	0.1383	E	H-5->L+2 (29%), H-4->L+1 (27%), H-3->L+2 (27%)	0.56
**23**	3.81	325.26	0.1384	E	H-5->L+1 (29%), H-4->L+2 (27%), H-3->L+1 (27%)	0.43
**24**	3.87	320.75	0.0	A	H-17->LUMO (36%), H-4->L+1 (18%), H-3->L+2 (18%)	0.61
**25**	3.92	315.95	0.0546	E	H-8->L+1 (37%), H-7->L+2 (23%), H-6->L+1 (23%)	0.34

Orbitals relevant to the $S_1 \leftarrow S_0$ and $S_2 \leftarrow S_0$ transition

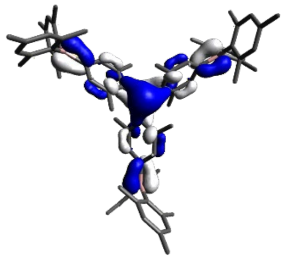

LUMO: –2.520 eV

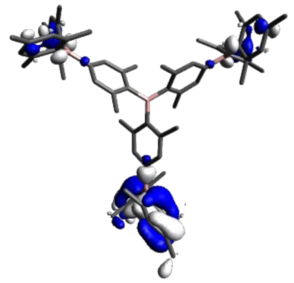

HOMO: –6.183 eV

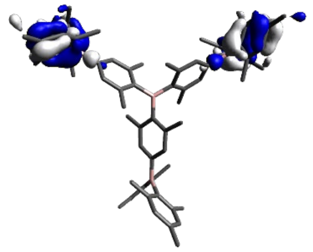

HOMO-1: –6.183 eV

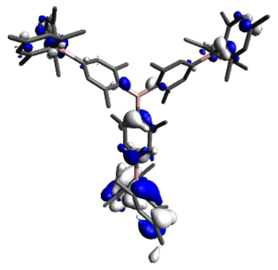

HOMO-3: –6.251 eV

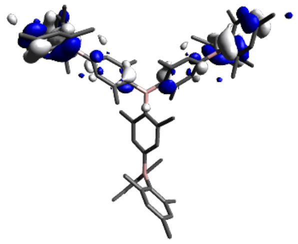

HOMO-4: –6.251 eV

NTOs of the $S_1 \leftarrow S_0$ transition

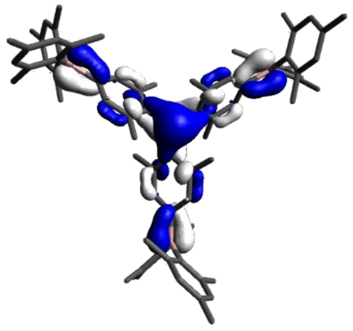

virtual

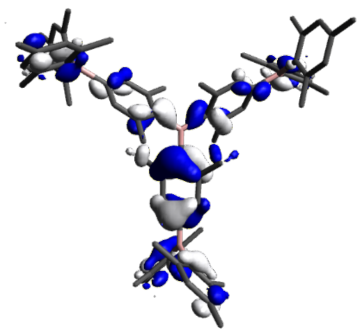

occupied

NTOs of the $S_2 \leftarrow S_0$ transition

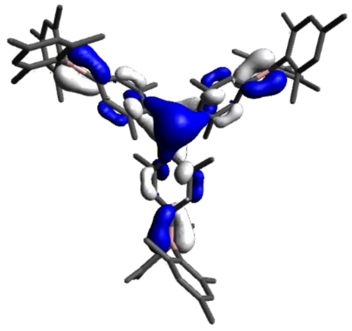

virtual

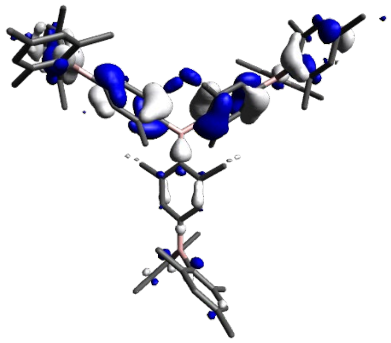

occupied

BG2H

Calculated absorption spectrum

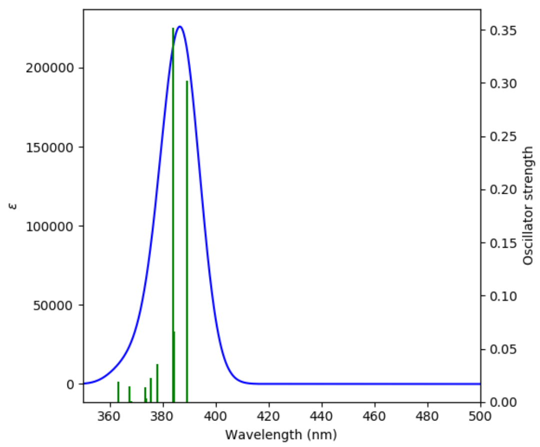

TD-DFT B3LYP/6-31G, gas phase

Orbital	Energy [eV]	Symmetry
L+4	-1.72	E
L+3	-1.78	A2
L+2	-2.21	E
L+1	-2.21	E
LUMO	-2.63	A2
HOMO	-6.21	E
H-1	-6.21	E
H-2	-6.21	E
H-3	-6.21	E
H-4	-6.21	A1

Table S7: Lowest energy singlet electronic transition of **BG2H** (TD-DFT B3LYP/6-31G, gas phase).

State	E [eV]	λ [nm]	*f*	Symmetry	Major contributions	Λ
**1**	3.18	389.41	0.3024	E	H-31->LUMO (33%), H-15->LUMO (37%)	0.43
**2**	3.18	389.41	0.3024	E	H-30->LUMO (33%), H-16->LUMO (37%)	0.41
**3**	3.23	384.23	0.0660	E	H-12->LUMO (53%)	0.31
**4**	3.23	384.23	0.0660	E	H-13->LUMO (53%)	0.28
**5**	3.23	384.01	0.3522	A2	H-14->LUMO (60%), H-4->LUMO (10%)	0.32
**6**	3.27	379.10	0.0000	A1	H-26->LUMO (27%), H-17->LUMO (11%), H-8->LUMO (22%), H-5->LUMO (23%)	0.23
**7**	3.28	378.08	0.0354	E	H-25->LUMO (26%), H-7->LUMO (24%), HOMO->LUMO (16%)	0.21
**8**	3.28	378.08	0.0354	E	H-24->LUMO (26%), H-6->LUMO (24%), H-1->LUMO (16%)	0.19
**9**	3.30	375.48	0.0228	A2	H-9->LUMO (81%)	0.19
**10**	3.30	375.45	0.0228	E	H-11->LUMO (81%)	0.16
**11**	3.30	375.45	0.0228	E	H-10->LUMO (81%)	0.17
**12**	3.31	374.18	0.0000	A1	H-8->LUMO (67%)	0.18
**13**	3.32	373.91	0.0030	E	H-25->LUMO (10%), H-7->LUMO (45%), H-6->LUMO (14%)	0.17
**14**	3.32	373.91	0.0030	E	H-24->LUMO (10%), H-7->LUMO (14%), H-6->LUMO (45%)	0.16
**15**	3.32	373.44	0.0013	E	H-3->LUMO (74%)	0.14
**16**	3.32	373.44	0.0013	E	H-2->LUMO (74%)	0.15
**17**	3.32	373.42	0.0140	A2	H-4->LUMO (82%)	0.16

Table S7: Continued

**State**	**E [eV]**	**λ [nm]**	***f***	**Symmetry**	**Major contributions**	**Λ**
**18**	3.32	373.40	0.0000	A1	H-26->LUMO (17%), H-17->LUMO (12%), H-5->LUMO (60%)	0.19
**19**	3.32	373.40	0.0000	E	H-25->LUMO (16%), HOMO->LUMO (54%)	0.20
**20**	3.32	373.40	0.0000	E	H-24->LUMO (16%), H-1->LUMO (54%)	0.18
**21**	3.37	368.11	0.0003	A2	H-36->LUMO (15%), H-27->LUMO (46%), H-20->LUMO (27%)	0.34
**22**	3.37	367.37	0.0147	E	H-38->LUMO (10%), H-28->LUMO (36%), H-21->LUMO (35%)	0.29
**23**	3.37	367.37	0.0145	E	H-37->LUMO (10%), H-29->LUMO (37%), H-22->LUMO (35%)	0.30
**24**	3.38	367.27	0.0000	A1	H-26->LUMO (14%), H-23->LUMO (68%)	0.31
**25**	3.41	363.27	0.0191	E	H-38->LUMO (34%), H-31->LUMO (11%), H-21->LUMO (27%), H-19->LUMO (10%)	0.35

BG2H

Calculated absorption spectrum

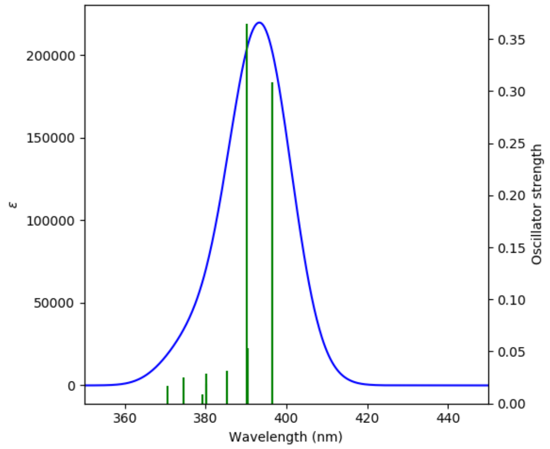

TD-DFT B3LYP/6-31+G(d), gas phase

Orbital	Energy [eV]
L+4	-2.02
L+3	-2.07
L+2	-2.49
L+1	-2.49
LUMO	-2.89
HOMO	-6.43
H-1	-6.43
H-2	-6.43
H-3	-6.43
H-4	-6.43

Table S8: Lowest energy singlet electronic transition of **BG2H** (TD-DFT B3LYP/6-31G, gas phase).

State	E [eV]	λ [nm]	*f*	Major contributions	Λ
**1**	3.13	396.44	0.3082	H-30->LUMO (36%), H-16->LUMO (36%)	0.44
**2**	3.13	396.44	0.3082	H-31->LUMO (36%), H-15->LUMO (36%)	0.45
**3**	3.18	390.43	0.0532	H-12->LUMO (60%)	0.33
**4**	3.18	390.43	0.0531	H-13->LUMO (60%)	0.29
**5**	3.18	390.10	0.3649	H-14->LUMO (63%)	0.34
**6**	3.21	386.14	0.0000	H-26->LUMO (36%), H-17->LUMO (17%), H-8->LUMO (24%)	0.28
**7**	3.22	385.27	0.0316	H-24->LUMO (48%), H-7->LUMO (23%)	0.25
**8**	3.22	385.27	0.0316	H-25->LUMO (48%), H-6->LUMO (23%)	0.29
**9**	3.26	380.21	0.0185	H-9->LUMO (78%)	0.20
**10**	3.26	380.14	0.0288	H-10->LUMO (80%)	0.19
**11**	3.26	380.14	0.0288	H-11->LUMO (80%)	0.16
**12**	3.27	379.55	0.0000	H-8->LUMO (25%), H-5->LUMO (61%)	0.14
**13**	3.27	379.48	0.0002	H-7->LUMO (25%), H-4->LUMO (60%)	0.12
**14**	3.27	379.48	0.0002	H-6->LUMO (25%), H-3->LUMO (61%)	0.13
**15**	3.27	379.26	0.0010	H-2->LUMO (87%)	0.11
**16**	3.27	379.26	0.0010	H-1->LUMO (87%)	0.13
**17**	3.27	379.25	0.0089	HOMO->LUMO (87%)	0.14

Table S8: Continued

State	E [eV]	λ [nm]	*f*	Major contributions	Λ
**18**	3.27	379.12	0.0000	H-26->LUMO (12%), H-17->LUMO (11%), H-8->LUMO (35%), H-5->LUMO (27%)	0.20
**19**	3.27	379.11	0.0001	H-25->LUMO (12%), H-6->LUMO (34%), H-3->LUMO (29%)	0.20
**20**	3.27	379.11	0.0001	H-24->LUMO (12%), H-7->LUMO (34%), H-4->LUMO (29%)	0.17
**21**	3.30	375.66	0.0001	H-35->LUMO (27%), H-27->LUMO (46%), H-20->LUMO (14%)	0.39
**22**	3.31	374.64	0.0247	H-38->LUMO (20%), H-28->LUMO (44%), H-21->LUMO (19%)	0.36
**23**	3.31	374.64	0.0246	H-37->LUMO (20%), H-29->LUMO (44%), H-22->LUMO (19%)	0.33
**24**	3.32	373.72	0.0000	H-26->LUMO (16%), H-23->LUMO (61%)	0.33
**25**	3.35	370.53	0.0171	H-38->LUMO (39%), H-30->LUMO (13%), H-21->LUMO (23%)	0.40

Orbitals relevant to the $S_1 \leftarrow S_0$ and $S_2 \leftarrow S_0$ transition

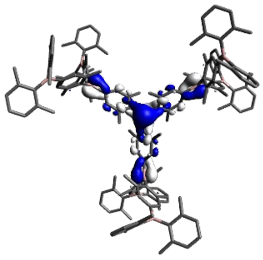

LUMO: –2.629 eV

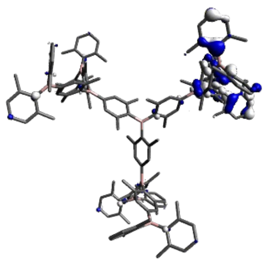

HOMO-15: –6.302 eV

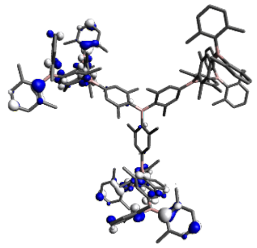

HOMO-16: –6.302 eV

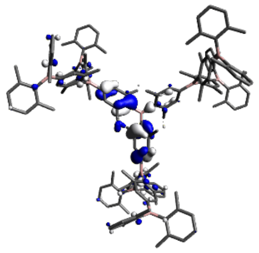

HOMO-30: –6.542 eV

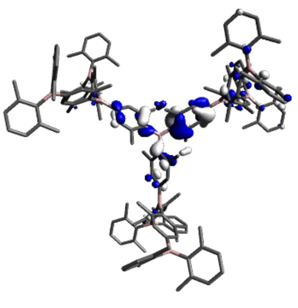

HOMO-31: –6.542 eV

NTOs of the $S_1 \leftarrow S_0$ transition

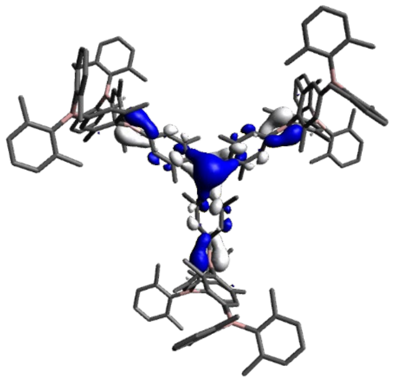

virtual

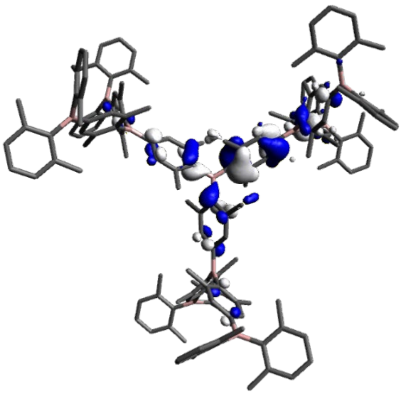

occupied

NTOs of the $S_2 \leftarrow S_0$ transition

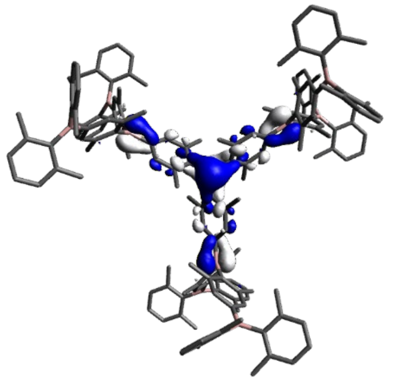

virtual

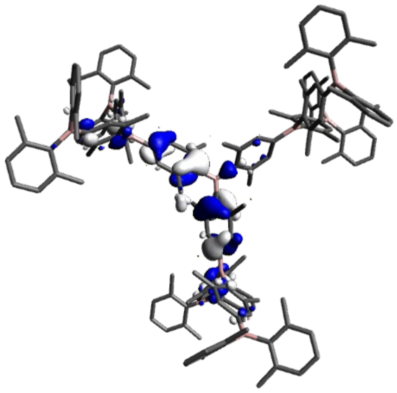

occupied

Theoretical calculations: Cartesian coordinates

BG1H

DFT B3LYP/6-31G, gas phase, S_0

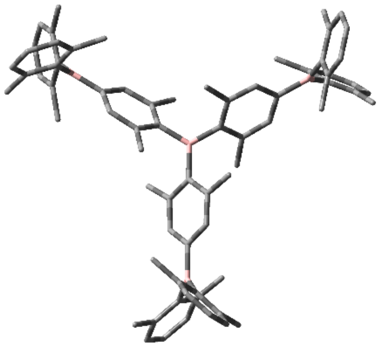

Point group: C_3

Total energy: −1,813,590.94 kcal mol^{-1}

Dipole moment: 0.01 D

Immaginary frequencies: 0

C	-0.00021900	1.58674700	-0.00212200
C	-1.37405400	-0.79356300	-0.00212200
C	0.77748200	2.32342000	-0.94761800
C	0.73860800	3.72424700	-0.94648600
C	0.00011900	4.46410600	-0.00051800
C	-0.73859800	3.72335700	0.94456900
C	-0.77790100	2.32254500	0.94411400
C	-1.62243300	-1.83495500	0.94411400
C	-2.85522300	-2.50132400	0.94456900
C	-3.86608800	-2.23195000	-0.00051800
C	-3.59459700	-1.22247000	-0.94648600
C	-2.40088200	-0.48839000	-0.94761800
C	-1.64083300	1.64562200	1.99537500
C	-0.60473400	-2.24381400	1.99537500
C	-2.24666600	0.59672400	-1.99960200
C	1.64011100	1.64730700	-1.99960200
B	0.00000000	0.00000000	-0.00238600
C	1.37427300	-0.79318400	-0.00212200
C	1.62339900	-1.83502900	-0.94761800
C	2.85598900	-2.50177700	-0.94648600
C	3.86597000	-2.23215600	-0.00051800
C	3.59382100	-1.22203400	0.94456900
C	2.40033400	-0.48759100	0.94411400
C	2.24556700	0.59819200	1.99537500
C	0.60655400	-2.24403200	-1.99960200
B	0.00000000	6.03373200	0.00030300
B	5.22536500	-3.01686600	0.00030300
B	-5.22536500	-3.01686600	0.00030300
C	-6.02719000	-3.15645800	-1.36060600
C	-5.74563800	-3.64100400	1.36197100
C	-0.28038300	6.79637100	1.36197100
C	0.28002200	6.79792900	-1.36060600
C	6.02602100	-3.15536700	1.36197100
C	5.74716800	-3.64147000	-1.36060600
C	-6.03352400	-5.03433400	1.47417900
C	-6.47138400	-5.56671300	2.69741300
C	-6.65725500	-4.75029900	3.81321300
C	-6.39410600	-3.38409700	3.71452300
C	-5.92932800	-2.82018500	2.51534100
C	-7.37829300	-2.71020900	-1.47196900
C	-8.05889400	-2.82375700	-2.69478300
C	-7.44501000	-3.39238200	-3.81100900
C	-6.12992700	-3.84666400	-3.71309100
C	-5.40855800	-3.72560200	-2.51430500
C	1.34203700	7.74489300	-1.47196900
C	1.58400100	8.39108600	-2.69478300
C	0.78461600	8.14375900	-3.81100900
C	-0.26634500	7.23200500	-3.71309100
C	-0.52218700	6.54675000	-2.51430500
C	-1.34309900	7.74235200	1.47417900
C	-1.58522300	8.38774000	2.69741300
C	-0.78525200	8.14050200	3.81321300
C	0.26633900	7.22950600	3.71452300
C	0.52231200	6.54504100	2.51534100
C	7.37662300	-2.70801800	1.47417900
C	8.05660700	-2.82102600	2.69741300
C	7.44250700	-3.39020300	3.81321300
C	6.12776700	-3.84541000	3.71452300
C	5.40701600	-3.72485600	2.51534100
C	6.03625600	-5.03468500	-1.47196900
C	6.47489300	-5.56732800	-2.69478300
C	6.66039400	-4.75137700	-3.81100900
C	6.39627200	-3.38534100	-3.71309100
C	5.93074500	-2.82114800	-2.51430500
C	-3.97852900	-4.24161900	-2.50547200
C	5.85178500	-5.99108600	-0.30848100
C	5.66261500	-1.32469800	-2.50547200
C	8.11277900	-2.06962400	0.31098900
C	3.97733000	-4.24170100	2.50520100
C	-2.26404300	8.06068500	0.31098900
C	-1.68408500	5.56631700	-2.50547200
C	2.26254000	8.06333800	-0.30848100
C	1.68475600	5.56532000	2.50520100
C	-5.84873600	-5.99106100	0.31098900
C	-8.11432500	-2.07225200	-0.30848100
C	-5.66208600	-1.32361900	2.50520100
H	1.31014800	4.26049400	-1.69938200
H	-1.30994000	4.25889000	1.69812100
H	-3.03333700	-3.26388600	1.69812100
H	-4.34477000	-0.99562500	-1.69938200
H	-1.99174100	2.38032300	2.72698400
H	-2.52143300	1.16847700	1.55216800
H	-1.10132500	0.86734600	2.54652500
H	-0.20048100	-1.38744800	2.54652500
H	-1.06555000	-2.91505900	2.72698400
H	0.24878600	-2.76786400	1.55216800
H	-2.27184200	1.59817200	-1.55686100
H	-1.30390600	0.51712800	-2.55225200
H	-3.05964100	0.53382200	-2.72986600
H	1.09979800	0.87065200	-2.55225200
H	1.99212400	2.38281600	-2.72986600
H	2.51997800	1.16838700	-1.55686100
H	3.03462200	-3.26486900	-1.69938200
H	4.34327700	-0.99500400	1.69812100
H	2.27264800	1.59938700	1.55216800

H	1.30180600	0.52010200	2.54652500
H	3.05729000	0.53473700	2.72698400
H	0.20410700	-1.38777900	-2.55225200
H	1.06751800	-2.91663800	-2.72986600
H	-0.24813600	-2.76655900	-1.55686100
H	-6.67457500	-6.63167900	2.76655800
H	-6.54965800	-2.73975400	4.57560500
H	-9.08311900	-2.46810500	-2.76329300
H	-5.64988600	-4.30326600	-4.57444300
H	2.40411800	9.10026500	-2.76329300
H	-0.90179500	7.04457800	-4.57444300
H	-2.40591500	9.09619100	2.76655800
H	0.90213200	7.04204800	4.57560500
H	9.08049000	-2.46451200	2.76655800
H	5.64752600	-4.30229300	4.57560500
H	6.67900100	-6.63216000	-2.76329300
H	6.55168100	-2.74131200	-4.57444300
H	-3.83737000	-4.98706000	-3.29541000
H	-3.25951000	-3.43247700	-2.68104500
H	-3.70138700	-4.70769500	-1.55581200
H	4.80111500	-6.05988000	0.00145600
H	6.17287100	-7.00011000	-0.58571400
H	6.42715600	-5.68253100	0.56932900
H	6.23760600	-0.82973000	-3.29541000
H	4.60236700	-1.10658000	-2.68104500
H	5.92767700	-0.85164800	-1.55581200
H	7.64472800	-1.12725700	-0.00132900
H	9.14602300	-1.83969900	0.58961500
H	8.13667000	-2.72353000	-0.56576600
H	3.83343800	-4.98080100	3.30054800
H	3.25713500	-3.43160000	2.67102300
H	3.70442800	-4.71580800	1.55820400
H	-1.70968900	8.40832800	-0.56576600
H	-2.84613000	7.18415700	-0.00132900
H	-2.97978600	8.84053800	0.58961500
H	-2.40023600	5.81679000	-3.29541000
H	-1.34285700	4.53905700	-2.68104500
H	-2.22629000	5.55934300	-1.55581200
H	1.70763900	8.40734600	0.56932900
H	2.84745200	7.18782700	0.00145600
H	2.97583800	8.84591800	-0.58571400
H	2.39678100	5.81025500	3.30054800
H	1.34328500	4.53656100	2.67102300
H	2.23179500	5.56603200	1.55820400
H	-4.79859700	-6.05690000	-0.00132900
H	-6.16623700	-7.00083900	0.58961500
H	-6.42698100	-5.68479800	-0.56576600
H	-7.64856700	-1.12794800	0.00145600
H	-9.14870900	-1.84580800	-0.58571400
H	-8.13479500	-2.72481500	0.56932900
H	-6.23021900	-0.82945400	3.30054800
H	-4.60042000	-1.10496100	2.67102300
H	-5.93622300	-0.85022500	1.55820400
H	-0.97561200	8.65785500	4.74877200
H	-7.01011700	-5.17383200	4.74877200
H	7.98572900	-3.48402300	4.74877200
H	0.97489400	8.66175000	-4.74623300
H	-7.98874300	-3.48659200	-4.74623300
H	7.01384900	-5.17515800	-4.74623300

BFG1H

DFT B3LYP/6-31G, gas phase, S_0

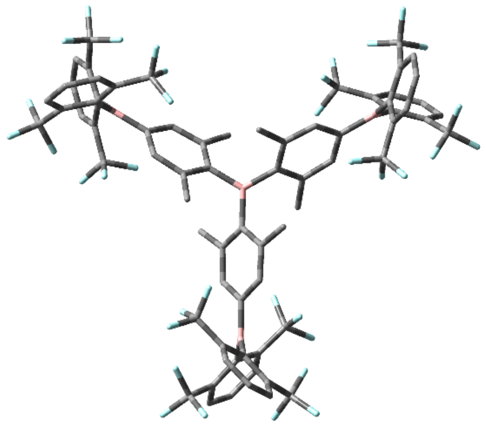

Point group: C_3

Total energy: –4,054,541.64 kcal mol^{-1}

Dipole moment: 0.01 D

Immaginary frequencies: 0

C	-0.00204800	1.58214400	-0.01101900
C	-1.36915200	-0.79284600	-0.01101900
C	0.76812600	2.31373300	-0.96530500
C	0.72133000	3.71129300	-0.96763400
C	-0.00146900	4.44422000	-0.00502400
C	-0.72514200	3.70750400	0.95393900
C	-0.77280600	2.30996500	0.94570100
C	-1.61408600	-1.82425200	0.94570100
C	-2.84822200	-2.48174300	0.95393900
C	-3.84807300	-2.22338200	-0.00502400
C	-3.57473900	-1.23095600	-0.96763400
C	-2.38781400	-0.49165000	-0.96530500
C	-1.62599400	1.62941600	2.00188300
C	-0.59811900	-2.22286000	2.00188300
C	-2.22725300	0.58442600	-2.02486000
C	1.61975400	1.63664500	-2.02486000
B	0.00000000	0.00000000	-0.01200600
C	1.37120100	-0.78929800	-0.01101900
C	1.61968800	-1.82208300	-0.96530500
C	2.85340900	-2.48033600	-0.96763400
C	3.84954100	-2.22083800	-0.00502400
C	3.57336400	-1.22576100	0.95393900
C	2.38689100	-0.48571300	0.94570100
C	2.22411300	0.59344400	2.00188300
C	0.60749900	-2.22107000	-2.02486000
B	0.00000000	5.99245800	-0.00066900
B	5.18962100	-2.99622900	-0.00066900
B	-5.18962100	-2.99622900	-0.00066900
C	-5.91505100	-3.28571700	-1.40690400
C	-5.79667600	-3.47407100	1.41033400
C	-0.11029500	6.75710400	1.41033400
C	0.11201100	6.76544300	-1.40690400
C	5.90697200	-3.28303300	1.41033400
C	5.80304000	-3.47972500	-1.40690400
C	-6.21179800	-4.81416000	1.66462100
C	-6.70514000	-5.23073400	2.90664200
C	-6.81895500	-4.32617400	3.95670300
C	-6.43819300	-3.00625900	3.75295200
C	-5.93592600	-2.57939500	2.51183200
C	-7.28414300	-2.97400400	-1.65447500
C	-7.89873000	-3.19555100	-2.89251300
C	-7.17879700	-3.75017700	-3.94507500
C	-5.84466600	-4.08164900	-3.74779000
C	-5.21673700	-3.85767100	-2.51071100
C	1.06650900	7.79525500	-1.65447500
C	1.18193700	8.43827600	-2.89251300
C	0.34165000	8.09210800	-3.94507500
C	-0.61247900	7.10245400	-3.74779000
C	-0.73247300	6.44666200	-2.51071100
C	-1.06328600	7.78665500	1.66462100
C	-1.17737900	8.42218900	2.90664200
C	-0.33709900	8.06847500	3.95670300
C	0.61560000	7.07876900	3.75295200
C	0.73414200	6.43036100	2.51183200
C	7.27508400	-2.97249400	1.66462100
C	7.88251900	-3.19145500	2.90664200
C	7.15605400	-3.74230100	3.95670300
C	5.82259400	-4.07250900	3.75295200
C	5.20178500	-3.85096600	2.51183200
C	6.21763500	-4.82125100	-1.65447500
C	6.71679300	-5.24272500	-2.89251300
C	6.83714600	-4.34193200	-3.94507500
C	6.45714500	-3.02080500	-3.74779000
C	5.94921000	-2.58899100	-2.51071100
C	-3.75585800	-4.24735200	-2.52714500
C	6.17064000	-5.87713000	-0.58952400
C	5.55624400	-1.12899300	-2.52714500
C	8.17263800	-2.40804900	0.60305000
C	3.74028000	-4.23849800	2.52149800
C	-2.00088800	8.28173600	0.60305000
C	-1.80038600	5.37634500	-2.52714500
C	2.00442400	8.28249600	-0.58952400
C	1.80050700	5.35842700	2.52149800
C	-6.17175000	-5.87368800	0.60305000
C	-8.17506400	-2.40536600	-0.58952400
C	-5.54078700	-1.11992800	2.52149800
H	1.29071600	4.24313500	-1.72374000
H	-1.29428400	4.23644200	1.71227300
H	-3.02172400	-3.23910400	1.71227300
H	-4.32002100	-1.00377500	-1.72374000
H	-1.98556900	2.36483800	2.72824600
H	-2.50189800	1.13274000	1.57212100
H	-1.07165800	0.86759200	2.56104600
H	-0.21552800	-1.36187900	2.56104600
H	-1.05522500	-2.90197300	2.72824600
H	0.26996700	-2.73307700	1.57212100
H	-2.22659400	1.59230400	-1.59740900
H	-1.29481000	0.47853000	-2.59036500
H	-3.04924300	0.53074600	-2.74540300
H	1.06182400	0.88207300	-2.59036500
H	1.98426100	2.37534900	-2.74540300
H	2.49227300	1.13213500	-1.59740900
H	3.02930500	-3.23936000	-1.72374000
H	4.31600800	-0.99733800	1.71227300
H	2.23193000	1.60033700	1.57212100
H	1.28718600	0.49428700	2.56104600
H	3.04079500	0.53713400	2.72824600
H	0.23298600	-1.36060300	-2.59036500
H	1.06498200	-2.90609500	-2.74540300
H	-0.26567900	-2.72443900	-1.59740900
H	-6.99374700	-6.26402200	3.04500600

H	-6.53944800	-2.28810400	4.55634700
H	-8.93827700	-2.92782900	-3.02588200
H	-5.27841800	-4.53138400	-4.55311100
H	1.93356400	9.20468900	-3.02588200
H	-1.28508500	6.83693600	-4.55311100
H	-1.92792900	9.18877300	3.04500600
H	1.28816800	6.80738000	4.55634700
H	8.92167600	-2.92475100	3.04500600
H	5.25128000	-4.51927600	4.55634700
H	7.00471200	-6.27686000	-3.02588200
H	6.56350300	-2.30555200	-4.55311100
H	-0.42113300	8.56044200	4.91856500
H	-7.20299400	-4.64493300	4.91856500
H	7.62412700	-3.91550900	4.91856500
H	0.42676100	8.58982800	-4.90387700
H	-7.65239000	-3.92532800	-4.90387700
H	7.22562900	-4.66450000	-4.90387700
F	3.17080500	-4.54825200	1.30174600
F	2.35349900	5.02012300	1.30174600
F	-5.52430400	-0.47187200	1.30174600
F	2.94831700	-3.23123300	3.08950700
F	1.32417100	4.16893400	3.08950700
F	-4.27248800	-0.93770100	3.08950700
F	3.53260100	-5.36386500	3.32868600
F	2.87894300	5.74125500	3.32868600
F	-6.41154400	-0.37738900	3.32868600
F	8.58717800	-3.36034100	-0.33051200
F	-1.38344800	9.11688500	-0.33051200
F	-7.20373000	-5.75654400	-0.33051200
F	7.56589600	-1.38559800	-0.12309000
F	-2.58298500	7.24505700	-0.12309000
F	-4.98291100	-5.85946000	-0.12309000
F	9.34173900	-1.86404500	1.12960900
F	-3.05655900	9.02220600	1.12960900
F	-6.28518000	-7.15816100	1.12960900
F	4.97777900	-5.85924500	0.12994500
F	2.58536500	7.24050500	0.12994500
F	-7.56314400	-1.38126100	0.12994500
F	7.19754900	-5.75782900	0.34935800
F	1.38765200	9.11217500	0.34935800
F	-8.58520100	-3.35434600	0.34935800
F	6.28566400	-7.16351200	-1.11101300
F	3.06095200	9.02530100	-1.11101300
F	-9.34661600	-1.86178800	-1.11101300
F	4.29052200	-0.94688500	-3.10079500
F	-2.96528800	-3.24225900	-3.10079500
F	-1.32523500	4.18914400	-3.10079500
F	5.53599700	-0.47671100	-1.30971100
F	-3.18084200	-4.55595900	-1.30971100
F	-2.35515500	5.03267000	-1.30971100
F	6.43130900	-0.39057200	-3.33345600
F	-3.55390000	-5.37439100	-3.33345600
F	-2.87740900	5.76496300	-3.33345600

BG1NMe$_2$

DFT B3LYP/6-31G, gas phase, S_0

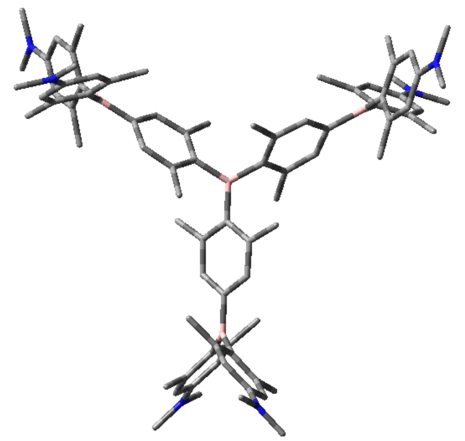

Point group: C_3

Total energy: –2,317,460.76 kcal mol^{-1}

Dipole moment: 0.01 D

Immaginary frequencies: 0

C	-0.00040700	1.58632700	-0.00249300
C	-1.37359600	-0.79351600	-0.00249300
C	0.78137300	2.32495600	-0.94314800
C	0.74281000	3.72621400	-0.94165400
C	0.00009500	4.46734000	-0.00066200
C	-0.74290600	3.72530500	0.93937000
C	-0.78206500	2.32401400	0.93903000
C	-1.62162300	-1.83929600	0.93903000
C	-2.85475600	-2.50602900	0.93937000
C	-3.86887700	-2.23358800	-0.00066200
C	-3.59840100	-1.21981500	-0.94165400
C	-2.40415700	-0.48578900	-0.94314800
C	-1.64982900	1.64794700	1.98737300
C	-0.60224900	-2.25276800	1.98737300
C	-2.25313600	0.60290800	-1.99241900
C	1.64870200	1.64981900	-1.99241900
B	0.00000000	0.00000000	-0.00281500
C	1.37400300	-0.79281100	-0.00249300
C	1.62278500	-1.83916700	-0.94314800
C	2.85559100	-2.50639900	-0.94165400
C	3.86878300	-2.23375200	-0.00066200
C	3.59766200	-1.21927700	0.93937000
C	2.40368800	-0.48471900	0.93903000
C	2.25207900	0.60482000	1.98737300
C	0.60443400	-2.25272700	-1.99241900
B	0.00000000	6.04302000	0.00000800
B	5.23340900	-3.02151000	0.00000800
B	-5.23340900	-3.02151000	0.00000800
C	-5.97978900	-3.24721100	-1.37072800
C	-5.80081000	-3.55486000	1.37137700
C	-0.17819400	6.80107900	1.37137700
C	0.17772700	6.80225500	-1.37072800
C	5.97900400	-3.24621900	1.37137700
C	5.80206200	-3.55504400	-1.37072800
C	-6.18571300	-4.92066200	1.53602800
C	-6.65725900	-5.39754900	2.76296400
C	-6.80963900	-4.54964900	3.88224800
C	-6.45705600	-3.19246600	3.71734600
C	-5.95021700	-2.70424700	2.50859700
C	-7.35586700	-2.90013400	-1.53440900
C	-8.00520100	-3.07115400	-2.76084500
C	-7.34694000	-3.62594500	-3.88064600
C	-5.99463300	-3.99704200	-3.71665500
C	-5.31783300	-3.80102900	-2.50839200
C	1.16634400	7.82043500	-1.53440900
C	1.34290300	8.46828400	-2.76084500
C	0.53331000	8.17560900	-3.88064600
C	-0.46422300	7.19002600	-3.71665500
C	-0.63287100	6.50589300	-2.50839200
C	-1.16856200	7.81731600	1.53602800
C	-1.34578500	8.46413000	2.76296400
C	-0.53529200	8.17214500	3.88224800
C	0.46377100	7.18820700	3.71734600
C	0.63316200	6.50516200	2.50859700
C	7.35427500	-2.89665400	1.53602800
C	8.00304400	-3.06658100	2.76296400
C	7.34493100	-3.62249600	3.88224800
C	5.99328500	-3.99574100	3.71734600
C	5.31705400	-3.80091500	2.50859700
C	6.18952300	-4.92030100	-1.53440900
C	6.66229800	-5.39713000	-2.76084500
C	6.81363000	-4.54966500	-3.88064600
C	6.45885700	-3.19298400	-3.71665500
C	5.95070500	-2.70486400	-2.50839200
C	-3.86490300	-4.24954700	-2.46221300
C	6.07547500	-5.92700800	-0.40187900
C	5.61266700	-1.22233000	-2.46221300
C	8.16869300	-2.29288900	0.40405600
C	3.86485800	-4.25157500	2.46096000
C	-2.09864600	8.22074000	0.40405600
C	-1.74776500	5.47187800	-2.46221300
C	2.09520200	8.22502000	-0.40187900
C	1.74954300	5.47285200	2.46096000
C	-6.07004600	-5.92785100	0.40405600
C	-8.17067700	-2.29801200	-0.40187900
C	-5.61440100	-1.22127700	2.46096000
H	1.31717000	4.26324400	-1.69231100
H	-1.31700800	4.26158700	1.69075600
H	-3.03213900	-3.27135600	1.69075600
H	-4.35066300	-0.99091900	-1.69231100
H	-2.00339200	2.38403400	2.71670900
H	-2.52835000	1.16974800	1.54111200
H	-1.11264700	0.86955100	2.54089200
H	-0.19673000	-1.39835600	2.54089200
H	-1.06293800	-2.92700600	2.71670900
H	0.25114300	-2.77448900	1.54111200
H	-2.27841200	1.60323500	-1.54707600
H	-1.31055800	0.52656600	-2.54609700
H	-3.06756400	0.54022200	-2.72149100
H	1.11129800	0.87169300	-2.54609700
H	2.00162800	2.38647700	-2.72149100
H	2.52764800	1.17154600	-1.54707600
H	3.03349300	-3.27232500	-1.69231100
H	4.34914700	-0.99023100	1.69075600
H	2.27720600	1.60474100	1.54111200
H	1.30937700	0.52880500	2.54089200
H	3.06633000	0.54297100	2.71670900
H	0.19925900	-1.39825900	-2.54609700
H	1.06593600	-2.92669900	-2.72149100
H	-0.24923600	-2.77478000	-1.54707600

H	-6.90944100	-6.44808500	2.84049500
H	-6.58124200	-2.49653700	4.53830900
H	-9.04165300	-2.76603400	-2.83766300
H	-5.45390600	-4.45182500	-4.53794600
H	2.12537000	9.21331800	-2.83766300
H	-1.12844100	6.94913400	-4.53794600
H	-2.12948400	9.20779400	2.84049500
H	1.12855600	6.94779100	4.53830900
H	9.03892600	-2.75970900	2.84049500
H	5.45268500	-4.45125400	4.53830900
H	6.91628200	-6.44728400	-2.83766300
H	6.58234700	-2.49730900	-4.53794600
H	-3.66110100	-4.97230100	-3.25970000
H	-3.17916000	-3.40484200	-2.59376400
H	-3.60259100	-4.72014000	-1.51001100
H	5.05850700	-5.97386600	0.00486600
H	6.33629800	-6.93158400	-0.75114600
H	6.73799100	-5.67454900	0.43178100
H	6.13669000	-0.68445600	-3.25970000
H	4.53825900	-1.05081300	-2.59376400
H	5.88905700	-0.75986500	-1.51001100
H	7.69859800	-1.39038600	-0.00382700
H	9.16785600	-2.01333600	0.75433400
H	8.28439100	-2.99306600	-0.42903200
H	3.65969900	-4.97014200	3.26186500
H	3.17760200	-3.40712100	2.58568200
H	3.60603200	-4.72808900	1.51064600
H	-1.55012400	8.67102600	-0.42903200
H	-2.64518900	7.36237500	-0.00382700
H	-2.84032800	8.94626500	0.75433400
H	-2.47558900	5.65675700	-3.25970000
H	-1.35909900	4.45565400	-2.59376400
H	-2.28646600	5.48000500	-1.51001100
H	1.54530800	8.67254600	0.43178100
H	2.64426600	7.36772900	0.00486600
H	2.83477900	8.95318700	-0.75114600
H	2.47442000	5.65446400	3.26186500
H	1.36185200	4.45544500	2.58568200
H	2.29162900	5.48696000	1.51064600
H	-5.05340900	-5.97198800	-0.00382700
H	-6.32752900	-6.93292800	0.75433400
H	-6.73426700	-5.67796000	-0.42903200
H	-7.70277300	-1.39386300	0.00486600
H	-9.17107600	-2.02160300	-0.75114600
H	-8.28329900	-2.99799700	0.43178100
H	-6.13411900	-0.68432200	3.26186500
H	-4.53945400	-1.04832400	2.58568200
H	-5.89766100	-0.75887100	1.51064600
N	7.29864500	-5.03101600	-5.09358600
N	8.00372700	-3.80087300	5.09566500
N	0.70766500	8.83632000	-5.09358600
N	-8.00631000	-3.80530400	-5.09358600
C	-9.41455100	-3.44062700	-5.22456500
H	10.04934200	-4.00224100	-4.52452700
H	-9.57890300	-2.36909700	-5.04140600
H	-9.75029200	-3.66460300	-6.23852400
C	7.68694600	-6.43292700	-5.22456500
H	8.49071300	-6.70186500	-4.52452700
H	6.84115000	-7.11102500	-5.04140600
H	8.04878500	-6.61169900	-6.23852400
C	1.72760500	9.87355400	-5.22456500
H	1.55862800	10.70410600	-4.52452700
H	2.73775300	9.48012200	-5.04140600
H	1.70150700	10.27630200	-6.23852400
C	-7.29567100	-4.36803800	-6.23877700
H	-6.42465600	-3.75823100	-6.51690800
H	-6.94094500	-5.39006900	-6.04137700

H	-7.96944500	-4.40468100	-7.09649800
C	7.43066700	-4.13421700	-6.23877700
H	6.46705200	-3.68480000	-6.51690800
H	8.13840900	-3.31600000	-6.04137700
H	7.79928900	-4.69940100	-7.09649800
C	-0.13499600	8.50225500	-6.23877700
H	-0.04239600	7.44303100	-6.51690800
H	-1.19746400	8.70606900	-6.04137700
H	0.17015700	9.10408300	-7.09649800
C	-7.67939700	-6.43346700	5.22768300
H	-8.48306700	-6.70418500	4.52821400
H	-6.83255800	-7.11026300	5.04450600
H	-8.04043900	-6.61221900	6.24192800
C	9.41124500	-3.43382000	5.22768300
H	10.04752800	-3.99446000	4.52821400
H	9.57394800	-2.36203700	5.04450600
H	9.74656900	-3.65711500	6.24192800
C	-1.73184700	9.86728700	5.22768300
H	-1.56446100	10.69864400	4.52821400
H	-2.74139000	9.47230000	5.04450600
H	-1.70613000	10.26933400	6.24192800
C	-7.42631600	-4.13376200	6.24043300
H	-6.46324600	-3.68273400	6.51785600
H	-8.13539200	-3.31673000	6.04293100
H	-7.79367900	-4.69902800	7.09864200
C	7.29310100	-4.36449700	6.24043300
H	6.42096400	-3.75596900	6.51785600
H	6.94006800	-5.38709200	6.04293100
H	7.96631700	-4.40001000	7.09864200
C	0.13321500	8.49825900	6.24043300
H	0.04228200	7.43870200	6.51785600
H	1.19532400	8.70382100	6.04293100
H	-0.17263800	9.09903800	7.09864200
N	-0.71021100	8.83186800	5.09566500
N	-7.29351600	-5.03099400	5.09566500

BG1Br

DFT B3LYP/6-31G, gas phase, S_0

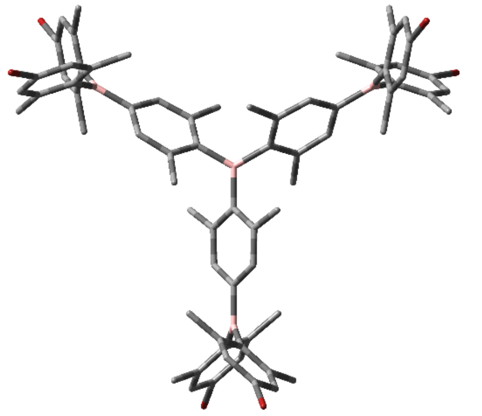

Point group: C_3

Total energy: – 11,493,076.47 kcal mol^{-1}

Dipole moment: 0.01 D

Immaginary frequencies: 0

C	-0.00024900	-1.58702600	0.00245500
C	-1.37428100	0.79372900	0.00245500
C	0.77700600	-2.32330600	0.94866900
C	0.73838800	-3.72405600	0.94734700
C	0.00010100	-4.46382100	0.00075900
C	-0.73842300	-3.72311800	-0.94492600
C	-0.77749800	-2.32236100	-0.94458300
C	-1.62247400	1.83451400	-0.94458300
C	-2.85510300	2.50105200	-0.94492600
C	-3.86583300	2.23182200	0.00075900
C	-3.59432100	1.22256500	0.94734700
C	-2.40054500	0.48874600	0.94866900
C	-1.63996300	-1.64547100	-1.99605500
C	-0.60503800	2.24298500	-1.99605500
C	-2.24612300	-0.59594700	2.00083400
C	1.63916700	-1.64722600	2.00083400
B	0.00000000	0.00000000	0.00276300
C	1.37453000	0.79329700	0.00245500
C	1.62353900	1.83456000	0.94866900
C	2.85593300	2.50149100	0.94734700
C	3.86573100	2.23199800	0.00075900
C	3.59352600	1.22206600	-0.94492600
C	2.39997300	0.48784700	-0.94458300
C	2.24500100	-0.59751400	-1.99605500
C	0.60695600	2.24317300	2.00083400
B	0.00000000	-6.03143200	0.00000500
B	5.22337300	3.01571600	0.00000500
B	-5.22337300	3.01571600	0.00000500
C	-6.02702900	3.15730000	1.35911600
C	-5.74632000	3.64054300	-1.35981800
C	-0.27964300	-6.79673100	-1.35981800
C	0.27921200	-6.79821000	1.35911600
C	6.02596300	3.15618800	-1.35981800
C	5.74781700	3.64091000	1.35911600
C	-6.04245200	5.03211100	-1.46703500
C	-6.48148300	5.57196600	-2.68660600
C	-6.65143900	4.74400600	-3.78673800
C	-6.38959600	3.38387100	-3.71637100
C	-5.92390600	2.82314900	-2.51572000
C	-7.38058800	2.71865000	1.46582800
C	-8.06813500	2.82969300	2.68501500
C	-7.43629500	3.39078800	3.78533100
C	-6.12726900	3.84348000	3.71543300
C	-5.40829800	3.71988800	2.51513900
C	1.33587400	-7.75110100	1.46582800
C	1.58348100	-8.40205700	2.68501500
C	0.78163900	-8.13541500	3.78533100
C	-0.26491700	-7.22811000	3.71543300
C	-0.51736900	-6.54366800	2.51513900
C	-1.33671000	-7.74897200	-1.46703500
C	-1.58472300	-8.39911200	-2.68660600
C	-0.78271000	-8.13231900	-3.78673800
C	0.26428000	-7.22548800	-3.71637100
C	0.51703400	-6.54182800	-2.51572000
C	7.37916200	2.71686100	-1.46703500
C	8.06620600	2.82714600	-2.68660600
C	7.43415000	3.38831200	-3.78673800
C	6.12531600	3.84161700	-3.71637100
C	5.40687200	3.71867900	-2.51572000
C	6.04471300	5.03245200	1.46582800
C	6.48465400	5.57236300	2.68501500
C	6.65465600	4.74462700	3.78533100
C	6.39218500	3.38463000	3.71543300
C	5.92566700	2.82378000	2.51513900
C	-3.97678000	4.23038700	2.51638400
C	5.87198100	5.98848200	0.30075600
C	5.65201200	1.32879900	2.51638400
C	8.12105600	2.08982300	-0.30180900
C	3.97558200	4.22972800	-2.51572900
C	-2.25068900	-8.07795200	-0.30180900
C	-1.67523300	-5.55918600	2.51638400
C	2.25018700	-8.07952600	0.30075600
C	1.67526100	-5.55781900	-2.51572900
C	-5.87036700	5.98813000	-0.30180900
C	-8.12216800	2.09104400	0.30075600
C	-5.65084300	1.32809100	-2.51572900
H	1.31120500	-4.25949500	1.69978900
H	-1.31104400	-4.25782600	-1.69803300
H	-3.03186400	3.26431100	-1.69803300
H	-4.34443400	0.99421000	1.69978900
H	-1.98931800	-2.37924600	-2.72915300
H	-2.52178500	-1.17046800	-1.55296600
H	-1.10093600	-0.86601100	-2.54577200
H	-0.19951900	1.38644400	-2.54577200
H	-1.06582900	2.91242300	-2.72915300
H	0.24723800	2.76916400	-1.55296600
H	-2.27390700	-1.59737000	1.55819800
H	-1.30249800	-0.51743000	2.55195100
H	-3.05744400	-0.53211000	2.73267500
H	1.09935600	-0.86928100	2.55195100
H	1.98954300	-2.38176900	2.73267500
H	2.52031600	-1.17057600	1.55819800
H	3.03322800	3.26528500	1.69978900
H	4.34290800	0.99351600	-1.69803300
H	2.27454800	-1.59869600	-1.55296600
H	1.30045600	-0.52043300	-2.54577200
H	3.05514700	-0.53317600	-2.72915300
H	0.20314100	1.38671100	2.55195100
H	1.06790100	2.91387900	2.73267500
H	-0.24640900	2.76794600	1.55819800
H	-6.69291400	6.63184000	-2.76571000
H	-6.54384500	2.75471200	-4.58545200

H	-9.09195400	2.48343800	2.76373600
H	-5.65973500	4.29169600	4.58460900
H	2.39525700	-9.11558200	2.76373600
H	-0.88685000	-7.04732300	4.58460900
H	-2.39688500	-9.11215400	-2.76571000
H	0.88627200	-7.04449200	-4.58545200
H	9.08979900	2.48031300	-2.76571000
H	5.65757300	4.28978000	-4.58545200
H	6.69669800	6.63214400	2.76373600
H	6.54658500	2.75562700	4.58460900
H	-3.83881100	4.97969800	3.30252100
H	-3.26479000	3.41792300	2.70326800
H	-3.69078100	4.68799300	1.56569200
H	4.82850500	6.03834500	-0.03406300
H	6.16898300	7.00190900	0.58625000
H	6.47534100	5.69052700	-0.56175400
H	6.23195100	0.83465800	3.30252100
H	4.59240300	1.11842900	2.70326800
H	5.90531100	0.85231400	1.56569200
H	7.63912900	1.16477900	0.03785300
H	9.14525600	1.83457800	-0.58923400
H	8.17027400	2.76427100	0.55816700
H	3.83467700	4.97230400	-3.30766900
H	3.26248900	3.41599000	-2.69233200
H	3.69391600	4.69582400	-1.56776900
H	-1.69120900	-8.45780000	0.55816700
H	-2.81083600	-7.19806900	0.03785300
H	-2.98383600	-8.83731300	-0.58923400
H	-2.39314000	-5.81435700	3.30252100
H	-1.32761300	-4.53635300	2.70326800
H	-2.21453100	-5.54030600	1.56569200
H	1.69047000	-8.45307300	-0.56175400
H	2.81510800	-7.20078000	-0.03406300
H	2.97934000	-8.84345000	0.58625000
H	2.38880400	-5.80707900	-3.30766900
H	1.32709000	-4.53339300	-2.69233200
H	2.21974500	-5.54693700	-1.56776900
H	-4.82829300	6.03329000	0.03785300
H	-6.16141900	7.00273500	-0.58923400
H	-6.47906600	5.69353000	0.55816700
H	-7.64361300	1.16243500	-0.03406300
H	-9.14832200	1.84154100	0.58625000
H	-8.16581200	2.76254600	-0.56175400
H	-6.22348000	0.83477500	-3.30766900
H	-4.58957800	1.11740300	-2.69233200
H	-5.91366100	0.85111300	-1.56776900
Br	7.28443600	5.50869900	5.46372100
Br	8.41012700	3.55086600	-5.46559200
Br	1.12845500	-9.06285700	5.46372100
Br	-1.12992300	-9.05881700	-5.46559200
Br	-8.41289200	3.55415700	5.46372100
Br	-7.28020400	5.50795000	-5.46559200

BG1Me

DFT B3LYP/6-31G, gas phase, S_0

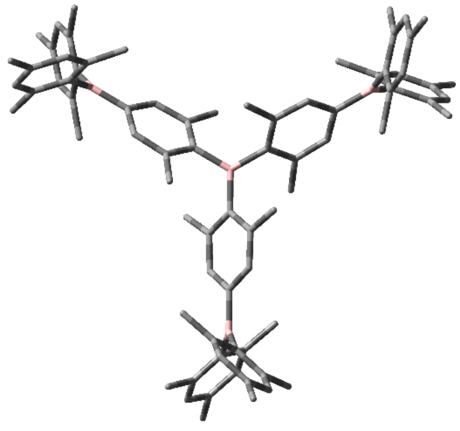

Point group: C_3

Total energy: –1,961,212.46 kcal mol^{-1}

Dipole moment: 0.004 D

Immaginary frequencies: 0

C	0.00026800	1.58665800	0.00244500
C	1.37395200	-0.79356100	0.00244500
C	-0.77777700	2.32365300	0.94739500
C	-0.73880400	3.72455400	0.94623400
C	-0.00004800	4.46451600	0.00073100
C	0.73894200	3.72358600	-0.94382800
C	0.77832500	2.32269800	-0.94328600
C	1.62235300	-1.83539800	-0.94328600
C	2.85524900	-2.50173600	-0.94382800
C	3.86640800	-2.23221600	0.00073100
C	3.59496100	-1.22245400	0.94623400
C	2.40123100	-0.48825200	0.94739500
C	1.64172600	1.64584700	-1.99429800
C	0.60448200	-2.24469900	-1.99429800
C	2.24742600	0.59722300	1.99915500
C	-1.64092400	1.64771600	1.99915500
B	0.00000000	0.00000000	0.00273100
C	-1.37422000	-0.79309700	0.00244500
C	-1.62345400	-1.83540100	0.94739500
C	-2.85615700	-2.50210000	0.94623400
C	-3.86636000	-2.23230000	0.00073100
C	-3.59419100	-1.22185000	-0.94382800
C	-2.40067800	-0.48730000	-0.94328600
C	-2.24620800	0.59885300	-1.99429800
C	-0.60650200	-2.24494000	1.99915500
B	0.00000000	6.03514200	-0.00015100
B	-5.22658600	-3.01757100	-0.00015100
B	5.22658600	-3.01757100	-0.00015100
C	6.01890500	-3.17334200	1.36248700
C	5.75608900	-3.62532200	-1.36361300
C	0.26157600	6.79758000	-1.36361300
C	-0.26125700	6.79919600	1.36248700
C	-6.01766500	-3.17225800	-1.36361300
C	-5.75764700	-3.62585300	1.36248700
C	6.05775400	-5.01412400	-1.48907900
C	6.50074100	-5.53313300	-2.71439400
C	6.69233300	-4.72101000	-3.83893200
C	6.40856500	-3.35594100	-3.71353600
C	5.93637300	-2.80151000	-2.51447700
C	7.37288100	-2.74080800	1.48709200
C	8.04471900	-2.86584900	2.71174500
C	7.43774900	-3.43799000	3.83660200
C	6.11346000	-3.87405000	3.71206300
C	5.39629700	-3.74151800	2.51354400
C	-1.31283100	7.75550600	1.48709200
C	-1.54046200	8.39985600	2.71174500
C	-0.74148800	8.16027500	3.83660200
C	0.29829600	7.23143700	3.71206300
C	0.54210100	6.54409000	2.51354400
C	1.31348200	7.75323100	-1.48907900
C	1.54146300	8.39637300	-2.71439400
C	0.74234800	8.15623600	-3.83893200
C	-0.29795300	7.22795100	-3.71353600
C	-0.54200800	6.54180400	-2.51447700
C	-7.37123600	-2.73910700	-1.48907900
C	-8.04220400	-2.86324100	-2.71439400
C	-7.43468100	-3.43522600	-3.83893200
C	-6.11061300	-3.87201000	-3.71353600
C	-5.39436500	-3.74029500	-2.51447700
C	-6.06005000	-5.01469800	1.48709200
C	-6.50425800	-5.53400700	2.71174500
C	-6.69626100	-4.72228500	3.83660200
C	-6.41175600	-3.35738700	3.71206300
C	-5.93839800	-2.80257100	2.51354400
C	3.96225100	-4.24635600	2.50141800
C	-5.87924300	-5.98403800	0.33335300
C	-5.65857800	-1.30823100	2.50141800
C	-8.12107800	-2.09861600	-0.33540400
C	-3.96064300	-4.24594500	-2.50087000
C	2.24308500	8.08236800	-0.33540400
C	1.69632700	5.55458800	2.50141800
C	-2.24270700	8.08359300	0.33335300
C	-1.69677500	5.55299000	-2.50087000
C	5.87799300	-5.98375200	-0.33540400
C	8.12195100	-2.09955500	0.33335300
C	5.65741800	-1.30704500	-2.50087000
H	-1.31030300	4.26109300	1.69904200
H	1.31024900	4.25933800	-1.69733700
H	3.03357100	-3.26437800	-1.69733700
H	4.34536600	-0.99579100	1.69904200
H	1.99249400	2.38068100	-2.72591100
H	2.52236400	1.16891800	-1.55090400
H	1.10259100	0.86722700	-2.54536600
H	0.19974500	-1.38848500	-2.54536600
H	1.06548300	-2.91589100	-2.72591100
H	-0.24886900	-2.76889000	-1.55090400
H	2.27272600	1.59860100	1.55623800
H	1.30463200	0.51808400	2.55185200
H	3.06054500	0.53414200	2.72931400
H	-1.10099000	0.87080200	2.55185200
H	-1.99285300	2.38343900	2.72931400
H	-2.52079200	1.16893800	1.55623800
H	-3.03506300	-3.26530200	1.69904200
H	-4.34381900	-0.99496000	-1.69733700
H	-2.27349500	1.59997200	-1.55090400
H	-1.30233600	0.52125800	-2.54536600
H	-3.05797700	0.53521000	-2.72591100
H	-0.20364200	-1.38888600	2.55185200
H	-1.06769200	-2.91758100	2.72931400
H	0.24806600	-2.76753900	1.55623800
H	6.70695400	-6.59831100	-2.78843800
H	6.55694700	-2.70303400	-4.57078800

H	9.07056100	-2.51243100	2.78510600
H	5.62269900	-4.32914900	4.56951200
H	-2.35945200	9.11155100	2.78510600
H	0.93780400	7.03397500	4.56951200
H	2.36082800	9.10754800	-2.78843800
H	-0.93757800	7.03000000	-4.57078800
H	-9.06778200	-2.50923700	-2.78843800
H	-5.61937000	-4.32696600	-4.57078800
H	-6.71110900	-6.59912100	2.78510600
H	-6.56050300	-2.70482600	4.56951200
H	3.80870600	-4.97924000	3.30069700
H	3.24791200	-3.42946400	2.65828300
H	3.69044200	-4.72411000	1.55574900
H	-4.83065000	-6.05144200	0.01639700
H	-6.19462900	-6.99121200	0.62377400
H	-6.46144500	-5.68646200	-0.54379900
H	-6.21650200	-0.80881600	3.30069700
H	-4.59395900	-1.09804300	2.65828300
H	-5.93642000	-0.83396100	1.55574900
H	-7.65216600	-1.16000200	-0.01357500
H	-9.14917500	-1.86288500	-0.62808200
H	-8.15985600	-2.75461000	0.53932700
H	-3.80461600	-4.97339900	-3.30458600
H	-3.24524500	-3.42843500	-2.64923900
H	-3.69274200	-4.73051800	-1.55748400
H	1.69436500	8.44394800	0.53932700
H	2.82149200	7.20697100	-0.01357500
H	2.96128200	8.85486100	-0.62808200
H	2.40779600	5.78805600	3.30069700
H	1.34604700	4.52750700	2.65828300
H	2.24597800	5.55807100	1.55574900
H	-1.69389800	8.43900600	-0.54379900
H	-2.82537700	7.20918700	0.01639700
H	-2.95725300	8.86031200	0.62377400
H	-2.40478200	5.78159300	-3.30458600
H	-1.34649000	4.52468200	-2.64923900
H	-2.25037800	5.56326700	-1.55748400
H	4.83067400	-6.04696900	-0.01357500
H	6.18789300	-6.99197600	-0.62808200
H	6.46549100	-5.68933700	0.53932700
H	7.65602700	-1.15774500	0.01639700
H	9.15188100	-1.86910000	0.62377400
H	8.15534300	-2.75254400	-0.54379900
H	6.20939800	-0.80819400	-3.30458600
H	4.59173400	-1.09624700	-2.64923900
H	5.94312000	-0.83274900	-1.55748400
C	7.21865800	-5.29422600	-5.13593700
H	8.31734900	-5.28703600	-5.15802800
H	6.89900500	-6.33295400	-5.27363900
H	6.87296200	-4.71521100	-5.99914300
C	-8.19426300	-3.60442800	-5.13593700
H	-8.73738200	-4.55951700	-5.15802800
H	-8.93400200	-2.80823600	-5.27363900
H	-7.51997400	-3.59455400	-5.99914300
C	0.97560600	8.89865400	-5.13593700
H	0.42003300	9.84655400	-5.15802800
H	2.03499700	9.14119000	-5.27363900
H	0.64701200	8.30976500	-5.99914300
C	-0.97473900	8.90406300	5.13282400
H	-0.43169400	9.85932900	5.14746500
H	-2.03620300	9.13323100	5.27771600
H	-0.63205800	8.32247600	5.99544200
C	-7.22377500	-5.29618000	5.13282400
H	-8.32258300	-5.30352300	5.14746500
H	-6.89150900	-6.33001900	5.27771600
H	-6.89144600	-4.70861600	5.99544200
C	8.19851400	-3.60788300	5.13282400
H	8.75427700	-4.55580700	5.14746500
H	8.92771200	-2.80321200	5.27771600
H	7.52350400	-3.61385900	5.99544200

BG2H

DFT B3LYP/6-31G, gas phase, S_0

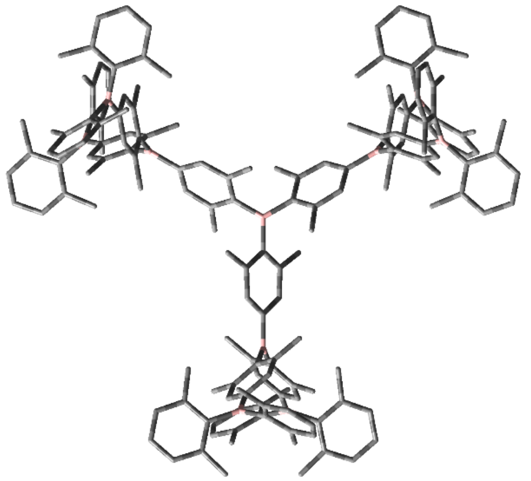

Point group: D_3

Total energy: –4,240,486.04 kcal mol^{-1}

Dipole moment: 0 D

Immaginary frequencies: 0

C	0.00000000	1.58692600	0.00000000
C	1.37431800	-0.79346300	0.00000000
C	-0.77637300	2.32275000	0.94731000
C	-0.73694700	3.72342800	0.94718400
C	0.00000000	4.46336400	0.00000000
C	0.73694700	3.72342800	-0.94718400
C	0.77637300	2.32275000	-0.94731000
C	1.62337400	-1.83373300	-0.94731000
C	2.85611000	-2.49992800	-0.94718400
C	3.86538700	-2.23168200	0.00000000
C	3.59305700	-1.22350000	0.94718400
C	2.39974700	-0.48901700	0.94731000
C	1.63804000	1.64619600	-1.99962800
C	0.60662800	-2.24168200	-1.99962800
C	2.24466700	0.59548600	1.99962800
C	-1.63804000	1.64619600	1.99962800
B	0.00000000	0.00000000	0.00000000
C	-1.37431800	-0.79346300	0.00000000
C	-1.62337400	-1.83373300	0.94731000
C	-2.85611000	-2.49992800	0.94718400
C	-3.86538700	-2.23168200	0.00000000
C	-3.59305700	-1.22350000	-0.94718400
C	-2.39974700	-0.48901700	-0.94731000
C	-2.24466700	0.59548600	-1.99962800
C	-0.60662800	-2.24168200	1.99962800
B	0.00000000	6.03117400	0.00000000
B	-5.22315000	-3.01558700	0.00000000
B	5.22315000	-3.01558700	0.00000000
C	6.03365200	-3.14504200	1.35784000
C	5.74051300	-3.65277500	-1.35784000
C	0.29314000	6.79781700	-1.35784000
C	-0.29314000	6.79781700	1.35784000
C	-6.03365200	-3.14504200	-1.35784000
C	-5.74051300	-3.65277500	1.35784000
C	6.01255300	-5.04966700	-1.46833300
C	6.44232200	-5.58558600	-2.68972600
C	6.67452300	-4.78890200	-3.82974200
C	6.42544800	-3.40736100	-3.69762100
C	5.94268600	-2.83642300	-2.51239800
C	7.37941600	-2.68219000	1.46833300
C	8.05842000	-2.78642200	2.68972600
C	7.48457200	-3.38585600	3.82974200
C	6.16358500	-3.86092100	3.69762100
C	5.42775800	-3.72830600	2.51239800
C	-1.36686400	7.73185700	1.46833300
C	-1.61609800	8.37200700	2.68972600
C	-0.81004900	8.17475700	3.82974200
C	0.26186300	7.26828200	3.69762100
C	0.51492800	6.56472900	2.51239800
C	1.36686400	7.73185700	-1.46833300
C	1.61609800	8.37200700	-2.68972600
C	0.81004900	8.17475700	-3.82974200
C	-0.26186300	7.26828200	-3.69762100
C	-0.51492800	6.56472900	-2.51239800
C	-7.37941600	-2.68219000	-1.46833300
C	-8.05842000	-2.78642200	-2.68972600
C	-7.48457200	-3.38585600	-3.82974200
C	-6.16358500	-3.86092100	-3.69762100
C	-5.42775800	-3.72830600	-2.51239800
C	-6.01255300	-5.04966700	1.46833300
C	-6.44232200	-5.58558600	2.68972600
C	-6.67452300	-4.78890200	3.82974200
C	-6.42544800	-3.40736100	3.69762100
C	-5.94268600	-2.83642300	2.51239800
C	3.99947700	-4.24874300	2.51073800
C	-5.81177200	-6.00174300	0.30428300
C	-5.67925800	-1.33927700	2.51073800
C	-8.10354800	-2.03227100	-0.30428300
C	-3.99947700	-4.24874300	-2.51073800
C	2.29177600	8.03401400	-0.30428300
C	1.67978100	5.58802000	2.51073800
C	-2.29177600	8.03401400	0.30428300
C	-1.67978100	5.58802000	-2.51073800
C	5.81177200	-6.00174300	-0.30428300
C	8.10354800	-2.03227100	0.30428300
C	5.67925800	-1.33927700	-2.51073800
H	-1.30727600	4.25898500	1.70142400
H	1.30727600	4.25898500	-1.70142400
H	3.03475100	-3.26162700	-1.70142400
H	4.34202700	-0.99735800	1.70142400
H	1.98848300	2.38082100	-2.73142300
H	2.51911500	1.16908300	-1.55739300
H	1.09790900	0.86845100	-2.55076600
H	0.20314600	-1.38504300	-2.55076600
H	1.06761000	-2.91248700	-2.73142300
H	-0.24710200	-2.76615900	-1.55739300
H	2.27201300	1.59707600	1.55739300
H	1.30105600	0.51659100	2.55076600
H	3.05609300	0.53166600	2.73142300
H	-1.09790900	0.86845100	2.55076600
H	-1.98848300	2.38082100	2.73142300
H	-2.51911500	1.16908300	1.55739300
H	-3.03475100	-3.26162700	1.70142400
H	-4.34202700	-0.99735800	-1.70142400
H	-2.27201300	1.59707600	-1.55739300
H	-1.30105600	0.51659100	-2.55076600
H	-3.05609300	0.53166600	-2.73142300

H	-0.20314600	-1.38504300	2.55076600
H	-1.06761000	-2.91248700	2.73142300
H	0.24710200	-2.76615900	1.55739300
H	6.61643500	-6.65633300	-2.75461200
H	6.60913400	-2.75849800	-4.55011300
H	9.07277000	-2.40183400	2.75461200
H	5.69349700	-4.34442900	4.55011300
H	-2.45633600	9.05816700	2.75461200
H	0.91563700	7.10292700	4.55011300
H	2.45633600	9.05816700	-2.75461200
H	-0.91563700	7.10292700	-4.55011300
H	-9.07277000	-2.40183400	-2.75461200
H	-5.69349700	-4.34442900	-4.55011300
H	-6.61643500	-6.65633300	2.75461200
H	-6.60913400	-2.75849800	4.55011300
H	3.85996700	-4.97881600	3.31480600
H	3.27787200	-3.43856600	2.66975400
H	3.72509500	-4.73415100	1.56967100
H	-4.75998100	-6.05435500	-0.00473800
H	-6.11766600	-7.01538000	0.58098700
H	-6.39201000	-5.70175300	-0.57377600
H	-6.24176500	-0.85342100	3.31480600
H	-4.61682100	-1.11943700	2.66975400
H	-5.96244300	-0.85895100	1.56967100
H	-7.62321600	-1.09508700	0.00473800
H	-9.13433000	-1.79036400	-0.58098700
H	-8.13386800	-2.68476700	0.57377600
H	-3.85996700	-4.97881600	-3.31480600
H	-3.27787200	-3.43856600	-2.66975400
H	-3.72509500	-4.73415100	-1.56967100
H	1.74185800	8.38651900	0.57377600
H	2.86323500	7.14944200	0.00473800
H	3.01666400	8.80574400	-0.58098700
H	2.38179800	5.83223800	3.31480600
H	1.33894900	4.55800300	2.66975400
H	2.23734800	5.59310200	1.56967100
H	-1.74185800	8.38651900	-0.57377600
H	-2.86323500	7.14944200	-0.00473800
H	-3.01666400	8.80574400	0.58098700
H	-2.38179800	5.83223800	-3.31480600
H	-1.33894900	4.55800300	-2.66975400
H	-2.23734800	5.59310200	-1.56967100
H	4.75998100	-6.05435500	0.00473800
H	6.11766600	-7.01538000	-0.58098700
H	6.39201000	-5.70175300	0.57377600
H	7.62321600	-1.09508700	-0.00473800
H	9.13433000	-1.79036400	0.58098700
H	8.13386800	-2.68476700	-0.57377600
H	6.24176500	-0.85342100	-3.31480600
H	4.61682100	-1.11943700	-2.66975400
H	5.96244300	-0.85895100	-1.56967100
B	1.08675500	8.93210500	-5.17539400
C	-0.11882400	9.17608300	-6.17686700
C	2.56156900	9.42600700	-5.48616100
C	-0.06470900	8.71402500	-7.52607100
C	-1.29573600	9.85917900	-5.74576800
C	2.83950700	10.79869000	-5.76078600
C	3.65192800	8.50466100	-5.48942900
C	-1.15437900	8.92286700	-8.38652600
C	1.13373200	7.95969500	-8.07124100
C	-2.35507700	10.07245800	-6.64281100
C	-1.45674600	10.40156300	-4.33468100
C	4.15704500	11.21416700	-6.01164800
C	1.75192800	11.85677600	-5.75837100
C	4.95270900	8.95312300	-5.77052800
C	3.46927900	7.01981900	-5.21860700
C	-2.29383200	9.60193000	-7.95436000
H	-1.09895400	8.55407900	-9.40691600
H	2.06045700	8.53046100	-7.96065900
H	1.28145700	7.00092200	-7.55771100
H	0.99764000	7.73842900	-9.13456700
H	-3.23354600	10.61448300	-6.30311100
H	-2.18216000	11.22209300	-4.32203900
H	-1.81939000	9.62811000	-3.64704500
H	-0.52024300	10.77950400	-3.91544000
C	5.21103400	10.30036600	-6.02269900
H	4.35034600	12.26511800	-6.20750100
H	0.94654600	11.61654200	-6.45869600
H	1.29491400	11.97089400	-4.76702700
H	2.16352800	12.83174600	-6.03788400
H	5.76660700	8.23336700	-5.79084300
H	4.29180100	6.44783400	-5.66133600
H	3.45826100	6.80727600	-4.14292300
H	2.53378200	6.62632000	-5.62600700
H	-3.12303100	9.76874800	-8.63538100
H	6.22287200	10.63412200	-6.23237400
B	7.19205200	-5.40721000	-5.17539400
C	8.00613300	-4.48513600	-6.17686700
C	6.88237700	-6.93138800	-5.48616100
C	7.57892100	-4.30097300	-7.52607100
C	9.18616700	-3.80745000	-5.74576800
C	7.93218600	-7.85843100	-5.76078600
C	5.53928800	-7.41499300	-5.48942900
C	8.30461900	-3.46171200	-8.38652600
C	6.32643200	-4.96168800	-8.07124100
C	9.90054300	-2.99667300	-6.64281100
C	9.73639100	-3.93920300	-4.33468100
C	7.63323000	-9.20719000	-6.01164800
C	9.39230500	-7.44560200	-5.75837100
C	5.27727800	-8.76573300	-5.77052800
C	4.34470200	-6.51439400	-5.21860700
C	9.46243100	-2.81444900	-7.95436000
H	7.95752700	-3.32531700	-9.40691600
H	6.35736700	-6.04963800	-7.96065900
H	5.42224800	-4.61023500	-7.55771100
H	6.20285700	-4.73319600	-9.13456700
H	10.80918500	-2.50690800	-6.30311100
H	10.80969800	-3.72124100	-4.32203900
H	9.24788300	-3.23841700	-3.64704500
H	9.59544600	-4.93920800	-3.91544000
C	6.31486100	-9.66307100	-6.02269900
H	8.44673100	-9.90006900	-6.20750100
H	9.58694700	-6.62800400	-6.45869600
H	9.71964100	-7.10687600	-4.76702700
H	10.03085400	-8.28954300	-6.03788400
H	4.24700200	-9.11071200	-5.79084300
H	3.43808800	-6.94072600	-5.66133600
H	4.16614400	-6.39858000	-4.14292300
H	4.47167100	-5.50747900	-5.62600700
H	10.02150000	-2.17975000	-8.63538100
H	6.09798400	-10.70622700	-6.23237400
B	-8.27880700	-3.52489500	-5.17539400
C	-7.88730800	-4.69094600	-6.17686700
C	-9.44394600	-2.49461900	-5.48616100
C	-7.51421200	-4.41305200	-7.52607100
C	-7.89043200	-6.05172900	-5.74576800
C	10.77169300	-2.94025900	-5.76078600
C	-9.19121600	-1.08966800	-5.48942900
C	-7.15024000	-5.46115500	-8.38652600
C	-7.46016400	-2.99800700	-8.07124100
C	-7.54546600	-7.07578500	-6.64281100
C	-8.27964500	-6.46236100	-4.33468100
C	11.79027600	-2.00697600	-6.01164800
C	11.14423300	-4.41117300	-5.75837100

C	10.22998600	-0.18739000	-5.77052800
C	-7.81398200	-0.50542600	-5.21860700
C	-7.16860000	-6.78748200	-7.95436000
H	-6.85857300	-5.22876200	-9.40691600
H	-8.41782400	-2.48082200	-7.96065900
H	-6.70370500	-2.39068700	-7.55771100
H	-7.20049600	-3.00523300	-9.13456700
H	-7.57563900	-8.10757500	-6.30311100
H	-8.62753800	-7.50085200	-4.32203900
H	-7.42849300	-6.38969300	-3.64704500
H	-9.07520300	-5.84029600	-3.91544000
C	11.52589500	-0.63729500	-6.02269900
H	12.79707700	-2.36504900	-6.20750100
H	10.53349400	-4.98853800	-6.45869600
H	11.01455500	-4.86401800	-4.76702700
H	12.19438200	-4.54220300	-6.03788400
H	10.01360900	0.87734400	-5.79084300
H	-7.72988900	0.49289200	-5.66133600
H	-7.62440500	-0.40869600	-4.14292300
H	-7.00545200	-1.11884100	-5.62600700
H	-6.89846900	-7.58899800	-8.63538100
H	12.32085600	0.07210500	-6.23237400
B	-1.08675500	8.93210500	5.17539400
C	0.11882400	9.17608300	6.17686700
C	-2.56156900	9.42600700	5.48616100
C	0.06470900	8.71402500	7.52607100
C	1.29573600	9.85917900	5.74576800
C	-2.83950700	10.79869000	5.76078600
C	-3.65192800	8.50466100	5.48942900
C	1.15437900	8.92286700	8.38652600
C	-1.13373200	7.95969500	8.07124100
C	2.35507700	10.07245800	6.64281100
C	1.45674600	10.40156300	4.33468100
C	-4.15704500	11.21416700	6.01164800
C	-1.75192800	11.85677600	5.75837100
C	-4.95270900	8.95312300	5.77052800
C	-3.46927900	7.01981900	5.21860700
C	2.29383200	9.60193000	7.95436000
H	1.09895400	8.55407900	9.40691600
H	-2.06045700	8.53046100	7.96065900
H	-1.28145700	7.00092200	7.55771100
H	-0.99764000	7.73842900	9.13456700
H	3.23354600	10.61448300	6.30311100
H	2.18216000	11.22209300	4.32203900
H	1.81939000	9.62811000	3.64704500
H	0.52024300	10.77950400	3.91544000
C	-5.21103400	10.30036600	6.02269900
H	-4.35034600	12.26511800	6.20750100
H	-0.94654600	11.61654200	6.45869600
H	-1.29491400	11.97089400	4.76702700
H	-2.16352800	12.83174600	6.03788400
H	-5.76660700	8.23336700	5.79084300
H	-4.29180100	6.44783400	5.66133600
H	-3.45826100	6.80727600	4.14292300
H	-2.53378200	6.62632000	5.62600700
H	3.12303100	9.76874800	8.63538100
H	-6.22287200	10.63412200	6.23237400
B	-7.19205200	-5.40721000	5.17539400
C	-8.00613300	-4.48513600	6.17686700
C	-6.88237700	-6.93138800	5.48616100
C	-7.57892100	-4.30097300	7.52607100
C	-9.18616700	-3.80745000	5.74576800
C	-7.93218600	-7.85843100	5.76078600
C	-5.53928800	-7.41499300	5.48942900
C	-8.30461900	-3.46171200	8.38652600
C	-6.32643200	-4.96168800	8.07124100
C	-9.90054300	-2.99667300	6.64281100
C	-9.73639100	-3.93920300	4.33468100
C	-7.63323000	-9.20719000	6.01164800
C	-9.39230500	-7.44560200	5.75837100
C	-5.27727800	-8.76573300	5.77052800
C	-4.34470200	-6.51439400	5.21860700
C	-9.46243100	-2.81444900	7.95436000
H	-7.95752700	-3.32531700	9.40691600
H	-6.35736700	-6.04963800	7.96065900
H	-5.42224800	-4.61023500	7.55771100
H	-6.20285700	-4.73319600	9.13456700
H	10.80918500	-2.50690800	6.30311100
H	10.80969800	-3.72124100	4.32203900
H	-9.24788300	-3.23841700	3.64704500
H	-9.59544600	-4.93920800	3.91544000
C	-6.31486100	-9.66307100	6.02269900
H	-8.44673100	-9.90006900	6.20750100
H	-9.58694700	-6.62800400	6.45869600
H	-9.71964100	-7.10687600	4.76702700
H	10.03085400	-8.28954300	6.03788400
H	-4.24700200	-9.11071200	5.79084300
H	-3.43808800	-6.94072600	5.66133600
H	-4.16614400	-6.39858000	4.14292300
H	-4.47167100	-5.50747900	5.62600700
H	10.02150000	-2.17975000	8.63538100
H	-6.09798400	-10.70622700	6.23237400
B	8.27880700	-3.52489500	5.17539400
C	7.88730800	-4.69094600	6.17686700
C	9.44394600	-2.49461900	5.48616100
C	7.51421200	-4.41305200	7.52607100
C	7.89043200	-6.05172900	5.74576800
C	10.77169300	-2.94025900	5.76078600
C	9.19121600	-1.08966800	5.48942900
C	7.15024000	-5.46115500	8.38652600
C	7.46016400	-2.99800700	8.07124100
C	7.54546600	-7.07578500	6.64281100
C	8.27964500	-6.46236100	4.33468100
C	11.79027600	-2.00697600	6.01164800
C	11.14423300	-4.41117300	5.75837100
C	10.22998600	-0.18739000	5.77052800
C	7.81398200	-0.50542600	5.21860700
C	7.16860000	-6.78748200	7.95436000
H	6.85857300	-5.22876200	9.40691600
H	8.41782400	-2.48082200	7.96065900
H	6.70370500	-2.39068700	7.55771100
H	7.20049600	-3.00523300	9.13456700
H	7.57563900	-8.10757500	6.30311100
H	8.62753800	-7.50085200	4.32203900
H	7.42849300	-6.38969300	3.64704500
H	9.07520300	-5.84029600	3.91544000
C	11.52589500	-0.63729500	6.02269900
H	12.79707700	-2.36504900	6.20750100
H	10.53349400	-4.98853800	6.45869600
H	11.01455500	-4.86401800	4.76702700
H	12.19438200	-4.54220300	6.03788400
H	10.01360900	0.87734400	5.79084300
H	7.72988900	0.49289200	5.66133600
H	7.62440500	-0.40869600	4.14292300
H	7.00545200	-1.11884100	5.62600700
H	6.89846900	-7.58899800	8.63538100
H	12.32085600	0.07210500	6.23237400

References

[1] R. Uson, L. A. Oro, J. A. Cabeza, H. E. Bryndza, M. P. Stepro, *Inorg. Synth.* **1985**, *23*, 126-130.

[2] E. Zysman-Colman, K. Arias, J. S. Siegel, *Can. J. Chem.* **2009**, *87*, 440-447.

[3] D. Landini, H. Molinari, M. Pensa, A. Rampoldi, *Synthesis* **1988**, 953-955.

[4] A. Narsaria, F. Rauch, J. Krebs, P. Endres, A. Friedrich, I. Krummenacher, H. Braunschweig, M. Finze, J. Nitsch, F. M. Bickelhaupt, T. B. Marder, *Adv. Funct. Mater.* **2020**, accepted for publication March 2020.

[5] R. T. Hawkins, W. J. Lennarz, H. R. Snyder, *J. Am. Chem. Soc.* **1960**, *82*, 3053-3059.

[6] A. Krasovskiy, P. Knochel, *Synthesis* **2006**, 890-891.

[7] A. Ito, K. Kawanishi, E. Sakuda, N. Kitamura, *Chem. Eur. J.* **2014**, *20*, 3940-3953.

[8] G. R. Fulmer, A. J. M. Miller, N. H. Sherden, H. E. Gottlieb, A. Nudelman, B. M. Stoltz, J. E. Bercaw, K. I. Goldberg, *Organometallics* **2010**, *29*, 2176-2179.

[9] G. Sheldrick, *Acta Crystallogr. A* **2015**, *71*, 3-8.

[10] G. Sheldrick, *Acta Crystallogr. A* **2008**, *64*, 112-122.

[11] C. B. Hübschle, G. M. Sheldrick, B. Dittrich, *J. Appl. Crystallogr.* **2011**, *44*, 1281-1284.

[12] A. Spek, *Acta Crystallogr. C* **2015**, *71*, 9-18.

[13] Diamond, 4.6.0, K. Brandenburg, Crystal Impact H. Putz & K. Brandenburg GbR, Bonn (Germany), **2017**.

[14] O. V. Dolomanov, L. J. Bourhis, R. J. Gildea, J. A. K. Howard, H. Puschmann, *J. Appl. Crystallogr.* **2009**, *42*, 339-341.

[15] Gaussian 09 Revision E.01, M. J. Frisch, G. W. Trucks, H. B. Schlegel, G. E. Scuseria, M. A. Robb, J. R. Cheeseman, G. Scalmani, V. Barone, B. Mennucci, G. A. Petersson, H. Nakatsuji, M. Caricato, X. Li, H. P. Hratchian, A. F. Izmaylov, J. Bloino, G. Zheng, J. L. Sonnenberg, M. Hada, M. Ehara, K. Toyota, R. Fukuda, J. Hasegawa, M. Ishida, T. Nakajima, Y. Honda, O. Kitao, H. Nakai, T. Vreven, J. A. Montgomery, J. E. Peralta, F. Ogliaro, M. Bearpark, J. J. Heyd, E. Brothers, K. N. Kudin, V. N. Staroverov, R. Kobayashi, J. Normand, K. Raghavachari, A. Rendell, J. C. Burant, S. S. Iyengar, J. Tomasi, M. Cossi, N. Rega, J. M. Millam, M. Klene, J. E. Knox, J. B. Cross, V. Bakken, C. Adamo, J. Jaramillo, R. Gomperts, R. E. Stratmann, O. Yazyev, A. J. Austin, R. Cammi, C. Pomelli, J. W. Ochterski, R. L. Martin, K. Morokuma, V. G. Zakrzewski, G. A. Voth, P. Salvador, J. J. Dannenberg, S. Dapprich, A. D. Daniels, Farkas, J. B. Foresman, J. V. Ortiz, J. Cioslowski, D. J. Fox, Gaussian Inc., Wallingford CT, **2016**.

[16] M. D. Hanwell, D. E. Curtis, D. C. Lonie, T. Vandermeersch, E. Zurek, G. R. Hutchison, *J. Cheminformatics* **2012**, *4*, 17.

[17] T. Lu, F. Chen, *J. Comput. Chem.* **2012**, *33*, 580-592.

[18] C. Lee, W. Yang, R. G. Parr, *Phys. Rev. B: Condens. Matter Mater. Phys.* **1988**, *37*, 785-789.

[19] G. A. Petersson, M. A. Al-Laham, *J. Chem. Phys.* **1991**, *94*, 6081-6090.

[20] G. A. Petersson, A. Bennett, T. G. Tensfeldt, M. A. Al-Laham, W. A. Shirley, J. Mantzaris, *J. Chem. Phys.* **1988**, *89*, 2193-2218.
